# FUCA2 Sustains AKT Signaling and Suppresses Senescence by Antagonizing FUT3‐Mediated ErbB3 Fucosylation in Lung Adenocarcinoma

**DOI:** 10.1002/advs.202523667

**Published:** 2026-06-16

**Authors:** Lu Chen, Jintao Guo, Fei Li, Mingjie Gao, Runyang Li, Zhaozhang Huang, Yaolin Zheng, Chunyi Gao, Jihuan Hou, Qiang Yu, Bowen Zheng, Xuemei Chen, Wenqing Zhang, Xiaoting Hong, Yali Zheng, Daxuan Wang, Qiyuan Li, Tianhui Hu, Yan‐yan Zhan

**Affiliations:** ^1^ Cancer Research Center School of Medicine Xiamen University Xiamen China; ^2^ National Institute for Data Science in Health and Medicine School of Medicine Xiamen University Xiamen China; ^3^ Shandong Provincial Key Medical and Health Laboratory of BT and IT for Thoracic Oncology The First Affiliated Hospital of Shandong Second Medical University Weifang China; ^4^ Department of Respiratory Critical Care and Sleep Medicine School of Medicine Xiang'an Hospital of Xiamen University Xiamen University Xiamen China; ^5^ Department of Oncology School of Medicine Zhongshan Hospital of Xiamen University Xiamen University Xiamen China; ^6^ School of Food Science and Technology Jiangnan University Wuxi China; ^7^ Department of Respiratory Medicine, Fuzhou University Affiliated Provincial Hospital Fujian Provincial Hospital Fuzhou China

**Keywords:** AKT signaling, ErbB3 fucosylation, FUCA2, lung adenocarcinoma, senescence

## Abstract

While targeted therapies have improved outcomes in lung adenocarcinoma (LUAD), many patients still lack targetable mutations. Here, we identified alpha‐L‐fucosidase 2 (FUCA2) as a crucial driver of LUAD by preventing cellular senescence. Mechanistically, through the restriction of fucosyltransferase 3 (FUT3)‐mediated α‐1,3‐fucosylation of ErbB3 at Asn437, FUCA2 safeguarded ErbB3–ErbB2 heterodimerization to promote ErbB3 activation and sustain persistent AKT signaling. Active AKT prevented p53 protein stabilization in TP53‐wild‐type LUAD cells and inhibited p27 protein accumulation in TP53‐mutant LUAD cells, thereby counteracting senescence and supporting malignant growth. Notably, low‐dose Capivasertib, an AKT inhibitor targeting tumors with PIK3CA/AKT1/PTEN mutation(s), induced senescence selectively in FUCA2‐high LUAD irrespective of PIK3CA/AKT1/PTEN/TP53 mutational status, and its combination with the nutraceutical senolytic procyanidin C1 achieved potent and low‐toxicity suppression of LUAD across multiple preclinical models. Together, our results uncover the FUCA2–ErbB3 fucosylation–AKT pathway as a central regulator of senescence and propose a FUCA2‐guided drug repurposing strategy for LUAD.

## Graphical abstract

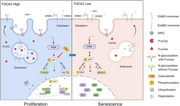

## 1 Introduction

Lung cancer is the malignancy with the highest incidence and mortality rate worldwide [1]. Lung adenocarcinoma (LUAD), the predominant histological subtype, accounts for approximately 40% of primary pulmonary malignancies. Recent advances in targeted therapies, exemplified by EGFR‐tyrosine kinase inhibitors (EGFR‐TKIs) such as gefitinib and osimertinib, have markedly improved clinical outcomes in LUAD [2, 3]. Compared with conventional cytotoxic chemotherapy, targeted agents offer greater precision, reduced systemic toxicity, and improved tolerability. However, a substantial subset of LUAD patients lacking targetable mutations or dysregulated expression in known driver genes remains refractory to current targeted therapies. Elucidating the molecular pathogenesis of LUAD and identifying novel oncogenic drivers are therefore critical for advancing targeted therapies and enhancing patient survival.

N‐linked glycosylation serves as a critical post‐translational modification (PTM) that precisely regulates protein localization, functional activation, and degradation dynamics [4, 5, 6]. Among its diverse modifications, fucosylation plays a pivotal role in determining N‐glycan architecture by controlling branch extension and terminal elaboration [7, 8, 9, 10]. This process is mediated by Golgi‐resident fucosyltransferases (FUT1–11), which catalyze the transfer of L‐fucose to N‐glycans and glycolipids through distinct α‐1,2‐, α‐1,3‐, α‐1,4‐ or α‐1,6‐linkages [10, 11]. Conversely, fucose removal is executed by GH29 fucosidases, with the GH29A subfamily (FUCA1/2) specifically targeting α‐1,2/3/4/6‐fucosyl moieties across glycoconjugate classes [12, 13]. Fucosylation of glycoconjugates acts as a fundamental regulatory mechanism governing mammalian physiology, with demonstrated roles in immunocyte migration, host defense, reproductive biology, and ontogeny [14]. Dysregulated fucosylation, driven by altered expression of fucosyltransferases or fucosidases, is implicated in diverse human diseases spanning cystic fibrosis to malignancy [14, 15].

Aberrant fucosylation is a hallmark of cancer, driving tumorigenesis, metastasis, and immune evasion [11, 15, 16, 17]. FUT8, a core fucosyltransferase overexpressed in non‐small cell lung cancer (NSCLC), colorectal cancer, and triple‐negative breast cancer, promotes tumor growth by modifying key targets including TGFBR1, EGFR, and immune checkpoints (B7‐H3, PD‐1) [18, 19]. FUT3, which catalyzes α‐1,3/α‐1,4 fucosylation, is also upregulated in LUAD and rewires glucose metabolism via NF‐κB [20]. Intriguingly, FUCA2, which antagonizes both FUT8 and FUT3, is similarly upregulated in LUAD and predicts adverse clinical outcomes [21, 22]. This paradoxical observation underscores the multifaceted regulatory roles of fucosylation in tumorigenesis. Thus, systematic dissection of FUCA2's oncogenic mechanisms in LUAD is critical for therapeutic development.

Here, we identified FUCA2 as a senescence‐related oncogenic driver in LUAD via integrated analysis of The Cancer Genome Atlas (TCGA)‐LUAD datasets. FUCA2 ablation enhanced FUT3‐mediated α‐1,3‐fucosylation of ErbB3 at Asn437, disrupting ErbB3–ErbB2 dimerization and AKT signaling. Consequent AKT inactivation stabilized p53 (in TP53‐wild‐type cells) or p27 (in TP53‐mutant cells), triggering senescence. We further repurposed low‐dose Capivasertib (an FDA‐approved AKT inhibitor) to induce senescence specifically in FUCA2‐high LUAD cells, independent of PIK3CA/AKT1/PTEN/TP53 mutation status. Combining Capivasertib with the senolytic procyanidin C1 achieved potent, low‐toxicity LUAD suppression in vitro and in vivo. Together, our findings establish the FUCA2‐ErbB3 fucosylation‐AKT axis as a senescence regulator and support a FUCA2‐guided repurposing strategy (low‐dose Capivasertib plus procyanidin C1) for LUAD therapy.

## 2 Results

### 2.1 FUCA2 Was a Driver Oncogene Negatively Associated With Senescence in LUAD

**FIGURE 1:**
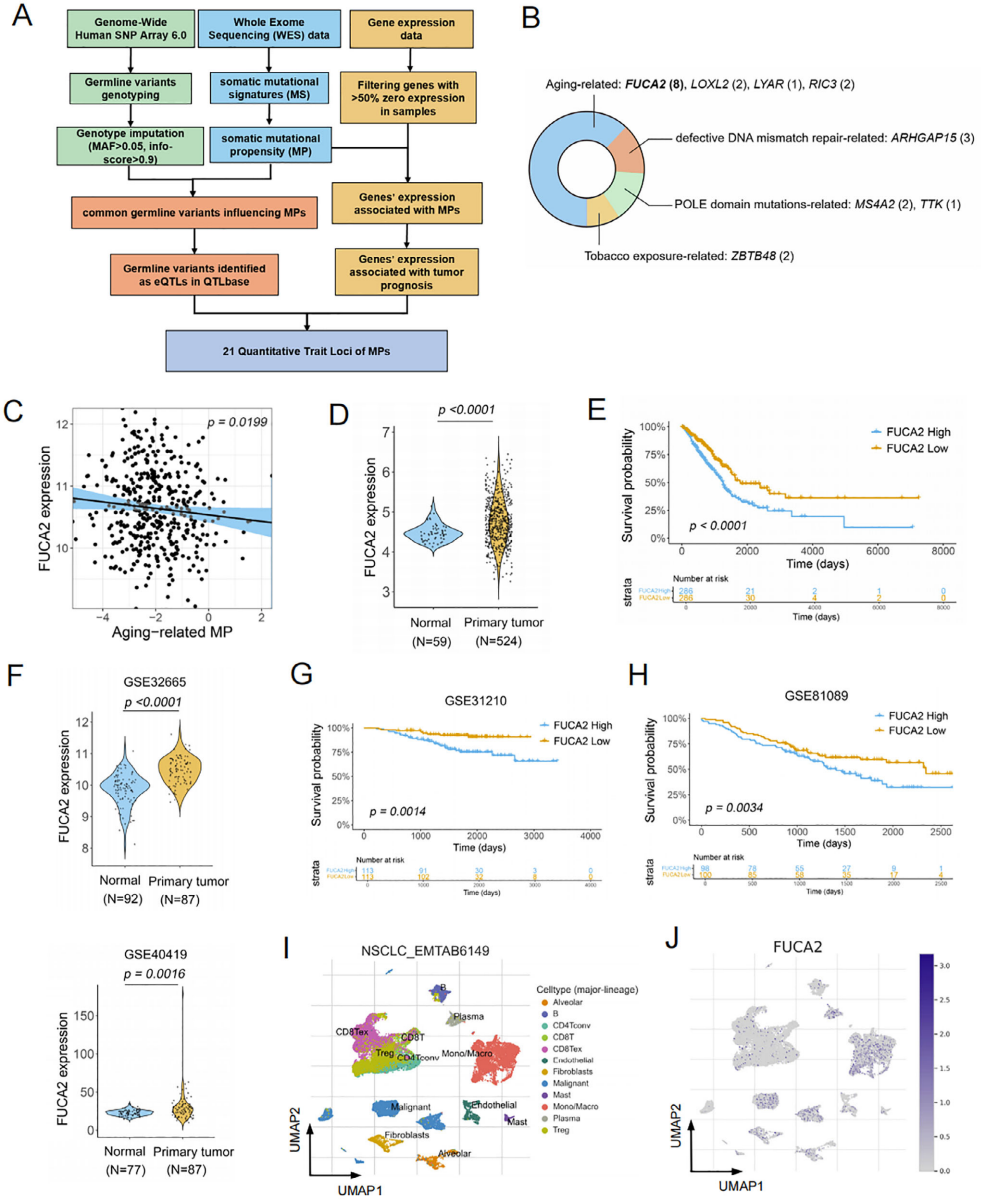
Systematic bioinformatics screening identified FUCA2 as a novel potential driver gene inversely correlated with cellular senescence in LUAD. (A) Workflow for identifying 21 quantitative trait loci (QTLs) linked to somatic mutational processes in LUAD, integrating SNP6.0 array (Affymetrix Genome‐Wide Human SNP Array 6.0), whole‐exome sequencing (WES) somatic mutation data, and gene expression profiles. (B) Distribution patterns of 4 somatic mutation signatures and their associated genes including FUCA2. (C) Negative correlation between FUCA2 expression and the senescence‐associated somatic mutation gene set. (D, E) TCGA LUAD dataset analyses of clinical relevance of FUCA2 and FUCA1: FUCA2 expression was significantly higher in tumor tissues than in adjacent normal tissues (D); High FUCA2 expression correlated with poorer survival outcomes (E). (F–H), Independent validation of FUCA2 overexpression and its prognostic impact in LUAD using GEO database: GSE32665 and GSE40419 datasets confirmed FUCA2 upregulation in LUAD (F); GSE31210 and GSE81089 datasets supported FUCA2 as a prognostic marker for unfavorable outcomes (G, H). (I, J) Single‐cell RNA sequencing revealed FUCA2 expression patterns across NSCLC cell populations: Cell type annotation in the NSCLC_EMTAB6149 single‐cell dataset (I, from TISCH database, available at http://tisch.compbio.cn/home/); FUCA2 expression profile across distinct cell types (I, from TISCH database, available at http://tisch.compbio.cn/home/).

To identify potential therapeutic targets for LUAD, we initially explored the potential driver genes using data from the TCGA database. Utilizing SNP6.0 chip data (Affymetrix Genome‐Wide Human SNP Array 6.0), whole exome sequencing (WES) somatic mutation data, and gene expression data, we identified 6 103 818 common germline variants (minimum allele frequency (MAF) > 0.05) alongside six somatic mutation signatures: MS1 (aging‐related), MS2 (linked to the activity of the AID/APOBEC cytidine deaminase family), MS3 (defective DNA mismatch repair‐related), MS4 (tobacco exposure‐related), and MS5 and MS6 (polymerase epsilon exonuclease (POLE) domain mutations‐related) (Figure 1A). The screening were then conducted in three steps: (1) We correlated germline variants with somatic mutation signatures to identify germline loci influencing the somatic mutation process, subsequently using eQTLbase (expression quantitative trait locus database) to determine the genes affected by these loci; (2) Simultaneously, we performed an association analysis between gene expression and somatic mutation signatures to identify genes significantly correlated with the somatic mutation process and further screened for those linked to tumor prognosis; (3) Finally, we sorted out the intersection of the two gene sets mentioned above (Figure 1A). In total, we identified 21 expression quantitative trait loci (eQTLs) related to the somatic mutation process (*p* < 1 × 10^−5^), which were significantly associated with eight genes including *FUCA2*, *LOXL2*, *LYAR*, *RIC3*, *ARHGAP15*, *MS4A2*, *TTK*, and *ZBTB48* (Table S1). Notably, 13 of the 21 mpQTLs were linked to aging‐related somatic mutational signatures (Figure 1B; Table S1). Particularly, the *FUCA2* gene exhibited the strongest (showing eight eQTLs) correlation with the aging‐related somatic mutation process, and the mRNA expression of *FUCA2* was negatively associated with the activity of the aging‐related somatic mutational signature (Figure 1B,C; Figure S1A and Table S1).

Furthermore, FUCA2 was found to be overexpressed and significantly correlated with poor prognostic outcomes in the LUAD dataset from the TCGA database (Figure 1D,E). In contrast, *FUCA1*, a gene from the same family, did not demonstrate a significant difference in expression and was not associated with prognosis in LUAD (Figure S1B,C). Using datasets GSE32665 and GSE40419 from the Gene Expression Omnibus (GEO) database, we validated that FUCA2 had higher expression levels in LUAD tissue compared to adjacent tissue (Figure 1F). Additionally, we confirmed the correlation between the expression level of FUCA2 and LUAD patient prognosis using datasets GSE31210 and GSE81089 from the GEO database, while FUCA1's expression level showed no statistically significant difference in relation to LUAD patient outcomes (Figure 1G,H; Figure S1D,E).

Notably, as indicated by bioinformatics analysis using single‐cell sequencing data (NSCLC_EMTAB6149) in the EMBL‐EBI database, FUCA2 were predominantly expressed in malignant cells and monocytes/macrophages rather than other cell types within non‐small cell lung cancer (NSCLC, with lung adenocarcinoma as its predominant subtype accounting for approximately 40%–50% of cases) samples (Figure 1I,J), highlighting the potential role of FUCA2 in LUAD cells.

### 2.2 FUCA2 Knockdown Induced Cellular Senescence to Suppress LUAD Growth In Vitro and In Vivo

**FIGURE 2:**
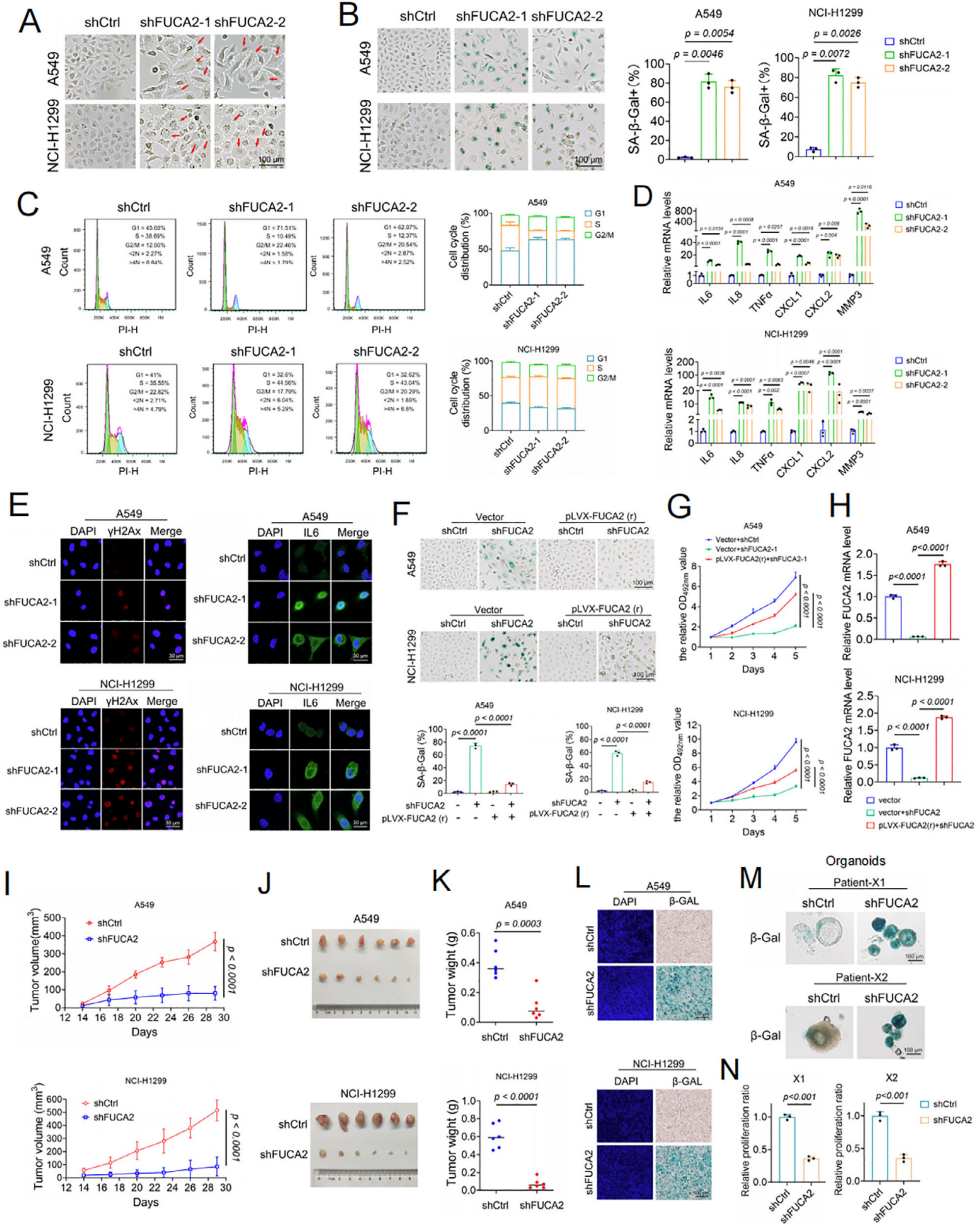
FUCA2 knockdown suppressed proliferation via inducing senescence in LUAD cells in vitro and in vivo. (A) Morphological changes characteristic of senescence (flattened and enlarged cells, indicated by red arrow) following FUCA2 deletion. The white dashed line outlined the outer contour of the cells with morphological changes. Scale bar: 100 µm. (B) SA‐β‐gal staining of FUCA2‐depleted cells (n = 300 cells analyzed across three biological replicates). Scale bar: 100 µm. (C) Cell cycle distribution analysis by propidium iodide staining and flow cytometry after FUCA2 knockdown. (D) Real‐time PCR analysis showing significant upregulation of SASP factors in FUCA2‐depleted cells. (E) Immunofluorescence detection of increased γH2AX foci (red) and IL6 expression (green) in FUCA2 knockdown cells (DAPI, blue). Scale bar: 30 µm. (F–H) Rescue experiments with FUCA2 overexpression (pLVX‐FUCA2(r), a shRNA‐resistant construct)) ahead of FUCA2 knockdown: Reversal of senescence phenotype by SA‐β‐gal staining (F, scale bar: 100 µm); Restoration of proliferative capacity by MTT assay (G); Confirmation of FUCA2 re‐expression by real‐time PCR (H). (I–L) Subcutaneous xenograft models utilizing A549 and NCI‐H1299 cells showed reduced tumor growth upon FUCA2 knockdown: Tumor volume measurements over time (I, n = 6 per group); Photograph (J) and weight (K) of tumors at endpoint (n = 6 per group); SA‐β‐gal staining of tumor sections revealed significantly increased senescence in shFUCA2 tumors versus controls (L, scale bar: 100 µm). (M, N) FUCA2 knockdown induced cellular senescence (M, SA‐β‐gal staining, scale bar: 100 µm) and decreased proliferation (N, ATP viability assay) in LUAD organoids derived from two patients (X1 and X2). Data represented the mean + or ± SEM, and the *p* values were analyzed by one‐way ANOVA (B, D, F, G‐I) or unpaired two‐tailed Student's *t*‐test (K, N).

The induction of senescence in preneoplastic cells limits cancer initiation, and many cancer therapies function, at least in part, by inducing senescence in cancer cells [23, 24, 25]. Since the tumor suppressor gene *TP53* is often inactivated by mutation in LUAD, two human LUAD cell lines A549 (with wild‐type p53) and NCI‐H1299 (p53‐deficient) were utilized to investigate the effects of FUCA2 on growth and senescence of LUAD cells with or without functional p53. Results from the MTT assay and colony formation assay showed that knocking down FUCA2, with short hairpin RNA against FUCA2 (shFUCA2), significantly inhibited proliferation in both A549 and NCI‐H1299 cell lines (Figure S2A–C). Microscopic examination revealed that after FUCA2 knockdown, A549 and NCI‐H1299 cells exhibited abnormal enlargement and a flattened appearance, displaying typical cellular senescence morphology (Figure 2A). Further analysis using common indicators of cellular senescence, including β‐galactosidase (SA‐β‐gal) staining, cell cycle analysis, senescence‐associated secretory phenotype (SASP) assessment, and γH2AX/IL6 staining, confirmed that knocking down FUCA2 induced senescence in LUAD cells (Figure 2B–E). Moreover, the introduction of a synonymous FUCA2 mutant (resistant to shFUCA2 interference, named FUCA2(r)) into A549 and NCI‐H1299 cells with FUCA2 knockdown beforehand failed to reverse senescence and regrowth (Figure S2D,E), suggesting that even short‐term FUCA2 deficiency resulted in irreversible senescence and cell growth arrest in LUAD cells. However, overexpression of FUCA2(r) ahead of FUCA2 knockdown effectively attenuated the senescence and cell growth inhibition caused by FUCA2 knockdown (Figure 2F–H), confirming the critical regulatory role of FUCA2 in LUAD cell senescence and proliferation. Notably, at the early stage of FUCA2 knockdown (around day 3, when a significant number of cells had already undergone senescence), no significant apoptosis was observed (Figure S2F). Interestingly, one week after the knockdown, a small proportion of cells (∼20%) underwent apoptosis (Figure S2G). Therefore, these results indicated that FUCA2 knockdown induced senescence, with a small probability of spontaneous conversion to apoptosis, to inhibit the growth in human LUAD A549 and NCI‐H1299 cells.

Next, we employed a xenograft mouse model to verify the effects of FUCA2 knockdown on LUAD cell senescence and growth in vivo. Four stable cell sublines, including A549‐shCtrl, A549‐shFUCA2, NCI‐H1299‐shCtrl, and NCI‐H1299‐shFUCA2, were constructed and used to establish a nude mouse xenograft model via subcutaneous injection. The results showed that FUCA2 knockdown significantly inhibited the growth of subcutaneous xenograft tumors formed by A549 and NCI‐H1299 cells, without affecting the body weight of nude mice (Figure 2I–K; Figure S2H). Subsequent analysis of the xenograft tumor sections by β‐galactosidase staining and TUNEL staining indicated that FUCA2 knockdown triggered widespread senescence and scattered apoptosis in xenograft tumors derived from both A549 and NCI‐H1299 cell lines (Figure 2L; Figure S2I).

Patient‐derived organoids represent a transformative preclinical model that faithfully recapitulates human LUAD pathophysiology [26]. Using organoids derived from two treatment‐naive LUAD patients, we demonstrated that FUCA2 knockdown induced marked cellular senescence and significantly impaired proliferative capacity (Figure 2M,N; Figure S2J,K), establishing FUCA2 as a critical negative regulator of senescence in LUAD.

### 2.3 Knocking Down FUCA2 Induced Cell Senescence Through Stabilizing p53/p27 Proteins

**FIGURE 3:**
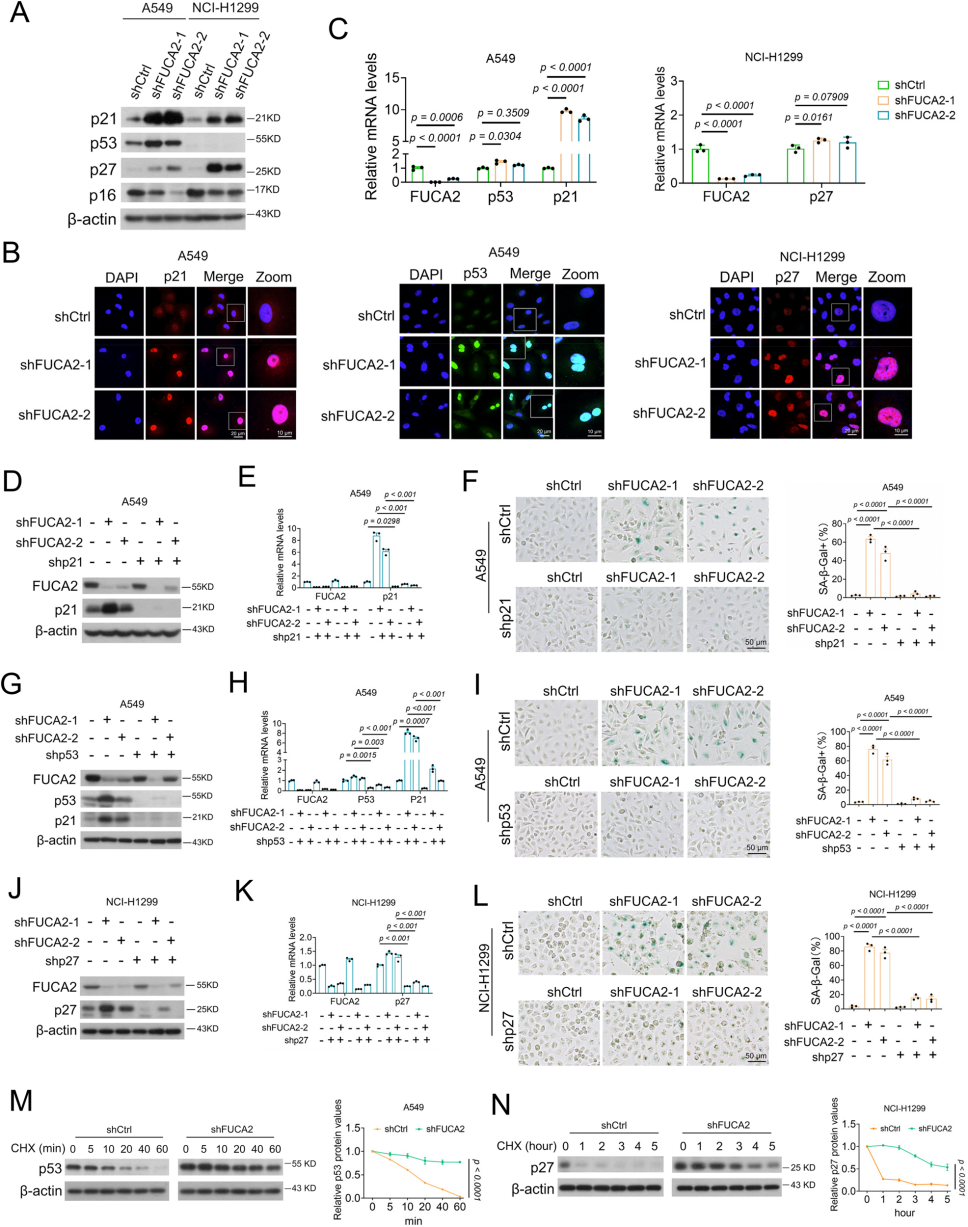
FUCA2 regulated cellular senescence through p53 and p27 protein stabilization in p53‐wild‐type and p53‐deficient LUAD cells, respectively. (A–C) Senescence marker profiling in FUCA2 knockdown cells: Western blots of p53, p21, p27, and p16 proteins (A); Real‐time PCR assays for p53, p21, and p27 mRNA levels (B); Immunofluorescence assay demonstrating nuclear accumulation of p21/p53 (red/green) in A549 (p53‐wild‐type) cells and p27 (red) in NCI‐H1299 (p53‐deficient) cells with DAPI nuclear staining (blue) (C, scale bars: 20 µm for main panels, 10 µm for insets). (D–F) FUCA2/p21 co‐knockdown effects in A549 cells were detected by western blotting (D), real‐time PCR (E), and SA‐β‐gal staining (F, scale bars: 50 µm); (G–I) FUCA2/p53 co‐knockdown effects in A549 cells were detected by western blotting (G), real‐time PCR (H), and SA‐β‐gal staining (I, scale bars: 50 µm). (J–L) FUCA2/p27 co‐knockdown effects in NCI‐H1299 cells were detected by western blotting (J), real‐time PCR (K), and SA‐β‐gal staining (L, scale bars: 50 µm). (M, N) Protein stability was detected by western blotting: p53 in A549 cells using cycloheximide (50 µg/mL) at 0–60 min (M) and p27 in NCI‐H1299 cells using cycloheximide (80 µg/mL) at 0–5 h (N). Data represented the mean + SEM, and the *p* values were analyzed by one‐way ANOVA (C, E, F, H, I, K, L) or unpaired two‐tailed Student's *t*‐test (M, N).

We then investigated how FUCA2 regulates senescence in LUAD cells. The effects of FUCA2 knockdown on the expression of senescence‐associated proteins p21, p53 (the upstream transcription factor of p21), p27, and p16 were tested in p53‐positive A549 cells and p53‐deficient NCI‐H1299 cells. The results from western blot, immunofluorescence, and real‐time PCR showed that FUCA2 knockdown in A549 cells dramatically upregulated both the mRNA and protein levels of p21 to a similar extent and substantially increased the protein level of p53 without significantly affecting its mRNA level; in NCI‐H1299 cells, FUCA2 knockdown greatly increased the protein level of p27 without notably altering its mRNA level (Figure 3A–C). Furthermore, in A549 cells, knockdown of p21 blocked the senescence induced by FUCA2 knockdown (Figure 3D–F). Additionally, knocking down p53 simultaneously blocked the promoting effects of FUCA2 knockdown on the mRNA and protein expression of p21 and cell senescence in A549 cells (Figure 3G–I). On the other hand, knockdown of p27 blocked the senescence induced by FUCA2 knockdown in NCI‐H1299 cells (Figure 3J–L). These results suggested that FUCA2 knockdown induced senescence in p53‐positive A549 and p53‐deficient NCI‐H1299 cells by elevating the protein levels (but not the mRNA levels) of p53 and p27, respectively.

We therefore asked whether FUCA2 knockdown upregulated the protein levels of p53 and p27 by stabilizing these proteins. To test this hypothesis, we treated both control and FUCA2 knockdown A549 and NCI‐H1299 cells with the protein synthesis inhibitor cycloheximide (CHX). As shown in Figure 3M,N, FUCA2 knockdown dramatically reduced the degradation rates of p53 and p27 proteins in A549 and NCI‐H1299 cells, respectively. Together, the above suggested that FUCA2 knockdown increased the stability of p53/p27 proteins in LUAD cells.

### 2.4 FUCA2 Regulation of FUT3‐Catalyzed Fucosylation Mediated Its Effects on p53/p27 Protein Stability

**FIGURE 4:**
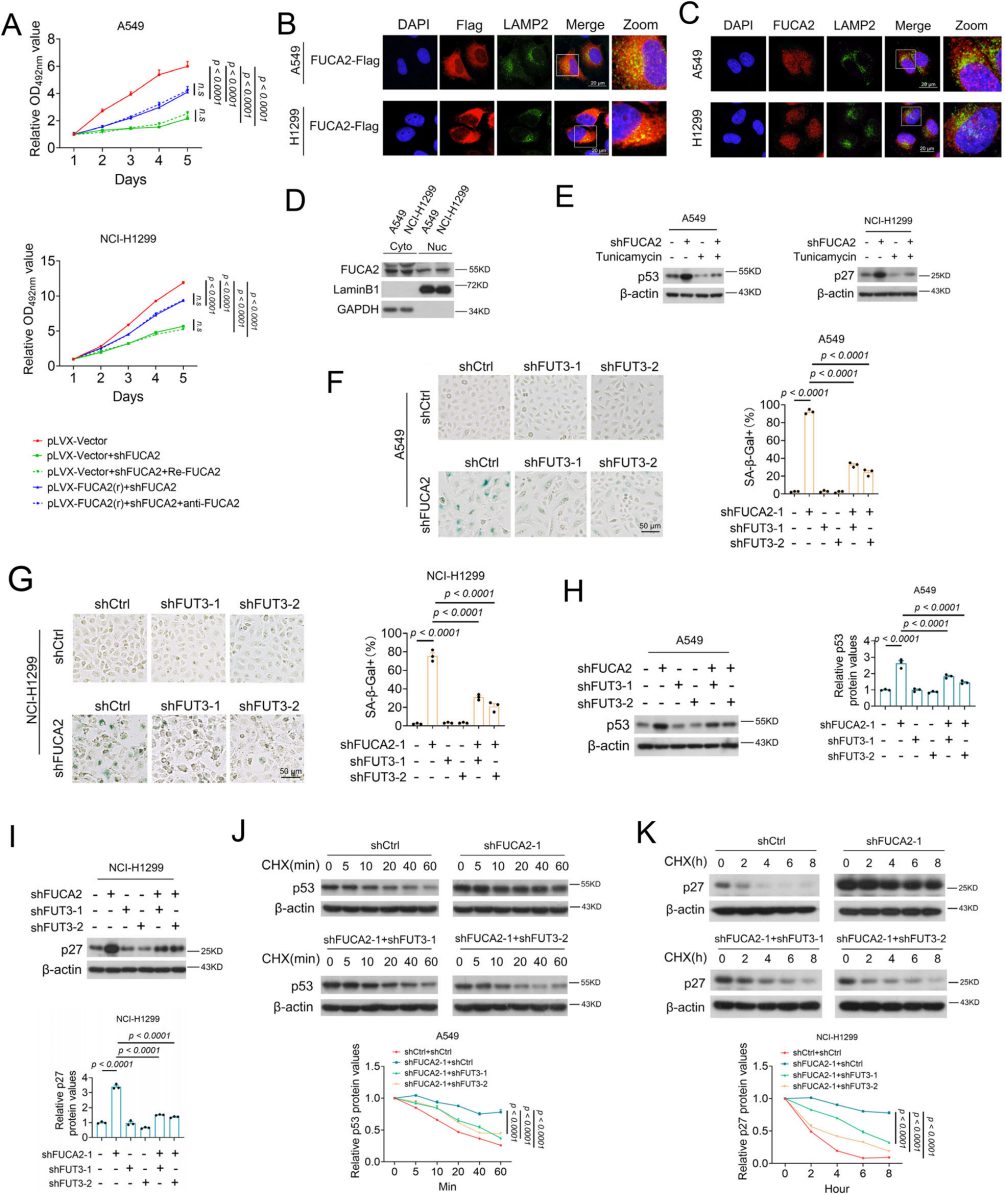
The tumor‐promoting effects of FUCA2 in lung adenocarcinoma were mediated through FUT3‐dependent α‐1,3‐fucosylation. (A) Intracellular (but not secreted) FUCA2 promoted proliferation of LUAD cells: recombinant human FUCA2 (Re‐FUCA2, 0.2 µg/mL, 5 days) failed to rescue proliferation defects in FUCA2‐knockdown cells, and FUCA2‐neutralizing antibody (0.2 µg/mL, 5 days) did not inhibit FUCA2 function. (B, C) Immunofluorescence revealed predominant cytoplasmic localization of exogenous (B) and endogenous (C) FUCA2 with partial lysosomal co‐localization (indicated by lysosome marker LAMP2) and minor nuclear distribution in both A549 and NCI‐H1299 cells (blue: DAPI; red: FUCA2; green: LAMP2; scale bars: 20 µm). (D) Western blots of nucleoplasmic fractionation confirmed endogenous FUCA2 distribution in cytoplasm and nucleus. (E) Tunicamycin (10 µM, 24 h) treatment abolished FUCA2 depletion‐induced p53/p27 accumulation. (F–K) FUT3‐dependent regulation by FUCA2: SA‐β‐gal staining in A549 (F) and NCI‐H1299 (G) cells; p53 protein levels in A549 (H) and p27 protein levels in NCI‐H1299 (I); p53 protein stabilization in A549 cells (CHX 50 µg/mL) (J) and p27 protein stabilization in NCI‐H1299 cells (CHX 80 µg/mL) (K). Data represented the mean + SEM, and the *p* values were analyzed by one‐way ANOVA (A, F–K).

We further investigated how FUCA2 affected p53/p27 proteins and LUAD cell senescence. It has been reported that FUCA2 functions as a fucosidase in lysosomes or a secreted non‐lysosomal enzyme [7, 27, 28]. The addition of anti‐FUCA2 antibody in culture medium showed no influence on the effect of FUCA2 on the growth of A549 and NCI‐H1299 cells (Figure 4A), suggesting that the regulation of LUAD by FUCA2 is independent of its extracellular enzyme functions. Immunofluorescence and nuclear‐cytoplasmic fractionation experiments further indicated the predominant location in lysosomes and cytoplasm and a small distribution in the nucleus of exogenous and endogenous FUCA2 protein (Figure 4B–D). Furthermore, the effects of FUCA2 knockdown on protein fucosylation, as indicated by Aleuria aurantia lectin (AAL), and the stability of p53/p27 proteins were effectively blocked by the treatment with tunicamycin, an inhibitor of N‐linked glycosylation (N‐glycosylation) that suppressed the initial step of glycosylation biosynthesis (Figure 4E; Figure S3A). These results thus suggested that the intracellular fucosidase activity was essential for FUCA2 to regulate p53/p27 protein stability in LUAD cells.

It's reasonable to hypothesize that increased fucosidase expression in cancer serves to reinforce the tumor‐promoting effects induced by the downregulated expression of anti‐tumor fucosyltransferases or to counteract unwanted anti‐tumor side effects caused by the upregulated expression of tumor‐promoting fucosyltransferases. Thereby, the expressions of fucosyltransferases, including FUT1‐11, were analyzed using the LUAD datasets from the TCGA database. FUT1 expression was significantly decreased, while the expressions of FUT2, −3, −8 were significantly upregulated in LUAD (Figure S3B). Knockdown of FUT3 significantly attenuated the regulatory effect of FUCA2 knockdown on senescence in A549 and NCI‐H1299 cell lines, whereas knockdown of FUT1, FUT2, or FUT8 had either no effect or only a slight inhibitory impact (Figure 4F,G; Figure S3C–E). Furthermore, FUT3 knockdown diminished the effects of FUCA2 knockdown on p53/p27 protein stability in A549/NCI‐H1299 cells (Figure 4H–K). Together, these results suggested that FUCA2 eliminated the fucosylation of some proteins mediated by FUT3/FUT8, thereby stabilizing p53/p27 proteins and triggering senescence in LUAD cells.

### 2.5 AKT Inactivation Was Essential for FUCA2 Knockdown to Prevent p53/p27 Degradation

**FIGURE 5:**
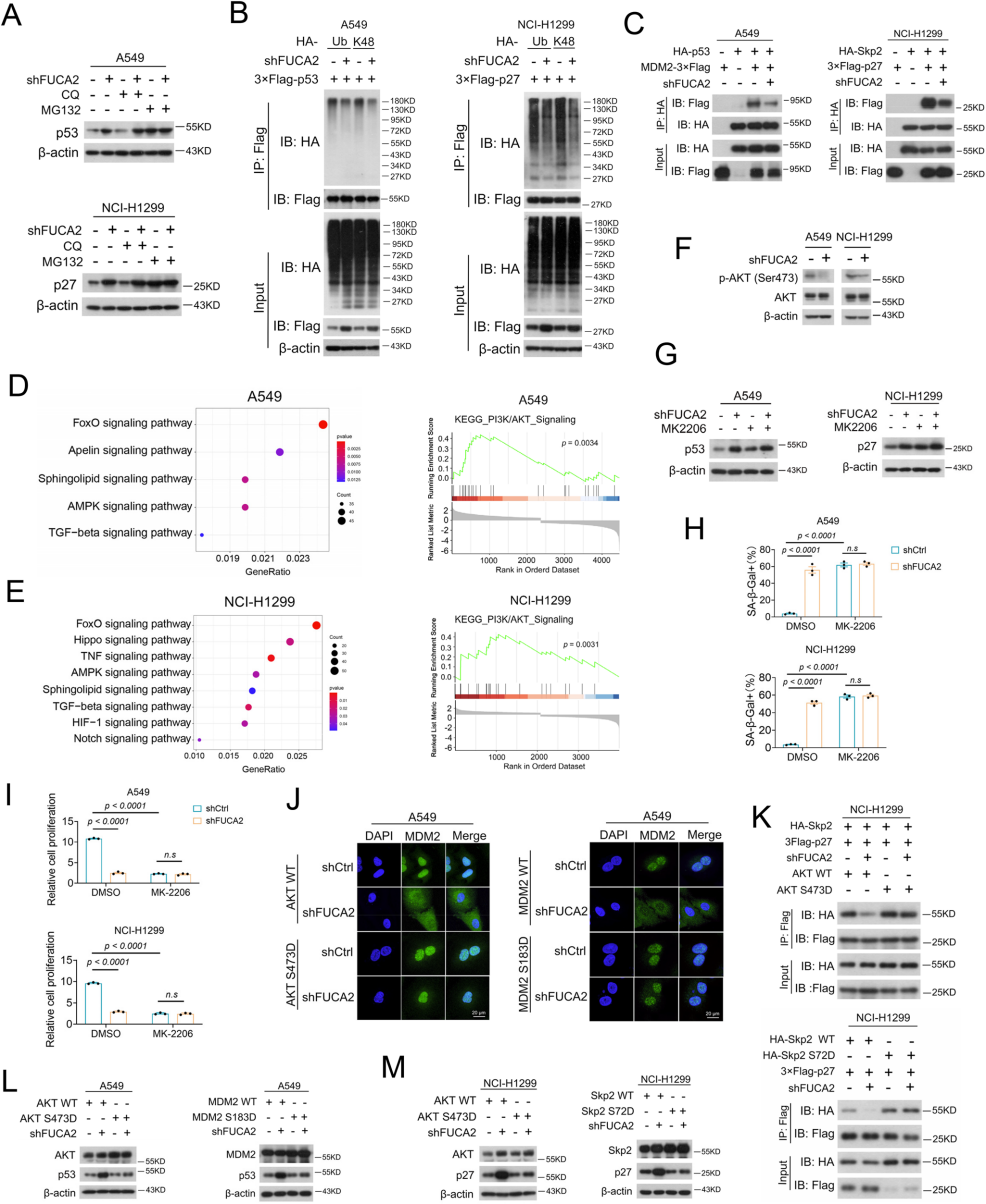
FUCA2 modulated p53/p27 protein levels through the PI3K/AKT signaling pathway. (A) Proteasomal regulation of p53/p27 by FUCA2 was demonstrated in shCtrl/shFUCA2‐transfected A549/NCI‐H1299 cells treated with MG132 (20 µm) or CQ (40 µm) for 24 h. (B) Co‐IP assays revealed FUCA2 knockdown significantly decreased polyubiquitination and K48‐linked ubiquitination of p53 and p27 in A549 and NCI‐H1299 cell lines, respectively. (C) Co‐IP analysis showed reduced p53‐MDM2 interaction in A549 cells and diminished p27‐Skp2 association in NCI‐H1299 cells. (D–F) RNA‐Seq analysis identified the involvement of PI3K/AKT pathway: KEGG enrichment of PI3K/AKT downstream pathways and GSEA plots in A549 (D) and NCI‐H1299 (E) cells, with corresponding p‐AKT (Ser473) levels detected by western blot (F). (G–I) MK‐2206 (20 µm, 3 days) treatment attenuated shFUCA2's tumor‐suppressive effects, as shown by p53/p27 protein levels (G), SA‐β‐gal staining (H), and MTT assays (I). (J) Immunofluorescence in A549 cells overexpressing AKT Wild‐type (WT) or S473D mutant, or MDM2 Wild‐type (WT) or S183D mutant, showed different MDM2 localization upon FUCA2 knockdown (blue: DAPI; green: MDM2; scale bars: 20 µm). (K) NCI‐H1299 cells overexpressing AKT WT/S473D or Skp2 WT/S72D demonstrated different Skp2‐p27 binding status, as detected by co‐IP assay. (L) In A549 cells, AKT S473D (not WT) and MDM2 S183D (not WT) overexpression blocked FUCA2‐knockdown‐induced p53 accumulation. (M) In NCI‐H1299 cells, AKT S473D (not WT) and Skp2 S72D (not WT) overexpression blocked FUCA2‐knockdown‐induced p27 accumulation. Data represented the mean + SEM, and the *p* values were analyzed by one‐way ANOVA (H, I).

To determine whether FUCA2 regulated the degradation of p53/p27 proteins through the ubiquitin‐proteasome pathway or the lysosomal degradation pathway, we treated cells with the proteasome inhibitor MG132 and the autophagy‐lysosome inhibitor Chloroquine (CQ). Effects of FUCA2 knockdown on p53/p27 protein levels could be blocked by MG132 but not by CQ (Figure 5A), indicating that FUCA2 promoted p53/p27 protein degradation via the proteasome pathway. Moreover, the ubiquitination levels of p53/p27 proteins were significantly reduced following FUCA2 knockdown (Figure 5B). Additionally, FUCA2 knockdown markedly inhibited the binding of p53 to its ubiquitin E3 ligase MDM2 and the interaction of p27 with its ubiquitin E3 ligase component SKP2 (Figure 5C). Together, these results suggested that FUCA2 promoted the ubiquitination of p53/p27 and their subsequent proteasome‐mediated degradation by facilitating the binding of p53/p27 to their respective ubiquitin E3 ligases.

Since FUCA2‐promoted degradation of p53/p27 depended on N‐glycosylation (fucosylation), we first analyzed whether p53, p27, MDM2, or SKP2 proteins underwent N‐glycosylation. It has been reported that N‐glycosylation typically occurs in secreted or membrane proteins [29, 30], while p53, MDM2, p27, and SKP2 were not classified as such. Furthermore, treatment with tunicamycin could not reduce the molecular weight of p53, MDM2, p27, and SKP2 proteins in FUCA2 knockdown A549 cells (Figure S4A), indicating that these four proteins were not direct N‐glycosylation targets.

Given that N‐glycosylation‐regulated secreted or membrane proteins often influence cellular signaling, we conducted RNA‐seq to analyze the changes in gene expression profiles before and after FUCA2 knockdown, followed by KEGG pathway analysis. The results showed that FUCA2 knockdown led to the upregulation of 2364 and 2075 genes, and the downregulation of another 2099 and 1921 genes in A549 and NCI‐H1299 cells, respectively (Figure S4B). KEGG pathway enrichment of these genes revealed that following FUCA2 knockdown, there were alterations in the FoxO signaling pathway, AMPK signaling pathway, and sphingolipid signaling pathway, pointing to the PI3K/AKT signaling as a key upstream pathway, which was confirmed by gene set enrichment analysis (GSEA) (Figure 5D,E). Further investigation revealed that FUCA2 knockdown significantly reduced AKT phosphorylation at the Ser473 residue in both A549 and NCI‐H1299 cells, which could be blocked by tunicamycin treatment (Figure 5F; Figure S4C). Furthermore, the AKT inhibitor MK‐2206 blocked the effects of FUCA2 knockdown on p53/p27 protein levels, as well as on cell senescence and proliferation, in both A549 and NCI‐H1299 cells (Figure 5G–I; Figure S4D).

It has been previously reported that AKT phosphorylation of MDM2 at Ser183 enhances nuclear stability of MDM2, leading to decreased p53 levels and prevention of senescence, thereby promoting Kras^G12D^‐driven lung cancers [31]. Additionally, AKT phosphorylation of SKP2 at Ser72 promotes its interaction with p27 and the following ubiquitination and degradation of p27 [32]. When FUCA2 was knocked down in A549 cells, MDM2 translocated from the nucleus to the cytoplasm (Figure S4E). On the other hand, FUCA2 knockdown resulted in a weakened interaction between SKP2 and p27 proteins in NCI‐H1299 cells (Figure 5C). We further found that the effects of FUCA2 knockdown on MDM2 translocation, p27‐SKP2 interaction, p53/p27 protein levels, and cell senescence were blocked by the overexpression of AKT Ser473D, MDM2 S183D, or p27 S72D mutant that mimicked the phosphorylated state, but not wild‐type AKT, MDM2 or p27, in A549 and/or NCI‐H1299 cells (Figure 5J–M; Figure S4F,G). Taken together, these results suggested that FUCA2 promoted AKT activation to phosphorylate MDM2 and SKP2, leading to the nuclear accumulation of MDM2 and enhanced interactions between MDM2 and p53, as well as between SKP2 and p27; this ultimately resulted in the degradation of p53 and p27 proteins, contributing to senescence resistance in A549 and NCI‐H1299 cells.

### 2.6 FUCA2 Hydrolyzed α‐1,3‐Linked Fucosylation of ErbB3 on Asn437 to Activate AKT

**FIGURE 6:**
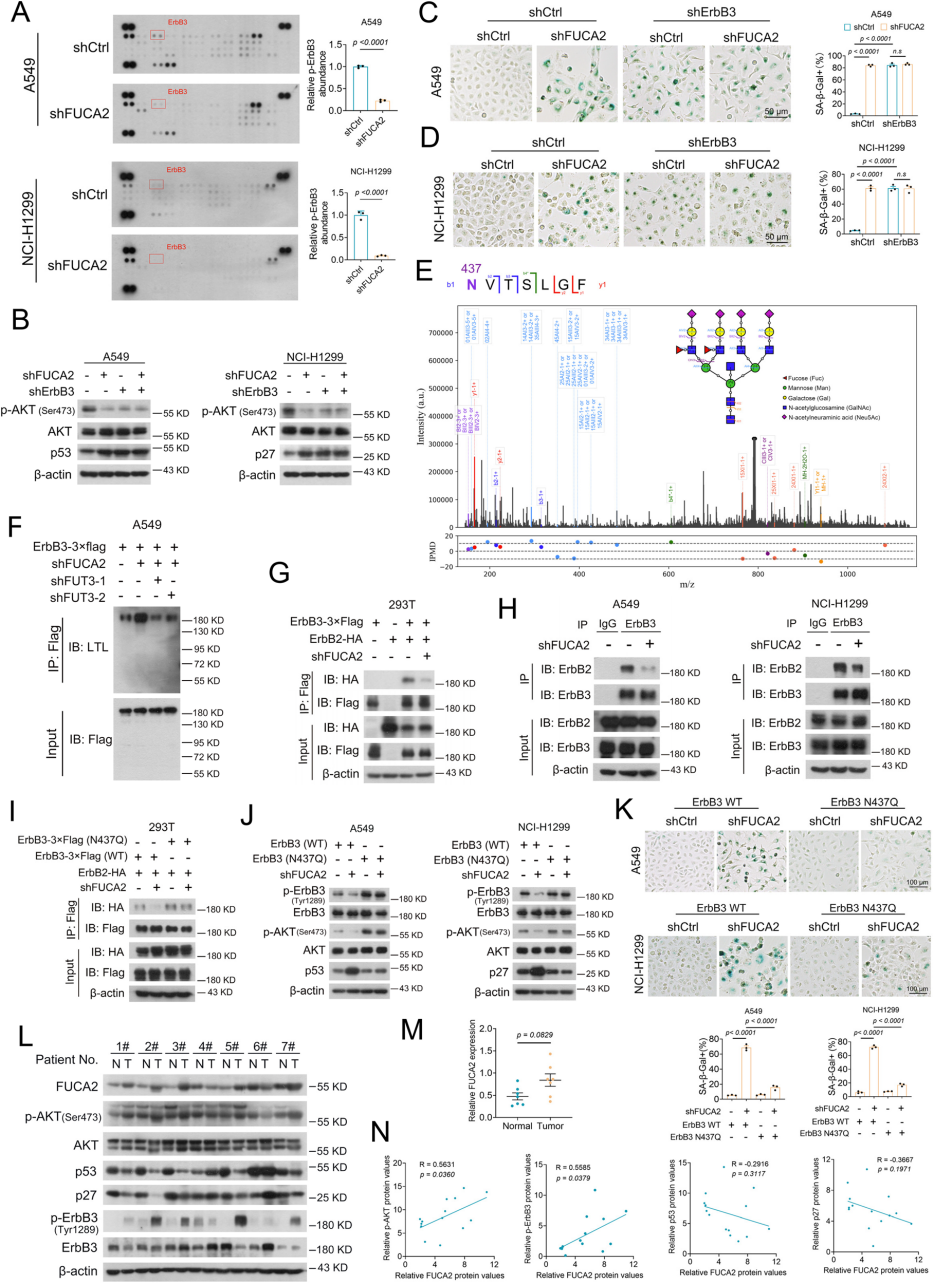
FUCA2 modulated LUAD cell senescence by controlling ErbB3 N437 fucosylation. (A) Human Phospho‐Receptor Tyrosine Kinase Array determined the phosphorylation levels of 59 tyrosine kinase receptors following FUCA2 knockdown in A549 and NCI‐H1299 cells. Quantification of p‐ErbB3 dot intensity was shown, with the shCtrl control group normalized to 1. (B) Western blotting analysis of p‐AKT(Ser473) and p53/p27 levels in FUCA2/ErbB3 co‐knockdown A549 and NCI‐H1299 cells. (C, D) Senescence assessment by SA‐β‐Gal staining in FUCA2/ErbB3 co‐knockdown A549 and NCI‐H1299 cells. (E) UPLC‐MS/MS identified ≥2 fucosylation sites on complex‐type N‐glycans (inset) at ErbB3 Asn437 (N473, purple). (F) LTL lectin binding showed FUT3‐dependent increase in ErbB3 α‐1,3‐fucosylation upon FUCA2 knockdown. (G, H) FUCA2 knockdown impaired ErbB3‐ErbB2 heterodimerization in 293T (G) and A549/NCI‐H1299 (H) cells. (I–K) N437Q mutation of ErbB3 restored the effects of FUCA2 knockdown on ErbB3‐ErbB2 binding (I), levels of p‐ErbB3(Tyr1289), p‐AKT(Ser473), p53 and p27 (J), and cell senescence (SA‐β‐gal staining) (K). (L–N) Levels of FUCA2, p‐AKT, AKT, p53, p27, p‐ErbB3 and ErbB3 in the clinical tissue samples of LUAD were detected by western blotting (L, N: Normal; T: tumor) and quantified by densitometry to analyze the expression level of FUCA2 in normal and tumor tissues (M) and the correlation of FUCA2 with p‐AKT, p‐ErbB3, p53, and p27 (N, analyzed by Pearson r‐test). Data represented the mean + SEM, and the *p* values were analyzed by one‐way ANOVA (C, D, K) or unpaired two‐tailed Student's *t*‐test (M).

We then investigated how FUCA2 regulated AKT phosphorylation. The epidermal growth factor receptor (EGFR) signaling cascade is renowned as the principal upstream pathway for AKT, with EGFR being a membrane‐bound receptor whose N‐glycosylation modulates its membrane translocation, dimerization, phosphorylation, and activation [33]. Contrary to our expectations, FUCA2 knockdown was observed to enhance the expression, membrane location, and phosphorylation of EGFR and the activation of its downstream kinase ERK (Figure S5A,B), a pattern that diverged from the influence of FUCA2 knockdown on AKT activity (Figure 5F), suggesting that FUCA2 did not modulate AKT through the EGFR pathway. To identify potential membrane receptor tyrosine kinases (RTKs) upstream of AKT that were regulated by FUCA2, we employed a human phospho‐RTK array kit encompassing 49 distinct RTKs. Dot‐blot array data revealed a marked decrease in ErbB3 phosphorylation following FUCA2 knockdown in both A549 and NCI‐H1299 cells, a finding corroborated by western blot analysis (Figure 6A; Figure S5C,D). ErbB3, a member of the HER family alongside EGFR, has been documented to stimulate tumorigenesis through the activation of the PI3K/AKT signaling axis [34]. Our further investigations indicated that ErbB3 knockdown in A549 and NCI‐H1299 cells elevated the expression of p53/p27 proteins and triggered cellular senescence (Figure S5E,F). Moreover, ErbB3 knockdown in A549/NCI‐H1299 cells was capable of abrogating the effects of FUCA2 knockdown on AKT phosphorylation, p53/p27 protein levels, and cellular senescence (Figure 6B–D).

Subsequently, our investigations revealed that treatment with PNGase F, an amidase specifically cleaving N‐linked glycan chains from the aspartate residue of glycoproteins, decreased the molecular weight of endogenous ErbB3 in A549 and NCI‐H1299 cells (Figure S5G), confirming the presence of N‐glycosylation modifications in ErbB3 within lung cancer cells. Moreover, we employed biotinylated lectins that recognized different fucosylated sites (AAL, recognizing α‐1,2‐, α‐1,3/4‐, and α‐1,6‐fucosylation; UEA‐1, specifically recognizing α‐1,2‐fucosylation; LTL, specifically recognizing α‐1,3‐fucosylation; LCA, specifically recognizing α‐1,6‐fucosylation) to analyze the type of fucosylated sites in ErbB3 protein isolated via immunoprecipitation from A549 cells. The results showed that α‐1,3‐ and α‐1,6‐fucosylation, but not α‐1,2‐fucosylation, of N‐glycans existed on ErbB3 proteins (Figure S5H). To further investigate the N‐glycosylation sites of ErbB3 and characterize its glycan modifications, ErbB3‐3×Flag were overexpressed and purified from A549 cells, and then subjected to N‐glycosylation mass spectrometry analysis. Seven N‐glycosylation sites were identified across ErbB3's domains I, II, and III, including 59 distinct N‐glycopeptides by UPLC‐MS/MS (Figure S5I,J). Among these, Asn437 exhibited the highest degree of fucosylation and carried a complex‐type glycan with multiple branched structures, two of which featuring α‐1,3‐linked fucose modifications on GlcNAc residues (Figure 6E). Knockdown of FUCA2 increased α‐1,3‐fucosylation of ErbB3 N‐glycans, which was reversed by FUT3 knockdown (Figure 6F). Quantitative mass spectrometry further revealed that FUCA2 knockdown increased α‐1,3‐fucosylation at Asn437 by approximately 2‐fold of the control level, whereas additional FUT3 knockdown on this background rendered it nearly undetectable, confirming that FUCA2 and FUT3 coregulated this site (Figure S5K). To directly validate FUCA2's enzymatic activity toward Asn437, in vitro defucosylation assays were conducted and the results showed that wild‐type FUCA2, but not the catalytically inactive mutant D228A [35], reduced α‐1,3‐fucose levels on ErbB3, an effect absent on the ErbB3(N437Q) mutant (Figure S5L). Functionally, the senescence phenotype induced by FUCA2 knockdown was rescued by a synonymous FUCA2 mutant [FUCA2(r)] but not by FUCA2(r, D228A) (Figure S5M). Finally, to mimic the co‐upregulated state observed in LUAD, we overexpressed FUT3 alone or together with FUCA2. As indicated in Figure S5N,O, FUT3 overexpression alone induced senescence and mildly suppressed cell growth, whereas co‐overexpression of FUCA2 and FUT3 completely abrogated senescence and significantly promoted cell growth, explaining why both enzymes are co‐upregulated in LUAD.

ErbB3 lacks an active kinase domain and requires heterodimerization with other EGFR family members, such as ErbB2, to activate downstream signaling pathways [36]. We discovered that FUCA2 knockdown hindered the interaction between endogenous/exogen ErbB3 and parallel ErbB2, yet had no effect on the interaction between the ErbB3(N437Q) mutant and ErbB2 (Figure 6G–I). To address the mechanistic link, molecular docking revealed that N437 α‐1,3‐fucosylation of ErbB3 disrupted NRG1b binding—a prerequisite for ErbB3 activation and ErbB3‐ErbB2 heterodimerization—thereby impairing dimerization (Figure S5P–T). Consistently, a time‐course analysis showed that phosphorylation levels of ErbB3 and ErbB2 progressively decreased following FUCA2 knockdown, with kinetics slightly lagging behind FUCA2 protein reduction (Figure S5U). Overexpression of ErbB3(N437Q), rather than wild‐type ErbB3, rendered FUCA2 knockdown ineffective in altering ErbB3 phosphorylation, AKT phosphorylation, p53/p27 expression, and cellular senescence in A549 or H1299 cells (Figure 6J,K).

Furthermore, western blot analysis of clinical lung adenocarcinoma samples demonstrated that FUCA2 expression was significantly elevated in lung adenocarcinoma compared to adjacent normal lung tissues, with FUCA2 levels positively correlating with phosphorylation levels of ErbB3 and AKT and negatively with expression of p53/p27 protein (Figure 6L–N; Figure S6A). To expand our clinical validation, we performed multiplex fluorescence staining on a commercial LUAD tissue microarray, comprising 94 LUAD and 86 adjacent normal tissues. The results confirmed that FUCA2 protein levels were significantly elevated in LUAD tissues and positively correlated with p‐AKT (Figure S6B–D). Notably, no significant correlations were observed between FUCA2 expression and tumor stage, grade, or patient survival (Figure S6E,F), although the lack of survival association might be attributed to the limited sample size (93 of 94 LUAD cases were evaluable, as one sample detached during staining). These data indicated that FUCA2 upregulation was an early event in LUAD and persisted across disease stages, supporting its role as a broadly applicable and stage‐independent prognostic biomarker. Consistent with this notion, TCGA analysis revealed that FUCA2‐high tumors were prevalent across LUAD subtypes regardless of TP53/PIK3CA/AKT1/PTEN mutation status (Figure S6G), further underscoring the broad clinical utility of FUCA2.

Together, the above results suggested that FUCA2 preferentially catalyzed the hydrolysis of the α‐1,3‐fucosylation on N437 residue of ErbB3 induced by FUT3, enhancing its interaction with ErbB2 and thereby promoting AKT phosphorylation to induce the degradation of p53 and p27 and prevent cellular senescence in A549 and NCI‐H1299 cells, respectively.

### 2.7 Capivasertib Induced Senescence and Inhibited Growth in FUCA2‐Abundant LUAD Cells Regardless of PIK3CA/AKT1/PTEN/TP53 Genetic Alterations

**FIGURE 7:**
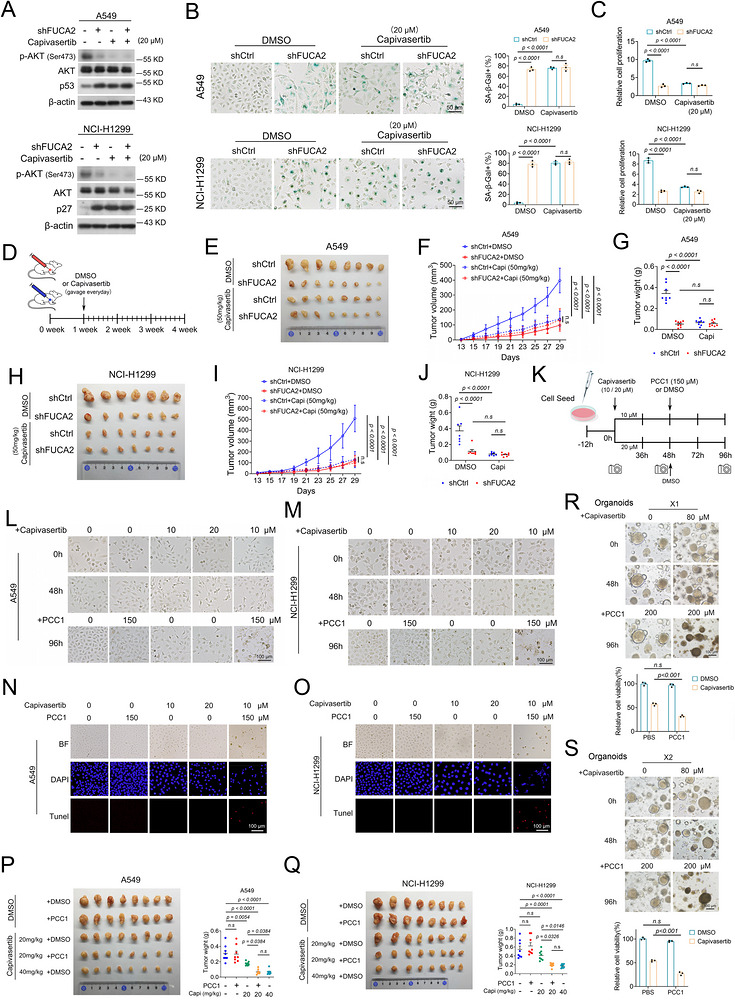
Capivasertib induced cellular senescence and proliferation inhibition in LUAD. (A–C) Treatment with 20 µm Capivasertib suppressed AKT phosphorylation and increased p53/p27 expression (A, by western blotting), promoted cell senescence (B, by β‐galactosidase staining), and inhibited cell growth (C, by cell counting) in an FUCA2‐dependent manner in A549 and NCI‐H1299 cells. (D–J), Capivasertib (50 mg/kg) inhibited the growth of in vivo xenograft derived from A549 and NCI‐H1299 cells in an FUCA2‐dependent manner. Experimental schematic (D), tumor photographs (E, n = 8; H, n = 7), growth curves (F, I), and endpoint tumor weights (G, J) were shown. (K–O) Combination therapy of Capivasertib (10 µm, 48 h pretreatment) with PCC1 (48 h) promoted apoptosis versus Capivasertib alone (20 µm, 96 h): Experimental schematic (K), cellular morphology (L, M), and TUNEL staining (N, O). (P, Q) In vivo validation of sequential treatment (Capivasertib 20 mg/kg/day ×2 weeks → PCC1 20 mg/kg ×2 doses+Capivasertib 20 mg/kg/day ×2 weeks) showing equivalent efficacy to Capivasertib monotherapy (40 mg/kg ×4 weeks) in A549 (P, n = 8) and NCI‐H1299 (Q, n = 8) xenografts. (R, S) Co‐administration of sub‐IC50 Capivasertib (50% IC50) and PCC1 synergistically improved treatment outcomes in LUAD organoids derived from two patients (X1 and X2): spheroid size (upper panel, scale bar: 100 µm) and ATP viability assay (lower panel). Data represented the mean + SEM, and the *p* values were analyzed by one‐way ANOVA (C, D, K, R, S) or unpaired two‐tailed Student's *t*‐test (M).

Next, we explored the potential of targeting over‐activated AKT, due to FUCA2 overexpression, to suppress LUAD progression. Capivasertib, an FDA‐approved AKT inhibitor, is clinically used with Fulvestrant for advanced breast cancer carrying PIK3CA/AKT1/PTEN mutations [37, 38]. We observed that in A549 and NCI‐H1299 cells, which lack PIK3CA/AKT1/PTEN mutations, Capivasertib inhibited cell growth in a dose‐dependent manner (Figure S7A). Notably, Capivasertib primarily induced cellular senescence, rather than apoptosis, at low concentrations (≤ 30 µm), while triggered remarkable apoptosis at higher concentrations (≥ 50 µm) (Figure S7B–D). Treatment with 20 µm Capivasertib significantly suppressed AKT phosphorylation, increased p53/p27 expression and promoted cell senescence, and inhibited cell growth in A549 and NCI‐H1299 cells; importantly, all these capivasertib‐induced effects were abolished upon FUCA2 knockdown (Figure 7A–C). To confirm that Capivasertib's inhibitory effect on LUAD cell growth depended on FUCA2 expression, we evaluated its IC50 value in five LUAD cell lines (all lacking PIK3CA/AKT1/PTEN mutations) with varying FUCA2 levels, alongside the normal lung epithelial cell line BEAS‐2B. The results showed that NCI‐H2122 and NCI‐H1437 cells, exhibiting high FUCA2 expression, displayed pronounced sensitivity to Capivasertib; whereas NCI‐H2126 and BEAS‐2B cells, with low FUCA2, demonstrated resistance; A549 and NCI‐H1299 cells, with intermediate FUCA2 expression, exhibited a moderate response, thereby indicating a strong inverse correlation between Capivasertib's IC50 value and FUCA2 expression (Figure S7E–G). Additionally, knockdown of FUCA2 expression in A549 and NCI‐H1299 cells significantly increased the IC50 value of Capivasertib, while overexpression of FUCA2 in NCI‐H2126 cells reduced the IC50 value (Figure S7H,I). Furthermore, FUCA2 knockdown in treatment‐naive LUAD patient‐derived organoids (lacking PIK3CA/AKT1/PTEN mutations) conferred significant resistance to Capivasertib, as evidenced by elevated IC50 values (Figure S7J). The recommended clinical dose of capivasertib is 400 mg orally twice daily for 4 days on/3 days off in 3–4 week cycles. For a 60 kg patient, this equates to 7.62 mg/kg/day when calculated as a weekly average. The mouse equivalent dose is 69.3 mg/kg/day based on body surface area conversion (9.1× human dose). Thereafter, the inhibitory effect of Capivasertib (50 mg/kg, quaque die, 3 weeks) on the growth of A549 and NCI‐H1299 cells, via inducing widespread senescence and scattered apoptosis, was further validated in vivo using nude mouse xenograft models, which was found to be dependent on FUCA2 expression, with no significant impact on the body weight of the mice (Figure 7D–J; Figure S7K–M). Taken together, our results established FUCA2 as a promising biomarker for predicting Capivasertib response in LUAD, irrespective of PIK3CA/AKT1/PTEN genomic alterations.

The p53 gene ranked as the first most commonly mutated gene in LUAD, with a mutation frequency approaching 50% in the TCGA LUAD cohort (Figure S8A). Among these mutations, approximately 32.4% [(32 frameshift mutations + 35 nonsense mutations)/207 in total] might lead to p53 loss, while around 54.1% (112/207) were missense mutations (Figure S8B). Additionally, among the nine clinical LUAD cases mentioned previously (Figures 2M and 6L), six were wild‐type p53, while the other three carried p53 missense mutations C238Y, V272M, and S172F, respectively (Figure S8C). On this basis, the majority of our experiments focused on cells with either wild‐type p53 (A549) or p53‐deficient (NCI‐H1299) backgrounds. However, given that some mutated p53 has additional functions not seen in wild‐type p53 (i.e., gain of function) [39], it was important to investigate whether the proposed targets, mechanisms, and therapies likewise applied to LUAD cells with p53 missense mutations. In addition to A549 and NCI‐H1299 cells, our study also employed LUAD cell lines NCI‐H1437, NCI‐H2122, and NCI‐H2126, which harbor p53 mutations R267P, C176F, and a deletion, respectively. The sensitivity of these three cell lines to Capivasertib was positively correlated with FUCA2 expression levels (Figure S7E–G). Similarly, the extent of cell senescence induced by FUCA2 knockdown in these cell lines also correlated with their FUCA2 expression (Figure S8D). Upon FUCA2 knockdown, upregulation of p27 protein expression was observed across all three cell lines, with concurrent increases in p53 protein levels noted in NCI‐H1437 and NCI‐H2122 cells (Figure S8E). The C176F, R267P, C238Y, V272M, or S172F mutation of p53 is classified as a loss‐of‐function mutation, while several other hotspot mutations in LUAD (such as R158L, R273L, and G245V), are considered gain‐of‐function mutations [39] (Figure S8B,F). However, the role of these mutated p53 protein in cell senescence remains unclear. To address this, we stably overexpressed wild‐type p53, C176F, R158L, R273L, or G245V mutant of p53 in p53‐negative NCI‐H1299 cells, and assessed their responses to Capivasertib. Overexpression of wild‐type p53 or p53 mutant did not alter Capivasertib sensitivity of NCI‐H1299 cells (Figure S8G). Coincidentally, FUCA2 knockdown‐induced cell senescence rate was not influenced by the overexpression of wild‐type p53 or p53 mutant (Figure S8H). Upregulated expression of p27 protein, as well as exogenous p53 protein, was detected under FUCA2 knockdown in NCI‐H1299 cells (Figure S8I). Moreover, p27 knockdown blocked Capivasertib‐stimulated cell senescence in NCI‐H1299 cells with exogenous mutated p53, but not in those cells with exogenous wild‐type p53 (Figure S8J), indicating that Capivasertib utilized p27 pathway to induce senescence in cells with mutated p53 (loss‐of‐function or gain of function), similar to p53‐deficient cells. Therefore, therapeutic targeting of the FUCA2‐AKT axis by Capivasertib, which induced senescence‐mediated growth arrest in LUAD, emerged as a promising treatment strategy independent of TP53 genetic background.

### 2.8 Low‐Dose Capivasertib Primed LUAD for Senescence‐Targeting by Procyanidin C1

Capivasertib, when used in clinical applications, may cause a range of side effects, including but not limited to nausea, vomiting, fatigue, loss of appetite, and rash [40]. We therefore explored reducing its dosage alongside other well‐tolerated therapeutics to mitigate toxicities and side effects while preserving anticancer efficacy. In recent years, “Senolytic Therapy,” a cutting‐edge approach that selectively targets and eradicates senescent cells, has attracted considerable attention as a promising novel strategy in cancer treatment [41]. Procyanidin C1 (PCC1), a nutraceutical extracted from grape seeds, demonstrated a remarkable ability to efficiently and safely eliminate senescent cells in aging‐related normal cells, including stromal cells, retinal cells, fibroblasts, and immunocytes [42, 43, 44, 45]. However, whether it can also clear senescent cancer cells remains unclear. Hence, we investigated the potential of combining low‐dose Capivasertib with PCC1 as a senolytic strategy to inhibit LUAD. To determine the effective concentration of PCC1, A549 and NCI‐H1299 cells were first treated with 20 µm Capivasertib for 2 days, followed by PCC1 treatment at different concentrations (50, 100, and 150 µm selected based on previously reported dosages for senolysis of senescent stromal cells [42]) for another 2 days (Figure S9A). Results showed that 100 and 150 µm, but not 50 µm, PCC1 effectively induced cell death in senescent LUAD cells (Figure S9B,C). Mechanistic senolysis assays were then performed to confirm that PCC1 eliminated senescent LUAD cells by inducing apoptosis, as evidenced by increased Annexin V positivity, mitochondrial ROS accumulation, and caspase activation (Figure S9D–F). Formal synergy analysis using the Chou‐Talalay method (CompuSyn) further confirmed that the combination of Capivasertib and PCC1 manifested synergistic cytotoxicity, with a Combination Index (CI) < 1 across multiple Fa levels in both A549 and NCI‐H1299 cells (Figure S9G,H). Compared to 100 µm, 150 µm PCC1 exhibited stronger and more potent senolytic activity, achieving approximately 80% growth inhibition (Figure S9A–C), and was therefore selected for subsequent experiments. We further reduced the Capivasertib concentration and found that sequential treatment (10 µm Capivasertib for 2 days, then 150 µm PCC1 for 2 days) induced senescence and apoptosis, yielding a similarly high 80% growth inhibition (Figure 7K–O). We further verified this “one‐two punch” therapeutic strategy for LUAD, which combined senescence‐inducing therapy (low‐dose Capivasertib) and the interinserted senolytic therapy (PCC1), in various mouse tumor models. To justify the selection of 20 mg/kg as the low dose for Capivasertib, we performed an in vivo dose‐response study at 5, 10, 20, and 40 mg/kg, which revealed a clear concentration‐dependent trend. The 20 mg/kg dose was selected as the minimal effective dose with consistent senescence induction and good tolerability (Figure S9I,J). Treatment with low‐dose Capivasertib (20 mg/kg, quaque die, 4 weeks) plus PCC1 (20 mg/kg, twice) manifested a comparable anti‐LUAD effect to higher‐dose Capivasertib (40 mg/kg, quaque die, 4 weeks) (Figure 7P,Q; Figure S9K–M). Importantly, this synergistic tumor‐suppressive effect was consistently recapitulated in patient‐derived LUAD organoids from two independent cases (Figure 7R,S, organoids were more resistant than cells, requiring modestly increased drug concentrations), further validating the translational potential of this senolytic therapy.

## 3 Discussion

The regulation of protein fucosylation in cancer presents intriguing paradoxes. While fucosyltransferases including FUT8 and FUT3 are frequently upregulated in LUAD and promote oncogenesis by modifying key signaling molecules (e.g., TGFBR1, EGFR, and immune checkpoints) [18, 19, 20], our findings reveal that the same modification can exert tumor‐suppressive effects when targeting specific substrates such as ErbB3. This duality suggests that tumors may upregulate fucosidases like FUCA2 as a counterbalance to mitigate the antitumor consequences of excessive α‐1,3‐fucosylation on certain proteins. Indeed, elevated FUCA2 expression in LUAD acts as a safeguard mechanism, selectively removing inhibitory fucose moieties from ErbB3 to reactivate AKT and bypass senescence. Although FUCA2 upregulation showed inter‐individual heterogeneity (elevated in five of seven paired tissues, slightly lower or comparable in the other two; Figure 6L), likely due to differences in genetic background or tumor microenvironment, the overall trend remained significant. Notably, the negative correlation of FUCA2 with p53 and its positive correlation with p‐ErbB3/p‐AKT were consistently observed across all seven pairs, reinforcing the robustness of this axis. The opposing roles of FUT3 and FUCA2 on ErbB3 illustrate how tumors exploit fucosylation dynamics to fine‐tune signaling, potentially explaining their frequent co‐upregulation. These findings position FUCA2 as both a critical rheostat in LUAD progression and a therapeutic vulnerability—its inhibition forces ErbB3 hyperfucosylation, triggering irreversible senescence regardless of TP53 status. Deciphering such context‐dependent crosstalk is essential for developing precision strategies to manipulate fucosylation in resistant tumors.

Despite belonging to the same α‐L‐fucosidase family, FUCA1 and FUCA2 exhibit divergent expression patterns and prognostic significance in LUAD, suggesting distinct functional roles in tumor biology. Moreover, FUCA1 knockdown did not suppress ErbB3 or AKT phosphorylation nor induce cellular senescence in LUAD cells (Figure S10A–C), indicating that FUCA1 did not share FUCA2's substrate specificity toward ErbB3 and that no functional compensation occurred between the two fucosidases. Our findings, combined with existing literature, reveal that these differences likely originate from their substrate and linkage specificities. The linkage‐dependent divergence is exemplified by EGFR regulation: FUT4/6‐mediated α‐1,3‐fucosylation inhibits EGFR dimerization, whereas FUT8‐driven α‐1,6‐fucosylation promotes it; FUCA1's α‐1,6‐hydrolysis bias results in inhibition of EGFR and its downstream signaling, such as Akt phosphorylation [7, 46, 47]. In contrast, our work demonstrates FUCA2's pro‐tumorigenic role in cleaving FUT3‐dependent α‐1,3‐fucosylation on ErbB3 to sustain AKT signaling. These observations raise critical therapeutic questions—whether to selectively target FUCA2's active site to preserve FUCA1's tumor‐suppressive functions or to develop downstream inhibitors (e.g., AKT inhibitors) to circumvent off‐target effects. Resolving these questions will require structural studies (e.g., crystallography) coupled with systematic profiling of FUCA1/2 substrates in LUAD, ultimately guiding precision strategies to exploit fucosylation dynamics in cancer therapy.

Capivasertib, the first AKT inhibitor approved by the FDA on November 16, 2023, is indicated for use with Fulvestrant (Estrogen receptor antagonist) in treating hormone receptor‐positive, HER2/ERBB2‐negative, locally advanced or metastatic breast cancer with one or more PIK3CA/AKT1/PTEN mutations [37, 38]. However, the potential utility of Capivasertib in tumors lacking PIK3CA/AKT1/PTEN mutations remains unclear. Moreover, while clinical use of Capivasertib is associated with adverse effects such as nausea, vomiting, fatigue, decreased appetite, and rash, dose reduction significantly diminishes its efficacy in promoting apoptosis and suppressing tumor growth [40]. Here, we found that administration of a minuscule dose of Capivasertib induced senescence in FUCA2‐abundant LUAD cells without PIK3CA/AKT1/PTEN mutations, regardless of TP53 gene status (mutated or not). Importantly, combined treatment with low‐dose Capivasertib and the flavonoid PCC1 (a nutraceutical), as senolytics, therapeutically restrained LUAD growth in vitro and in vivo (employing five human LUAD cell lines, mouse models, and organoids derived from LUAD patients). Therefore, this FUCA2‐guided strategy, which employs low‐dose Capivasertib (beyond its current genomic indication) together with adjuvant procyanidin C1, circumvents the challenges of *de novo* FUCA2 inhibitor development and offers a promising path for rapid clinical translation.

Cellular senescence represents a state of irreversible cell cycle arrest that has emerged as a promising therapeutic strategy in oncology [24]. The fate of senescent cells is context‐dependent: while some develop a persistent senescence‐associated secretory phenotype (SASP) that may paradoxically promote tumor progression (necessitating senolytic agents for clearance), others undergo spontaneous apoptosis, exerting tumor‐suppressive effects [48, 49]. Here, our findings demonstrate that FUCA2 knockdown triggers a sequential cascade in LUAD cells: (1) stabilization of p53/p27 proteins; (2) cell cycle arrest and a decline in proliferation; (3) senescence; (4) some of the senescent cells ultimately undergo apoptosis. The transition from senescence to apoptosis appears driven by progressive accumulation of p53/p27, with senescence serving as the critical intermediate stage. As the levels of p53/p27 proteins continue to accumulate, the balance of senescence is disrupted, triggering apoptosis. We observed a parallel phenomenon with AKT inhibitors: low concentrations predominantly induced senescence, while higher concentrations accelerated p53/p27 accumulation, leading to apoptosis. Notably, this pathway accommodates LUAD heterogeneity: p53‐wildtype cells utilize p53 stabilization, whereas p53‐deficient/mutant cells employ p27 (rarely mutated in LUAD), ensuring broad applicability.

Collectively, these findings establish the FUCA2–ErbB3 fucosylation–AKT axis as a key regulator of cellular senescence and put forward a FUCA2‐guided drug repurposing strategy—combining low‐dose capivasertib, applied beyond its current genomic indication, with the nutraceutical adjuvant procyanidin C1—as a promising therapeutic avenue for LUAD, independent of TP53/PIK3CA/AKT1/PTEN genetic alterations.

## 4 Methods

### 4.1 Bioinformatics Analysis

Details of the methodology for the identification of potential therapeutic targets for LUAD were described in the previous study [50].

For FUCA2 expression landscape across cell types in NSCLC, we utilized the TISCH2 Database (http://tisch.comp‐genomics.org/) to analyze NSCLC datasets.

### 4.2 Cell Culture

The lung adenocarcinoma cell lines A549, NCI‐H1299, NCI‐H1437, NCI‐H2122, and NCI‐H2126, along with the human embryonic kidney cell line HEK‐293T, were obtained from the Shanghai Cell Bank. The normal lung epithelial cell line BEAS‐2B was generously provided by Professor Zhenghong Zuo's laboratory (School of Medicine, Xiamen University). All cells were cultured in a humidified incubator at 37°C with 5% CO_2_. The A549, NCI‐H1299, NCI‐H1437, NCI‐H2122, and NCI‐H2126 cell lines were maintained in RPMI‐1640 medium (Gibco, #31800022) supplemented with 10% fetal bovine serum (Gibco, #10270106). HEK‐293T cells were cultured in DMEM medium (Gibco, #12800082) containing 10% fetal bovine serum, while BEAS‐2B cells were grown in Complete BEAS‐2B Cell Medium with Serum and Supplements (Beyotime, C6106C). All cell lines were authenticated by STR profiling and used within two months of thawing.

### 4.3 Antibodies and Reagents

All antibodies and reagents used in this study are provided in Tables S1 and S2.

### 4.4 Cell Proliferation Assays

For the MTT assay, cells were seeded in 96‐well plates (4 × 10^3^ cells/well, n = 5 replicates) and incubated with MTT reagent (0.5 mg/mL, Sigma–Aldrich #M2128) for 4 h at 37°C. The resulting formazan crystals were dissolved in DMSO (BBI Life Sciences #A610163), and absorbance was measured at 570 nm with a 630 nm reference wavelength using a TECAN Infinite F50 microplate reader.

For the colony formation assay, cells were plated in 6‐well plates (1 × 10^3^ cells/well, n = 3 replicates) and cultured for 7 days. Colonies were fixed with 4% methanol for 10 min, stained with 0.01% (w/v) crystal violet for 10 min, then air‐dried before imaging and quantification.

### 4.5 Annexin V/PI Apoptosis Assay

Cell apoptosis was quantified using the FITC‐Annexin V/PI Apoptosis Detection Kit (YEASEN, #40302ES50) following the manufacturer's protocol. Flow cytometry analysis was performed on a Beckman Coulter CytoFlex instrument. Apoptotic cells were identified as Annexin V‐positive populations, and data were analyzed using FlowJo software (version 10.6.2).

### 4.6 Western Blotting

Cellular proteins were extracted using ice‐cold ELB lysis buffer (50 mM Tris‐HCl [pH 7.6], 140 mm NaCl, 100 mm NaF, 0.5% NP‐40, 2 mm NaVO3, 5 mm β‐glycerol phosphate, and 1 mm PMSF) followed by centrifugation (13 200 × *g*, 30 min, 4°C). Protein concentrations in the cleared lysates were quantified using a BCA protein assay (Bio‐Rad). Equal amounts of protein were denatured in 2× Laemmli buffer (100°C, 15 min), resolved by SDS‐PAGE, and electrotransferred onto PVDF membranes. Membranes were blocked with 5% non‐fat milk in TBS‐T (20 mm Tris‐HCl [pH 7.5], 100 mm NaCl, 0.1% Tween‐20) for 1 h at room temperature before overnight incubation with primary antibodies (4°C). After TBS‐T washes, membranes were probed with HRP‐conjugated secondary antibodies (3 h, room temperature) and developed using enhanced chemiluminescence reagents (Millipore).

### 4.7 RNA Extraction and Real‐Time PCR

Total RNA was isolated using TRIzol reagent (Takara, #9109) and reverse‐transcribed into cDNA using the PrimeScript RT reagent kit (Takara, #RR036A). Quantitative real‐time PCR was performed on a Roche LightCycler 96 Real‐Time PCR System with TB Green Premix Ex Taq II (Takara, #RR820). All primer sequences are provided in Table S3.

### 4.8 Lentivirus‐Mediated Gene Knockdown or Overexpression

Cells at 70%–80% confluence were transfected using PEI4000 transfection reagent (YEASEN, #40816ES02). Viral supernatant was harvested after 48 h, centrifuged, and filtered through a 0.45 µm PVDF membrane (Millipore, #HAWP04700). Lentiviral particles were concentrated by ultracentrifugation (Thermo Scientific Sorvall ST 16R) and titers were determined prior to immediate use or storage at −80°C. For transduction, target cells were plated at 30%–40% confluence and incubated with concentrated lentivirus. Following 48 h incubation, media were replaced with selection medium containing appropriate antibiotics. All shRNA sequences are provided in Table S4.

### 4.9 Protein Stability Analysis

Cells in logarithmic growth phase were plated in 35 mm culture dishes at 70% confluence. Following cell attachment, fresh medium containing cycloheximide (Cell Signaling Technology, #2112; 50 µg/mL for A549, 80 µg/mL for NCI‐H1299) was applied. Whole‐cell lysates were harvested at specified time intervals and subjected to immunoblotting to determine protein degradation kinetics.

### 4.10 Protein Ubiquitination Analysis

Log‐phase cells were cultured in 10 cm dishes until reaching 80% confluence. Following 12‐h incubation, cells were transfected with either control or knockdown plasmids, along with pLVX‐3×Flag‐p53/3×Flag‐p27 and pCDNA3.3‐HA‐Ub constructs. Flag‐tagged proteins were immunoprecipitated using anti‐Flag nanobody‐conjugated magnetic beads (Shenzhen KT Life Technology, #KTSM1360). Ubiquitination levels were subsequently analyzed by immunoblotting with an anti‐HA antibody (Invitrogen, #26183).

### 4.11 Co‐Immunoprecipitation (Co‐IP)

Transfected cells were lysed in ice‐cold lysis buffer (50 mm Tris‐HCl, 150 mm NaCl, 1 mm EDTA, 1% NP‐40, 5% glycerol) using sonication. Cleared lysates were obtained by centrifugation (13 000 × *g*, 4°C) and incubated with anti‐Flag M2 affinity gel (Beyotime, #P2055) for 12 h at 4°C with rotation. The immunocomplexes were washed three times with lysis buffer, eluted in 5× SDS loading buffer by boiling (95°C, 5 min), and resolved by SDS‐PAGE followed by immunoblotting.

### 4.12 Senescence‐Associated β‐Galactosidase Staining

Cells were plated in 12‐well plates at 30%–40% confluence and cultured to 80%–90% density. Following fixation (15 min) and four 5‐min washes with 1× PBS, senescence‐associated β‐galactosidase activity was detected using a commercial staining kit (Beyotime, #C0602) according to the manufacturer's protocol (37°C, 12–16 h). Bright‐field images were acquired at 40× magnification (Olympus IX73 microscope). SA‐β‐Gal‐positive cells were quantified by counting 100 cells per field across three independent fields per condition, with results presented as mean percentage positivity ± standard error of the mean (SEM).

### 4.13 Protein Phosphorylation Analysis

Cells were harvested and lysed in RIPA buffer (Beyotime, #P0013C) supplemented with phosphatase inhibitor cocktail (Roche, #4906837001). Lysates were sonicated on ice and centrifuged (13 000 × g, 4°C). Supernatants were combined with 5× SDS loading buffer, denatured at 95°C for 5 min, and separated by SDS‐PAGE. Proteins were transferred to PVDF membranes, and phosphorylation of target proteins was assessed using phospho‐specific antibodies.

### 4.14 Immunofluorescence Analysis

Cells were plated on sterile glass coverslips in 12‐well plates and allowed to adhere for 12 h (30%–40% confluence). After fixation with 4% paraformaldehyde (10 min, RT) and three washes with 1× PBS, cells were permeabilized with 0.2% Triton X‐100 (10 min) followed by additional PBS washes. Non‐specific binding sites were blocked with 5% BSA (1 h, room temperature). Primary and corresponding Alexa Fluor‐conjugated secondary antibodies (Invitrogen; 488 nm/594 nm) were applied sequentially. Nuclei were counterstained with DAPI‐containing mounting medium (Solarbio, #S2110) prior to imaging on a ZEISS LSM 880 confocal microscope with Airyscan super‐resolution capability.

### 4.15 Lectin Blot Analysis

#### 4.15.1 Whole‐Cell Protein Analysis

Total lysates were separated by SDS‐PAGE and transferred to PVDF membranes. After blocking with 5% BSA, membranes were probed with biotinylated lectins (overnight, 4°C), washed with TBST, and incubated with streptavidin‐HRP (Beyotime, #A0303; 1 h, room temperature) prior to chemiluminescent detection. Note: Protein samples should be analyzed immediately without freezing to preserve glycosylation patterns.

#### 4.15.2 Target‐Specific Analysis

Cells transfected with expression plasmids for 48 h were lysed, and target proteins were immunoprecipitated using anti‐Flag magnetic beads. Enriched proteins were resolved by SDS‐PAGE, followed by lectin blotting as above.

### 4.16 TUNEL Apoptosis Detection

Treated cells in 24‐well plates were washed with 1× PBS and fixed with 4% paraformaldehyde (20–30 min, room temperature). After permeabilization with 0.3% Triton X‐100/PBS (5 min) and PBS washes, cells were incubated with TUNEL reaction mixture (Beyotime, #C1089; 100 µL/well, 37°C, 60 min, dark). Following two PBS washes, samples were mounted using anti‐fade medium for fluorescence microscopy.

### 4.17 Phospho‐Receptor Tyrosine Kinase (RTK) Profiling

Cells were processed per the manufacturer's protocol (R&D Systems, #ARY001B). Briefly, PBS‐washed cells (1 × 10^7^ cells/mL) were lysed in ice‐cold Lysis Buffer containing protease inhibitors. Lysates were gently inverted in 1.5 mL microcentrifuge tubes and rotated (4°C, 30 min). After centrifugation (14 000 × g, 5 min, 4°C), cleared supernatants were diluted and incubated with nitrocellulose membranes (4°C, overnight). Membranes were washed (3 × 10 min with Wash Buffer), probed with anti‐phospho‐tyrosine‐HRP antibody (room temperature, 2 h, dark), washed again, and developed using chemiluminescence.

### 4.18 In Vitro Defucosylation Assay

293T cells were transfected with 20 µg of plasmids (pLVX‐FUCA2‐WT/D228A‐3Flag, pCAGGS‐ErbB3‐WT/N437Q‐3Flag) using PEI (1:3 DNA:PEI ratio). ErbB3‐expressing groups were co‐transfected with pLVX‐FUT3‐3HA to enhance fucosylation. After 48 h, cells were lysed using an Anti‐Flag Nanobody IP Kit (AlpalifeBio, #KTSM1360), and Flag‐tagged proteins were purified by anti‐Flag beads and eluted with 150 µg/mL 3× Flag Peptide (Beyotime, #P9801). The defucosylation reaction (50 µL total) contained 20 µL ErbB3 substrate, 10 µL FUCA2 enzyme, and 50 mm citrate‐disodium phosphate buffer (pH 5.0). After incubation at 37°C for 30 min, the reaction was stopped with 5× loading buffer, boiled for 10 min, and analyzed by Western blotting and LTL blot analysis.

### 4.19 Mitochondrial ROS Detection

NSCLC cells were treated with Capivasertib for 48 h followed by PCC1 for 48 h. Cells were then stained with 5 µm MitoSOX Red (Beyotime, #S0061S) at 37°C for 30 min and analyzed by flow cytometry (Ex/Em: 405/610 nm).

### 4.20 Combination Therapy and CI Calculation

Cells were seeded in 96‐well plates (5000 cells/well), pretreated with Capivasertib for 48 h, and then co‐treated with PCC1 (Capivasertib:PCC1 = 1:15) for another 48 h. Cell viability was measured by MTT assay, and CI values were calculated using CompuSyn software (ComboSyn Inc., Paramus, NJ, USA). CI < 1, = 1, or > 1 indicates synergy, additivity, or antagonism, respectively.

### 4.21 Structural Modeling and Molecular Docking

To thoroughly sample the stable conformational space of glycosylated ErbB3, extensive all‐atom MD simulations (100 ns) were executed employing the Desmond package (Schrödinger). The complex was placed in an explicit water box with counter ions, energy‐minimized, and gradually heated to 300 K under NPT conditions. Trajectory frames were saved every 100 ps, and representative conformations were extracted by cluster analysis. The glycosylated ErbB3 was treated as an integral receptor for docking.

For docking, the unmodified ErbB3‐NRG1b complex (PDB: 7MN8) was used as a reference. Redocking experiments validated the HADDOCK3 workflow [51]. The glycosylated ErbB3 receptor was then docked with NRG1b using HADDOCK3 following a multi‐step protocol: topology building, rigid‐body sampling with distance restraints, semi‐flexible refinement, and final energy minimization in explicit water. Docking poses were clustered and scored using HADDOCK3. The top‐scoring cluster was superimposed onto the 7MN8 structure to assess the steric effects of glycosylation on ErbB3‐NRG1b binding.

### 4.22 Clinical Tissue Analysis

This study was conducted in accordance with the Declaration of Helsinki and approved by the Medical Ethics Committees of Xiang'an Hospital Affiliated to Xiamen University (Ethical Number: XAHLL2025029), Fujian Provincial Hospital (Ethical Number: K2022‐05‐005), and the Internal Ethical Review Committee of Shanghai Outdo Biotechnology (Ethical Number: SHYJS‐CP‐2304002). Informed consent was obtained from all participating patients prior to sample collection.

For organoid‐related studies, lung tissue specimens (designated X1# and X2#) were procured from Xiang'an Hospital Affiliated to Xiamen University.

For western blot analysis, additional clinical samples (No. 1#–7#) were obtained from the tissue repository at Fujian Provincial Hospital. Following collection, samples were immediately homogenized in ice‐cold lysis buffer using standardized protocols and subsequently analyzed by immunoblotting.

For multiplex fluorescence staining, a commercial LUAD tissue microarray (Shanghai Outdo Biotech Co., Ltd., #HLugA180Su09, Slice Number: M060) was used, and the staining results were scored for statistical analysis. Each microarray contained a total of 180 paraffin‐embedded lung tissue samples, including 94 cases of LUAD tissues and 86 cases of adjacent normal lung tissues.

### 4.23 Organoid Culture and Assay Protocols

#### 4.23.1 Generation of Organoids

Fresh tumor tissue samples from two lung adenocarcinoma patients (named X1 and X2 from Xiang'an Hospital of Xiamen University) were immediately preserved in tumor tissue storage solution (MB‐0818L04S), with organoid establishment completed within 24 h. Briefly, 1 cm^3^ tissue fragments were washed twice in D‐PBS containing double antibiotics and mechanically dissociated into 1–3 mm^3^ pieces. The fragments were enzymatically digested using tumor tissue digestion solution (MB‐0818L05S) at 37°C for 20–30 min with gentle agitation every 5 min. The reaction was neutralized with serum‐supplemented basal medium (MB‐0818L07), and the suspension was filtered through a 100 µm cell strainer. After centrifugation (300 × *g*, 5 min), red blood cell lysis was performed using MB‐0818L08S (1–3 min). The cell pellet was washed twice with basal medium before resuspension in ice‐cold Matrigel (#082703) and plating in low‐adhesion plates (48‐well: 25 µL/well; 24‐well: 50 µL/well). Following Matrigel polymerization (37°C, 15–30 min), lung adenocarcinoma‐specific organoid culture medium (MA‐0807T002LP) was added and refreshed every 2–3 days.

#### 4.23.2 Organoid Digestion and Passaging

For subculture, the conditioned medium was retained and supplemented with 500 µL fresh basal medium. The Matrigel‐organoid matrix was gently dislodged and transferred to a microcentrifuge tube. After centrifugation, the pellet was digested with 5–10 volumes of organoid digestion solution at 37°C for 5–10 min until appropriately fragmented. The reaction was neutralized with an equal volume of basal medium, followed by centrifugation (300 × *g*, 3 min) and one wash. The organoid fragments were re‐embedded in fresh Matrigel, polymerized (37°C, 15–30 min), and cultured in complete medium for expansion or subsequent applications.

#### 4.23.3 Organoid Viability Assay

Organoid cultures were equilibrated to room temperature for 10 min before adding pre‐warmed detection reagent (bioGenous Organoid Viability ATP Assay Kit, E238003) at a 1:1 ratio with culture medium. Complete lysis was achieved by vigorous shaking (5 min), followed by 20‐min incubation at room temperature. Chemiluminescence was measured at 560 nm using a microplate reader.

#### 4.23.4 Infection of Organoids With Lentivirus

Organoids were transduced on Matrigel‐coated 48‐well plates using lentiviral particles (1:1 v/v) in the presence of Polybrene (10 µg/mL, Beyotime, C0351) for 48 h.

#### 4.23.5 β‐Galactosidase Staining of Organoids

Mature organoids were recovered using organoid recovery solution (MA‐0837DS001P, 4°C, 30 min) and transferred with low‐adhesion pipette tips (Corning, 4714) to specialized tubes (Eppendorf, 30108116). After centrifugation (100 g, 3 min) and one wash, organoids were fixed (37°C, 2 h), washed three times with PBS, and stained at room temperature for 10 h before imaging.

### 4.24 Multiplex Fluorescence Staining

The clinical LUAD tissue microarray was baked at 65°C for 2 h or 60°C overnight, then dewaxed in eco‐friendly dewaxing solution (15 min, twice), rehydrated sequentially in absolute ethanol (5 min, twice), 90% ethanol (5 min, once), 75% ethanol (5 min, once), and distilled water (5 min, once). For antigen retrieval, slides were placed in retrieval buffer, microwaved until boiling (3 min on high power), then incubated at low temperature for 15–20 min, rapidly cooled with ice, washed with TBST (5 min, 2–3 times), and blocked with blocking buffer for 30 min to 2 h at room temperature (RT). Slides were then incubated with primary antibody (RT for 1–2 h or 4°C overnight), washed with TBST (5 min, 2–3 times), incubated with secondary antibody for 10 min at RT, washed again, followed by fluorescent dye (1:100) for 10–15 min at RT and TBST washing. These steps (antigen retrieval to dye labeling) were repeated for each additional marker. Finally, slides were incubated with DAPI for 10 min, washed with TBST, the hydrophobic barrier removed, and mounted with mounting medium.

### 4.25 Subcutaneous Xenograft Model

All animal studies were conducted in accordance with institutional ethical guidelines and were approved by the Institutional Animal Care and Use Committee of Xiamen University.

Stable knockdown subclones of A549 and NCI‐H1299 cells were generated via lentiviral transduction and cultured under standard conditions (37°C, 5% CO_2_). During logarithmic growth phase, cells were harvested by trypsinization and resuspended in serum‐free medium (5 × 10^6^ cells/200 µL). Cell suspensions were implanted subcutaneously in the vascularized right axillary fossa of 4–6‐week‐old female Balb/c nude mice. Tumor progression was monitored through biweekly caliper measurements, with volumes calculated as (length × width^2^)/2. Upon reaching the ethical endpoint (15 mm maximal diameter), mice were euthanized for tumor resection and morphological analysis.

### 4.26 Immunohistochemical (IHC) Staining

Tissue sections (5 µm) from paraffin‐embedded xenografts underwent standard deparaffinization and rehydration. Antigen retrieval was performed via microwave irradiation in citrate buffer (pH 6.0). After blocking with 5% bovine serum albumin (BSA), sections were incubated with primary antibodies (4°C, overnight) followed by biotinylated secondary antibodies (room temperature, 1 h). Immunoreactivity was detected using the streptavidin‐peroxidase (SP) system with 3,3'‐diaminobenzidine (DAB) chromogenic development, followed by hematoxylin counterstaining (Sigma, #HHS32) for nuclear visualization.

### 4.27 Statistical Analysis and Reproducibility

Data were derived from three independent biological experiments with triplicate technical replicates per condition unless otherwise noted. Normality was assumed without formal testing. Sample sizes were chosen following standard parameters employed in analogous experimental paradigms. No data were excluded from analyses. Randomization was applied only for treatment group allocation in animal studies, with no blinding during data collection or analysis.

Statistical tests (specified in figure legends) were performed using GraphPad Prism 9.0.0 and R language (v4.4.1), including two‐tailed unpaired Student's *t*‐tests (pairwise comparisons), one‐way ANOVA (multiple groups), and log‐rank tests (survival analyses). For datasets not following a normal distribution, correlation coefficients were calculated using Spearman correlation analysis; the non‐parametric Kruskal–Wallis test was employed for multi‐group comparisons. Results are presented as mean ± SEM (n ≥ 3), with *p* < 0.05 considered statistically significant.

### 4.28 Code Availability

The initial data preprocessing and normalization steps followed our previously published workflow [50], implemented using the publicly available code (https://github.com/gjtlab/Genetic‐Determinants‐of‐Somatic‐Selection‐of‐Mutational‐Processes‐in‐3566‐Human‐Cancers).

## Author Contributions

Lu Chen, Fei Li, Zhaozhang Huang, Yaolin Zheng, Chunyi Gao, Jihuan Hou, Qiang Yu, Bowen Zheng, Wenqing Zhang, and Xiaoting Hong conducted molecular, cellular and animal experiments and analyzed the data; Jintao Guo and Runyang Li conducted the bioinformatic analyses. Mingjie Gao and Xuemei Chen conducted the structural modeling and molecular docking. Yali Zheng, Daxuan Wang, Qiyuan Li, Tianhui Hu, and Yan‐yan Zhan conceived and designed the study. Lu Chen, Jintao Guo, Fei Li, Mingjie Gao, Tianhui Hu and Yan‐yan Zhan wrote and revised the manuscript.

## Conflicts of Interest

The authors declare no conflicts of interest.

## Supporting information




**Supporting File 1**: advs75694‐sup‐0001‐SuppMat.pdf.

**Supporting File 2**: advs75694‐sup‐0002‐blots.pdf.

**Supporting File 3**: advs75694‐sup‐0003‐data.zip.

## Data Availability

The data that support the findings of this study are available in the supplementary material of this article.
