# Supplementary material for: FUCA2 Sustains AKT Signaling and Suppresses Senescence by Antagonizing FUT3‐Mediated ErbB3 Fucosylation in Lung Adenocarcinoma
Source: Adv Sci (Weinh). 2026 Jun 16;13(44):e23667. doi: 10.1002/advs.202523667 (PMC13335885; doi:10.1002/advs.202523667)

Extended Data Figure 2

a

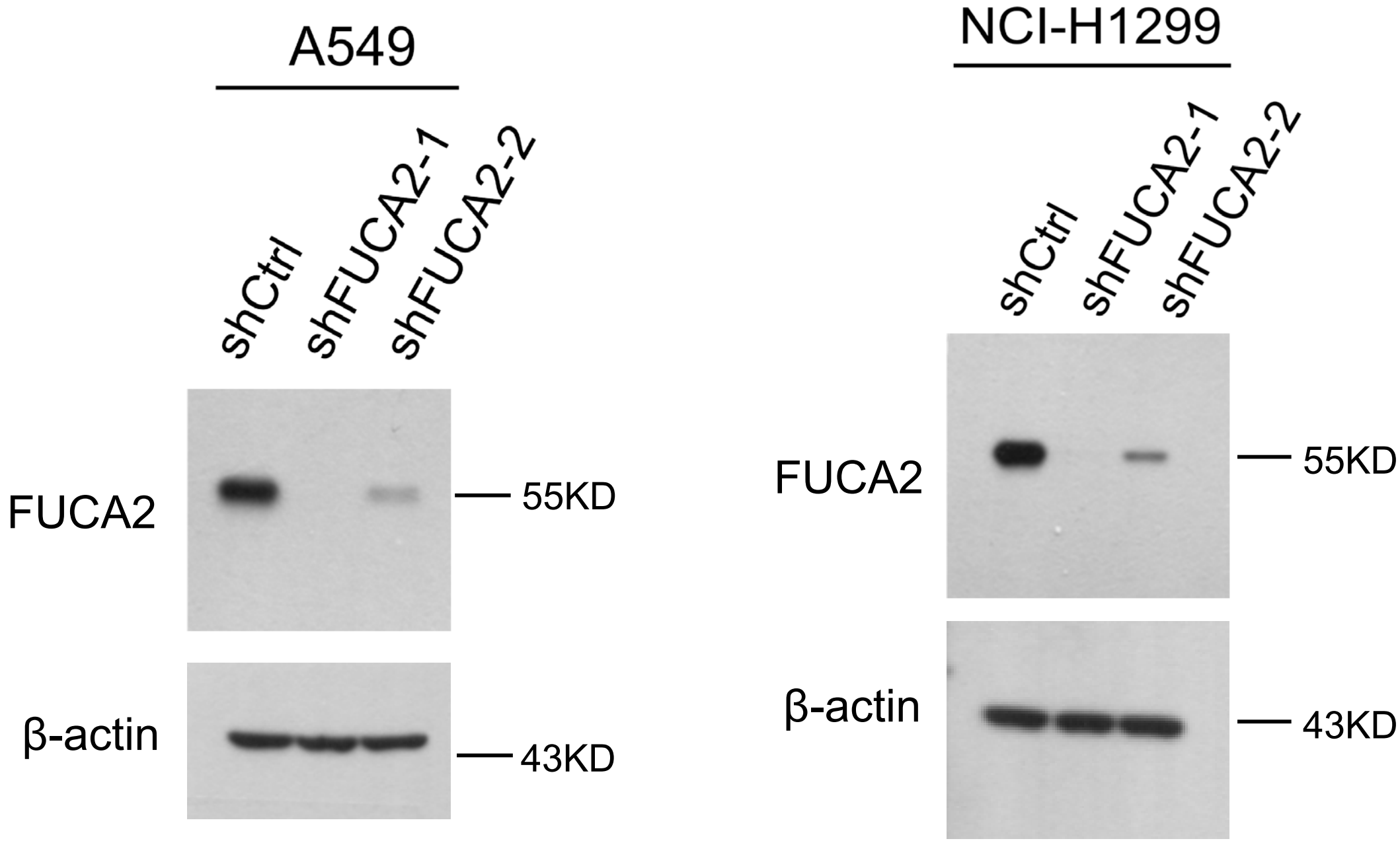

k

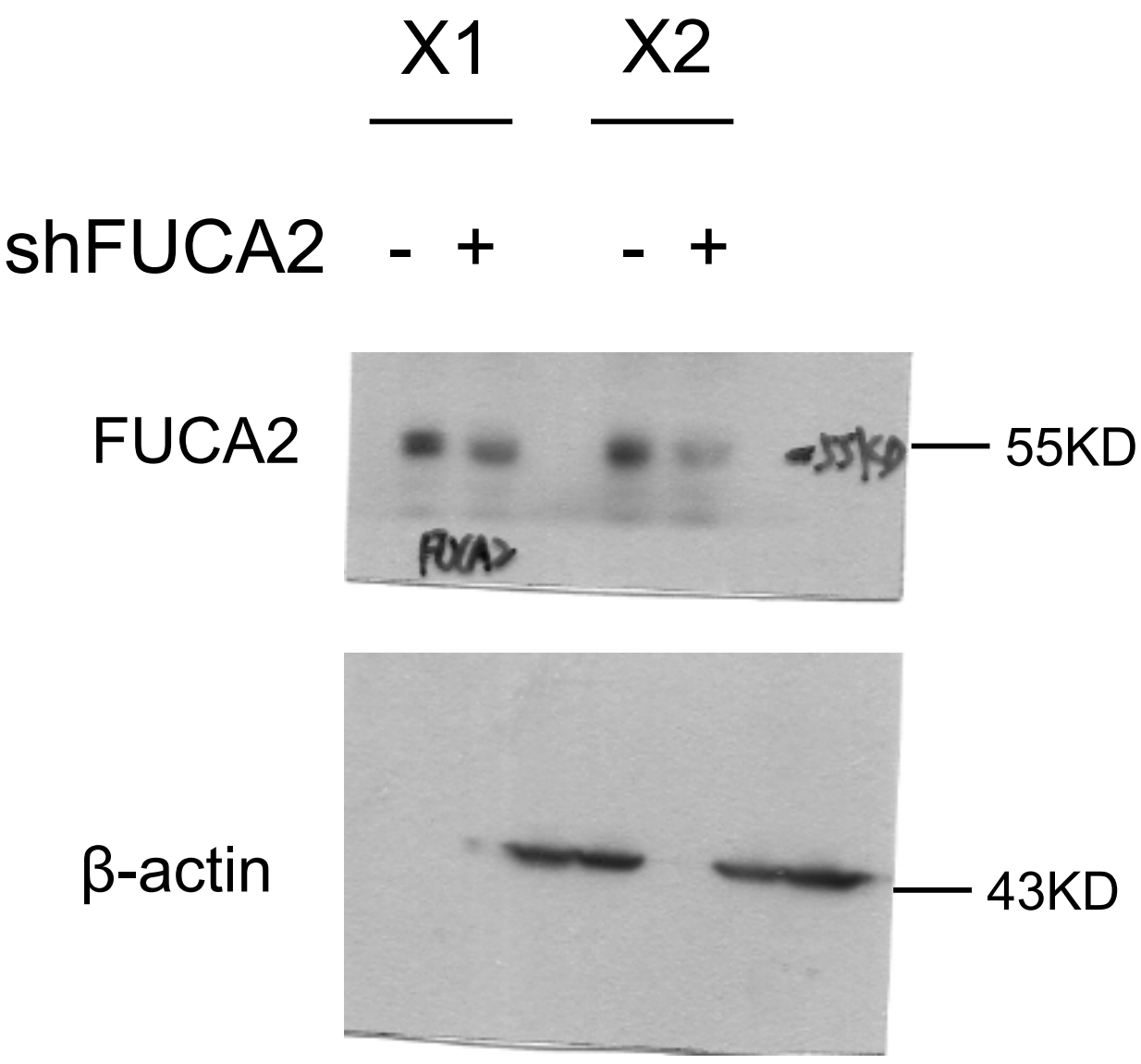

Figure 3

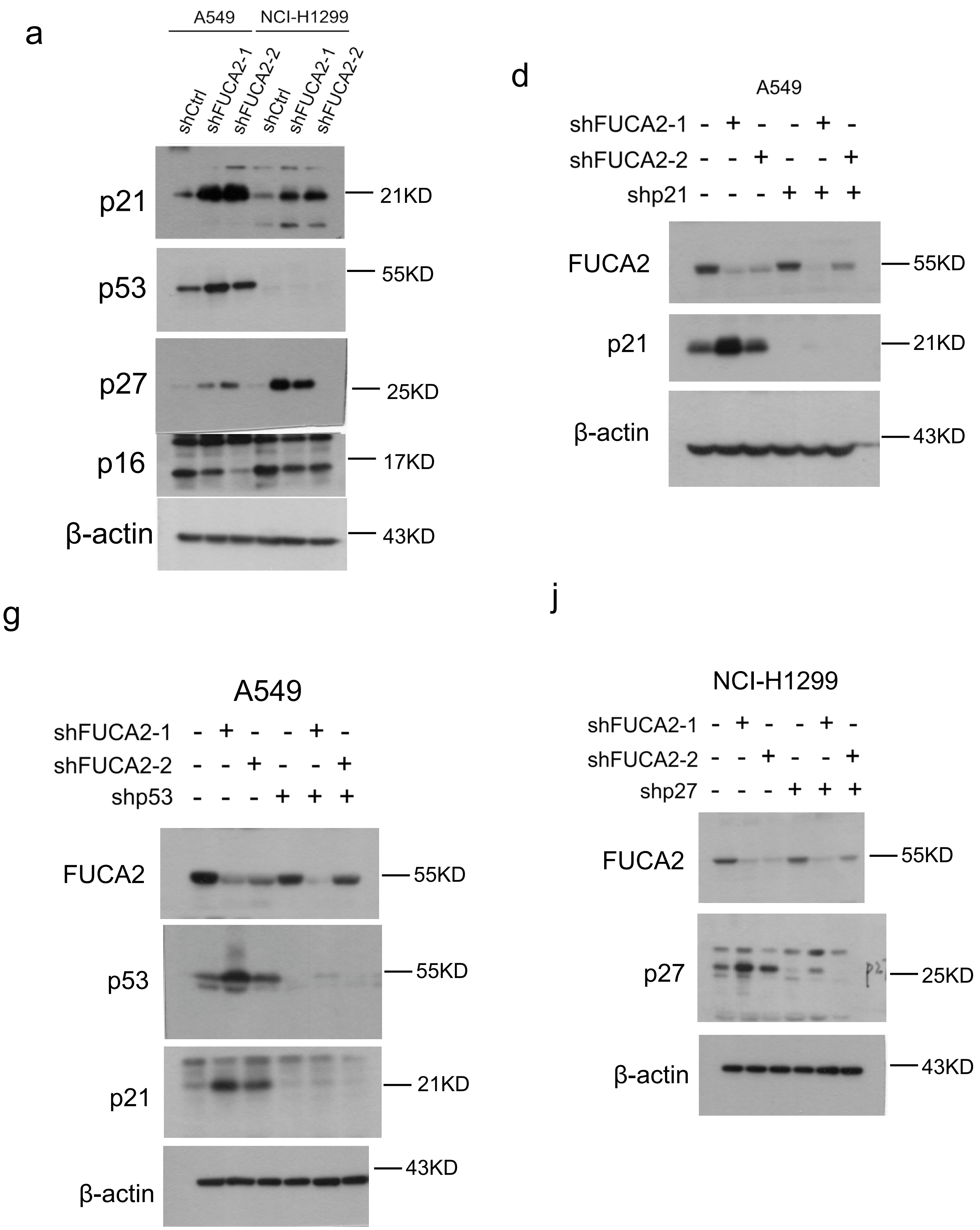

a
A549
NCI-H1299
shCtrl
shFUCA2-1
shFUCA2-2
shCtrl
shFUCA2-1
shFUCA2-2
p21
21KD
p53
55KD
p27
25KD
p16
17KD
β-actin
43KD
d
A549
shFUCA2-1 - + - - + -
shFUCA2-2 - - + - - +
shp21 - - - + + +
FUCA2
55KD
p21
21KD
β-actin
43KD
g
A549
shFUCA2-1 - + - - + -
shFUCA2-2 - - + - - +
shp53 - - - + + +
FUCA2
55KD
p53
55KD
p21
21KD
β-actin
43KD
j
NCI-H1299
shFUCA2-1 - + - - + -
shFUCA2-2 - - + - - +
shp27 - - - + + +
FUCA2
55KD
p27
25KD
β-actin
43KD

Figure 3

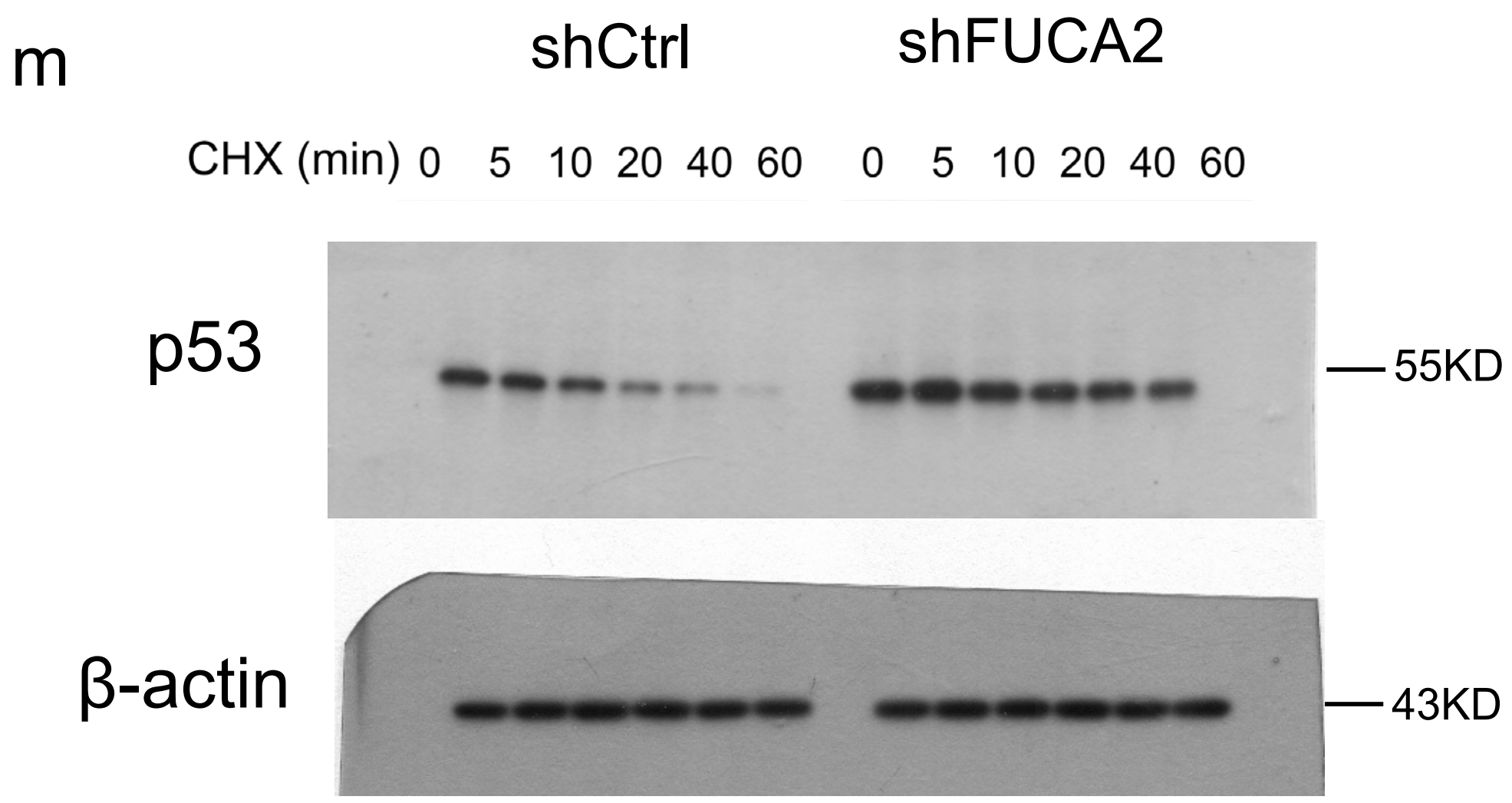
m
shCtrl
shFUCA2
CHX (min) 0 5 10 20 40 60 0 5 10 20 40 60
p53
55KD
β-actin
43KD

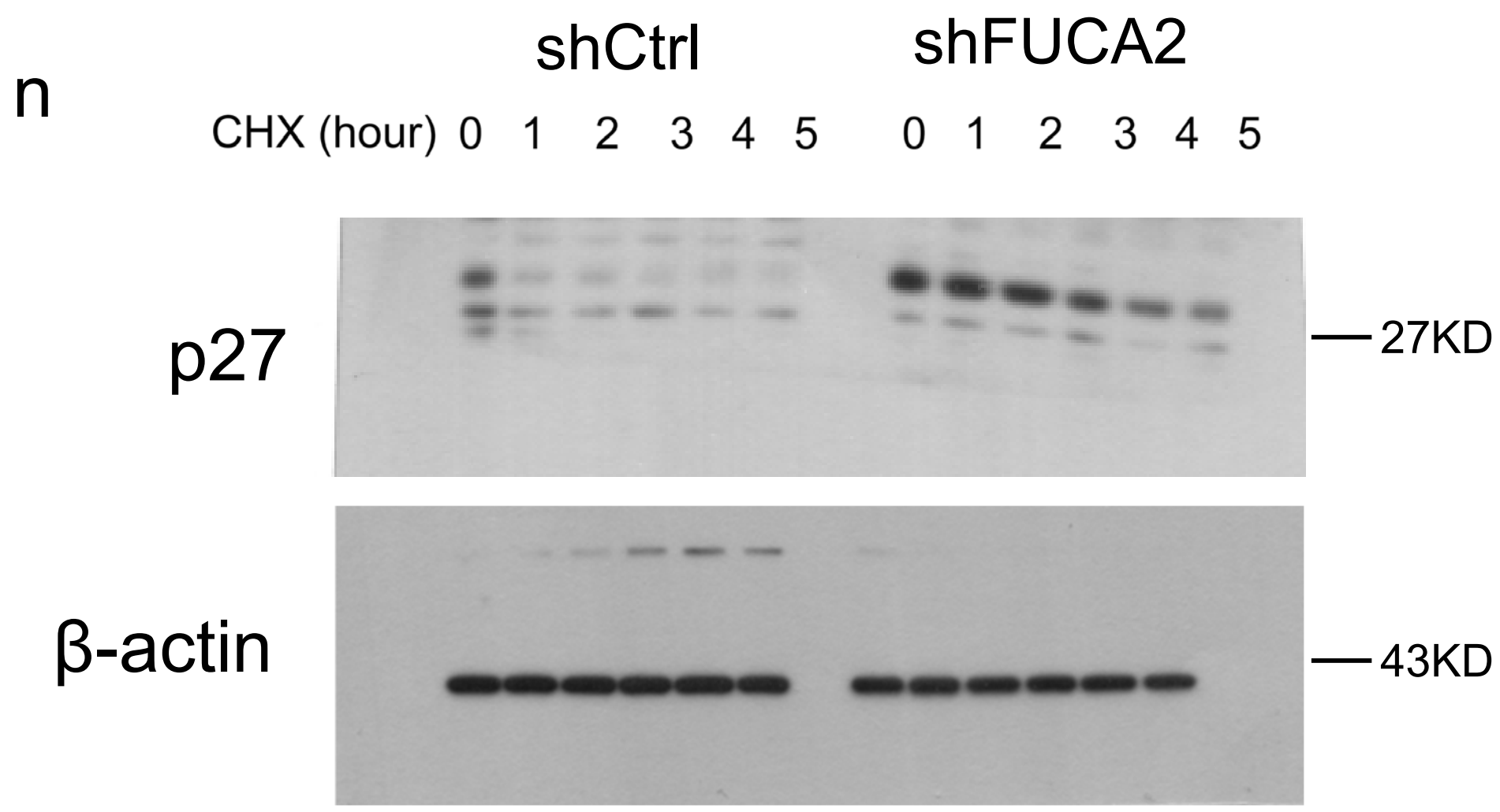
n
shCtrl
shFUCA2
CHX (hour) 0 1 2 3 4 5 0 1 2 3 4 5
p27
27KD
β-actin
43KD

Figure 4

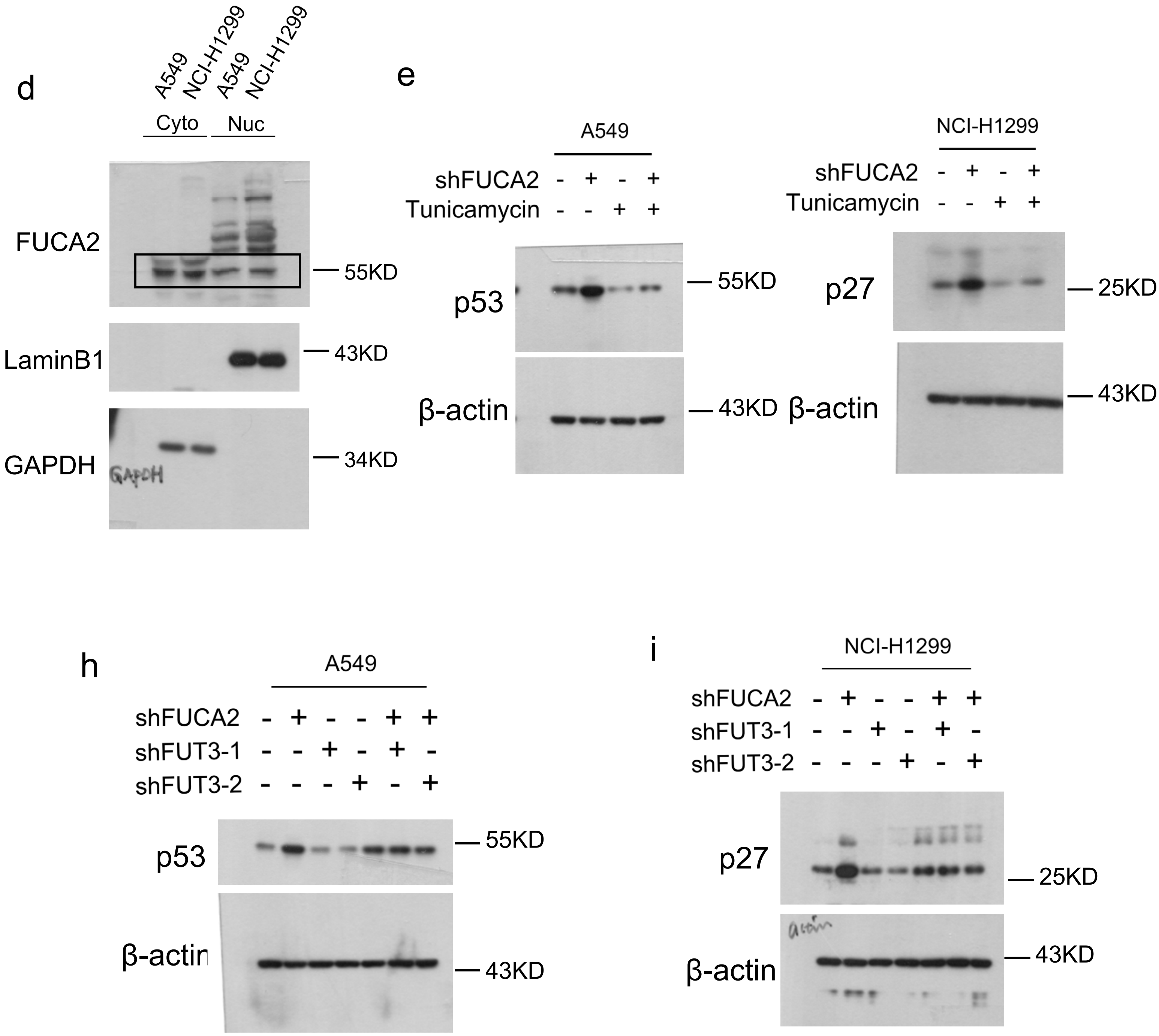
d
A549
NCI-H1299
A549
NCI-H1299
Cyto
Nuc
FUCA2
55KD
LaminB1
43KD
GAPDH
34KD
e
A549
shFUCA2 - + - +
Tunicamycin - - + +
p53
55KD
β-actin
43KD
NCI-H1299
shFUCA2 - + - +
Tunicamycin - - + +
p27
25KD
β-actin
43KD
h
A549
shFUCA2 - + - - + +
shFUT3-1 - - + - + -
shFUT3-2 - - - + - +
p53
55KD
β-actin
43KD
i
NCI-H1299
shFUCA2 - + - - + +
shFUT3-1 - - + - + -
shFUT3-2 - - - + - +
p27
25KD
β-actin
43KD

Figure 4

j

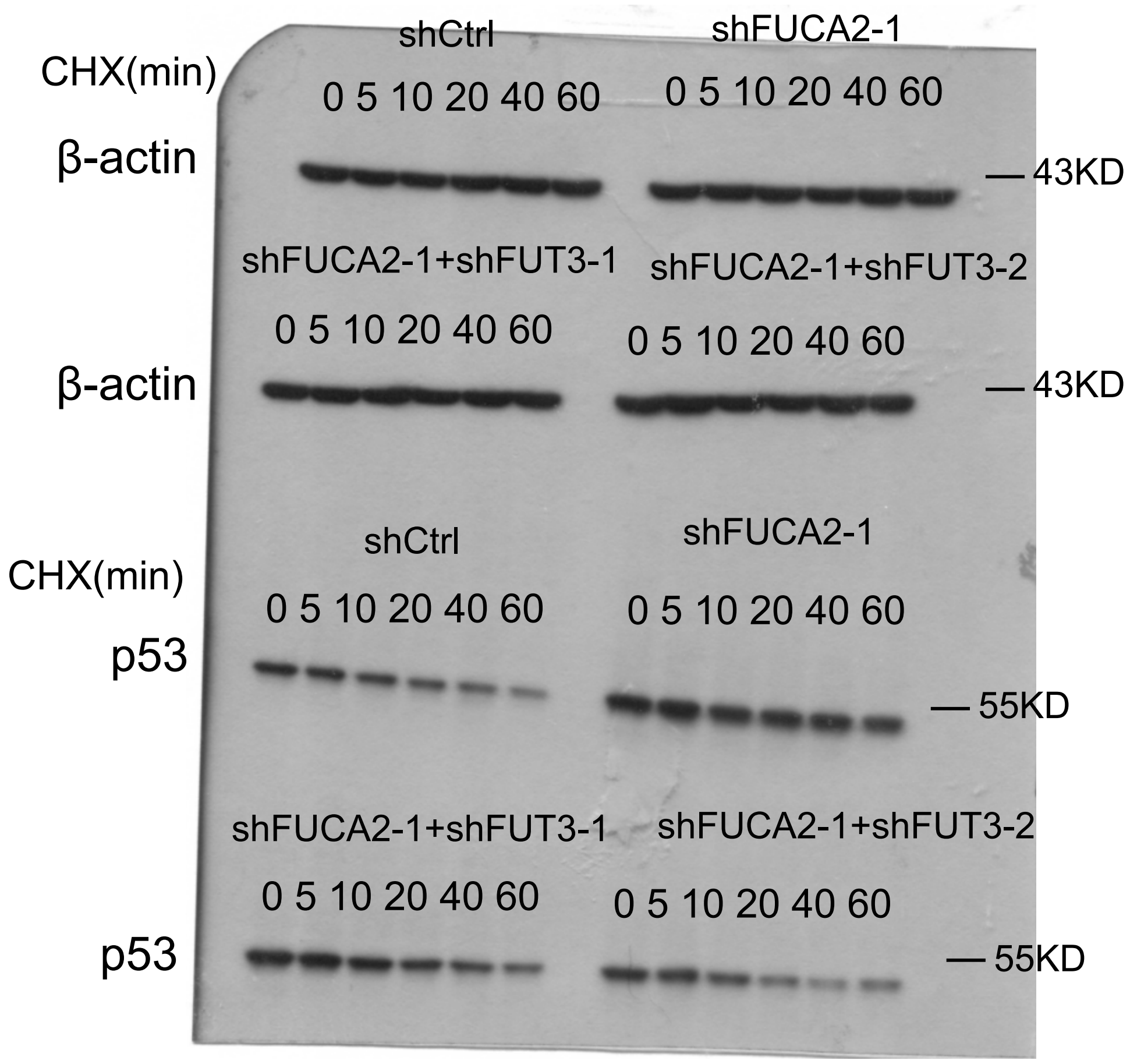
CHX(min)
shCtrl
shFUCA2-1
0 5 10 20 40 60
0 5 10 20 40 60
β-actin
— 43KD
shFUCA2-1+shFUT3-1
shFUCA2-1+shFUT3-2
0 5 10 20 40 60
0 5 10 20 40 60
β-actin
— 43KD
CHX(min)
shCtrl
shFUCA2-1
0 5 10 20 40 60
0 5 10 20 40 60
p53
— 55KD
shFUCA2-1+shFUT3-1
shFUCA2-1+shFUT3-2
0 5 10 20 40 60
0 5 10 20 40 60
p53
— 55KD

k

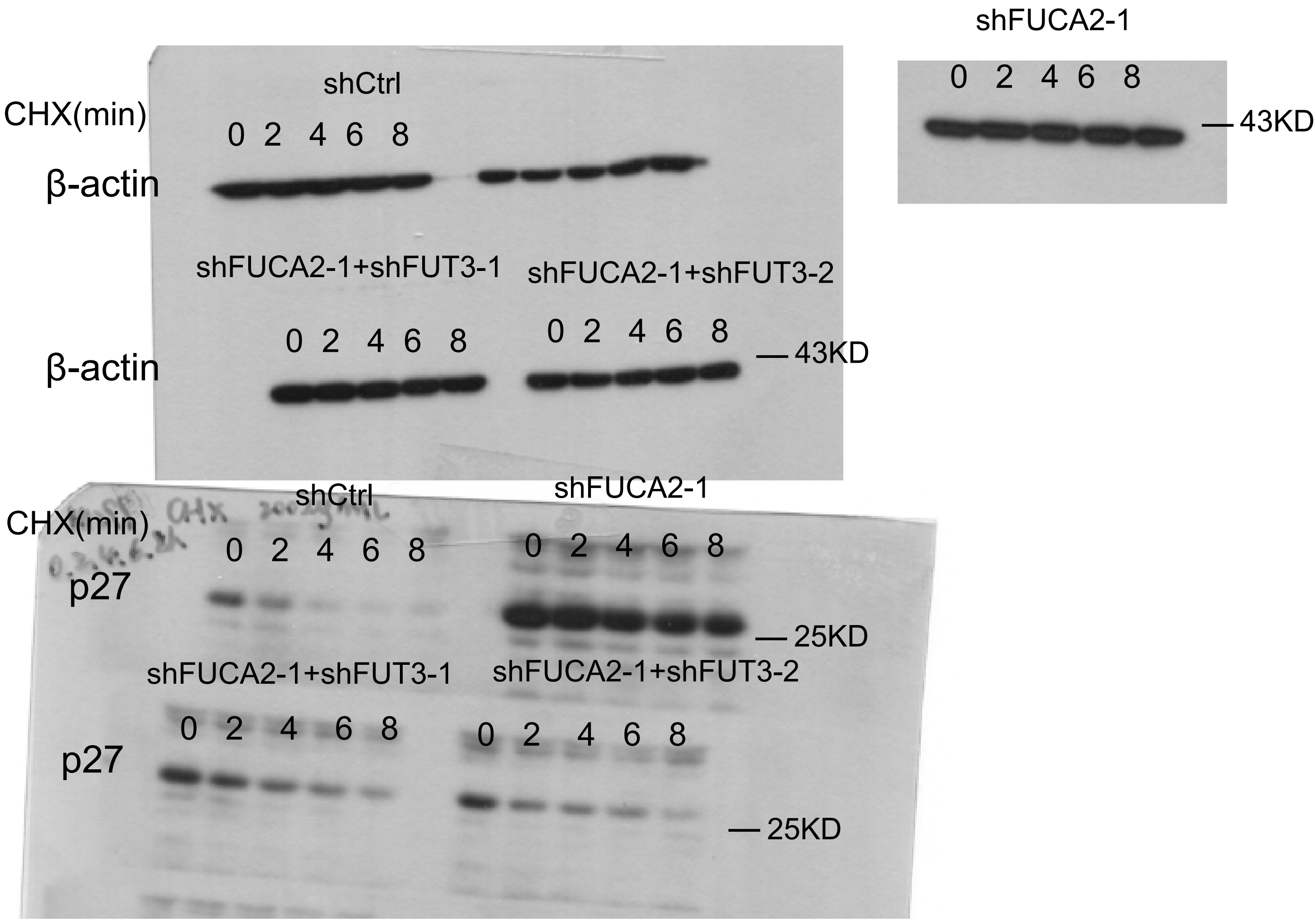
CHX(min)
shCtrl
0 2 4 6 8
β-actin
shFUCA2-1
0 2 4 6 8
— 43KD
shFUCA2-1+shFUT3-1
shFUCA2-1+shFUT3-2
0 2 4 6 8
0 2 4 6 8
β-actin
— 43KD
CHX(min)
shCtrl
shFUCA2-1
0 2 4 6 8
0 2 4 6 8
p27
— 25KD
shFUCA2-1+shFUT3-1
shFUCA2-1+shFUT3-2
0 2 4 6 8
0 2 4 6 8
p27
— 25KD

Extended Data Figure 3

a

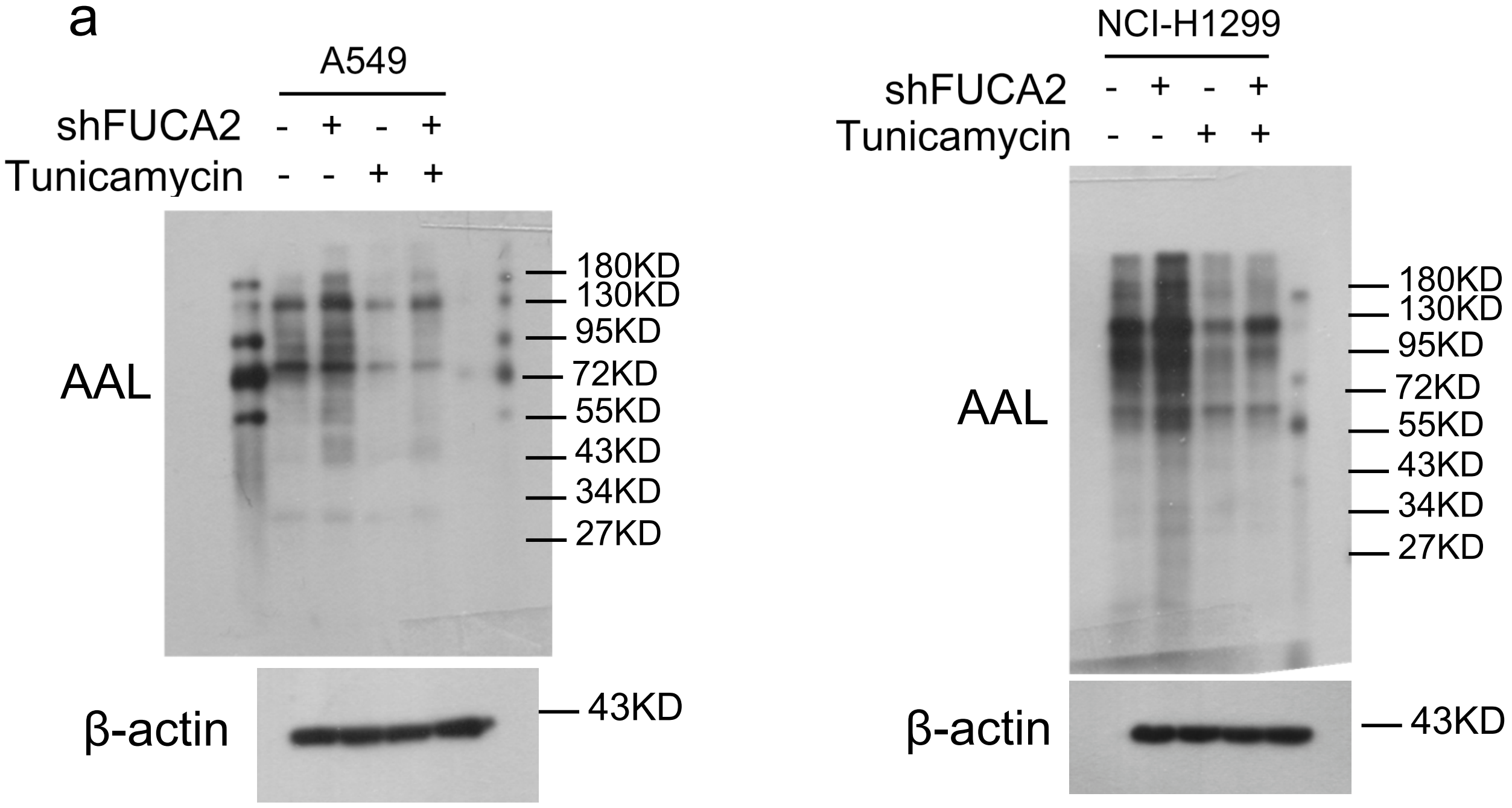

c

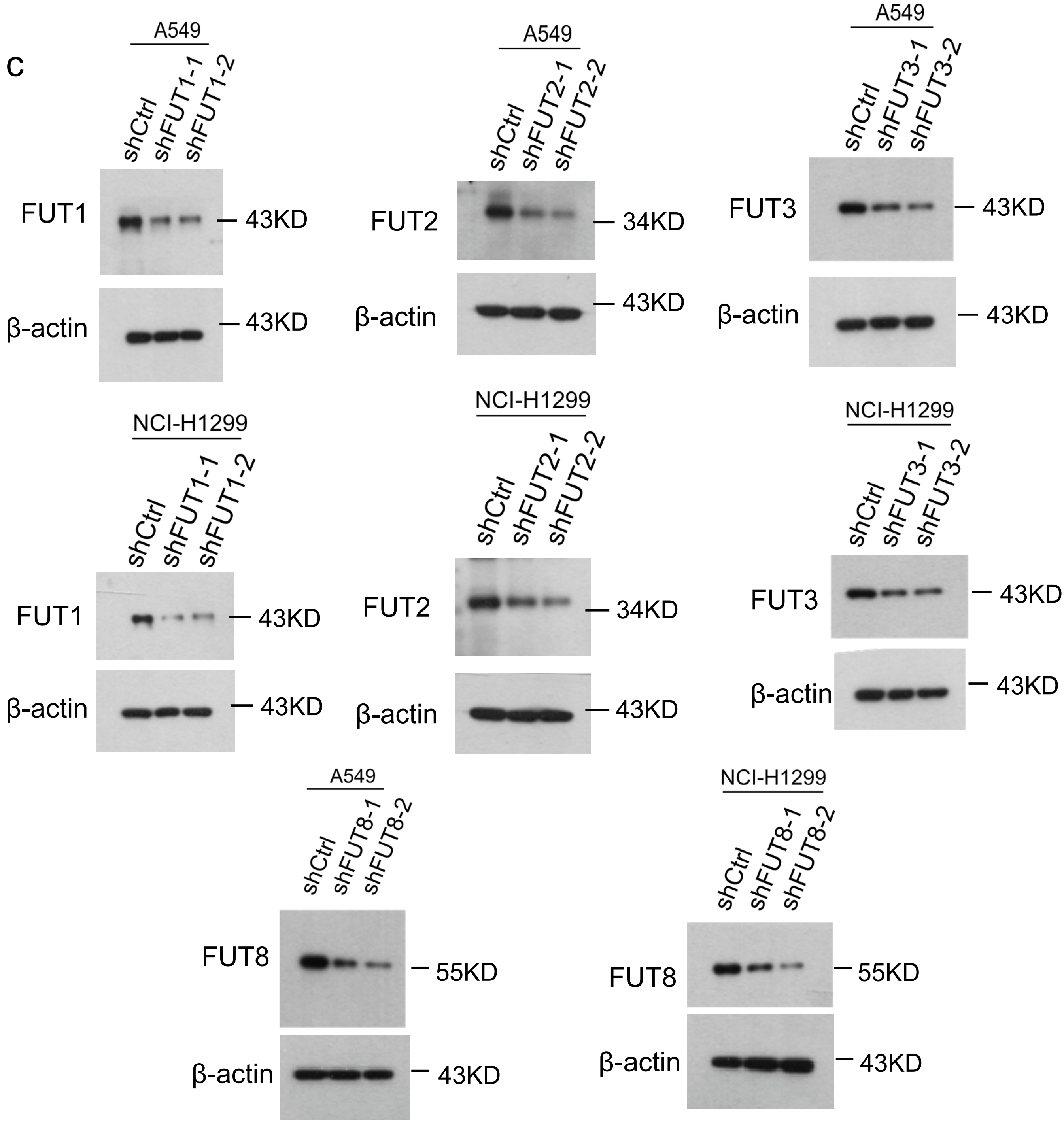

Figure 5

a

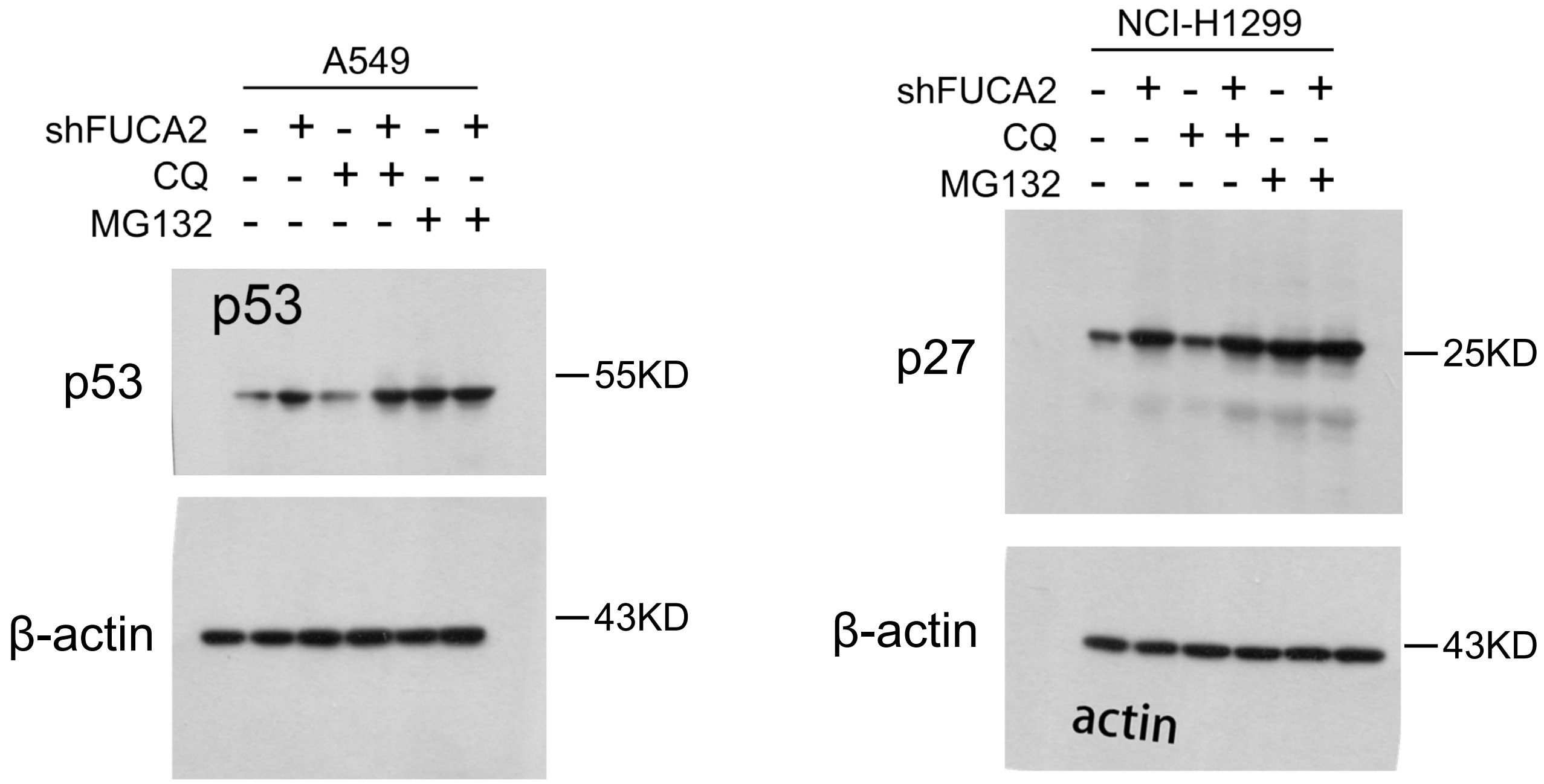
A549
shFUCA2 - + - + - +
CQ - - + + - -
MG132 - - - - + +
p53
p53
55KD
β-actin
43KD
NCI-H1299
shFUCA2 - + - + - +
CQ - - + + - -
MG132 - - - - + +
p27
25KD
β-actin
43KD
actin

Figure 5

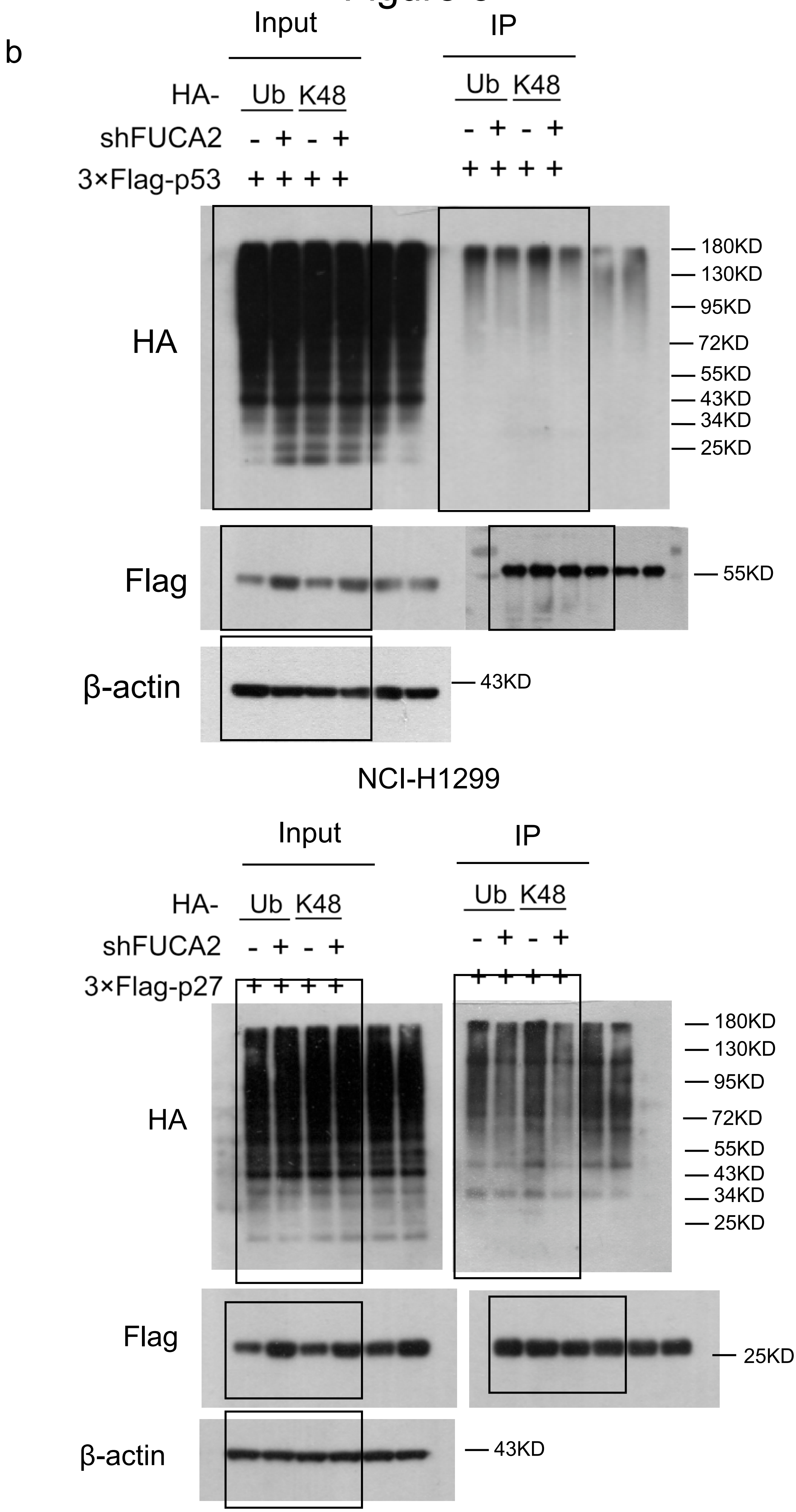
b
Input
IP
HA-
Ub K48
shFUCA2
- + - +
3×Flag-p53
+ + + +
HA
Flag
β-actin
180KD
130KD
95KD
72KD
55KD
43KD
34KD
25KD
NCI-H1299
3×Flag-p27

Figure 5

c

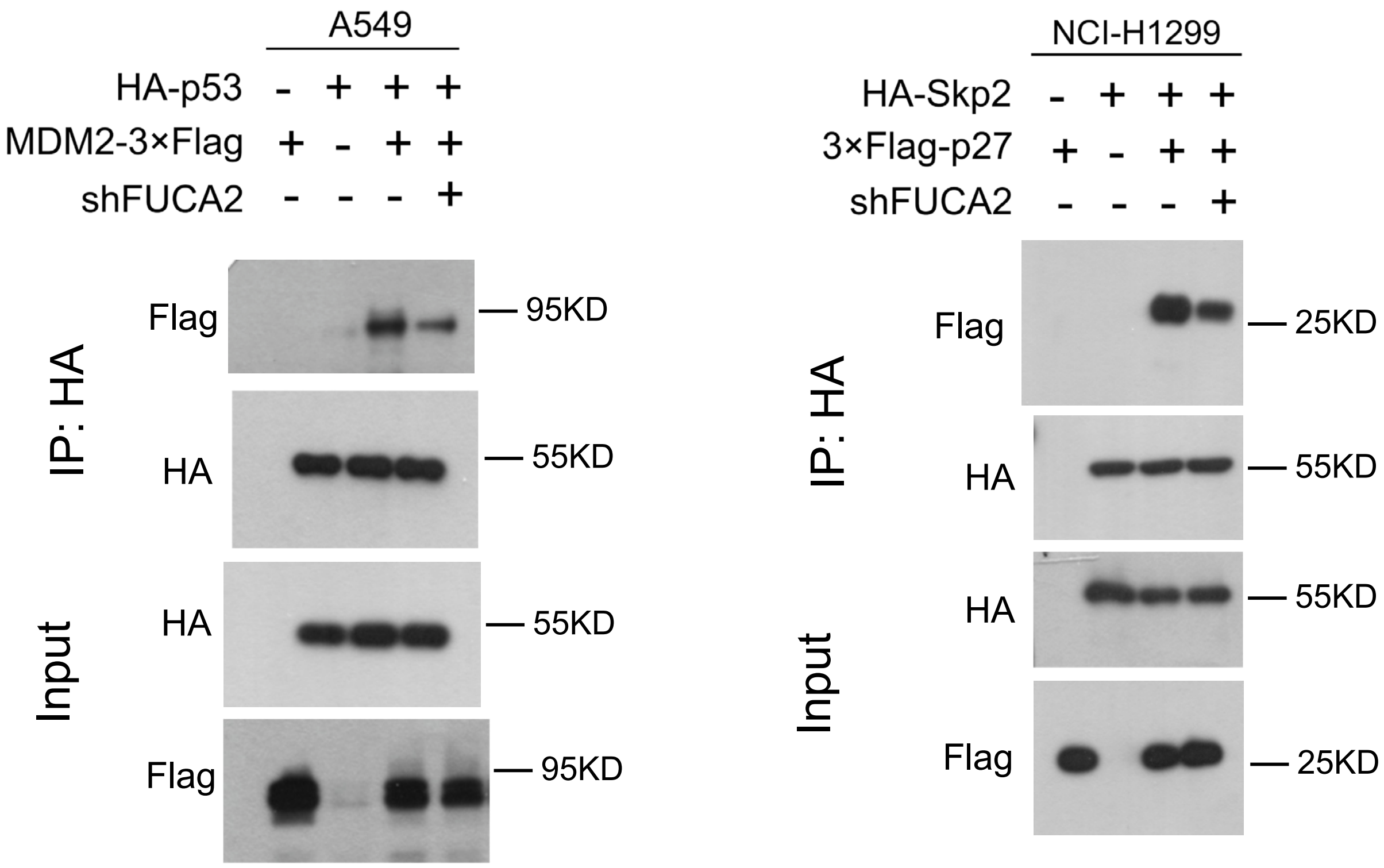
A549
HA-p53 - + + +
MDM2-3×Flag + - + +
shFUCA2 - - - +
Flag
95KD
IP: HA
HA
55KD
Input
HA
55KD
Flag
95KD
NCI-H1299
HA-Skp2 - + + +
3×Flag-p27 + - + +
shFUCA2 - - - +
Flag
25KD
IP: HA
HA
55KD
Input
HA
55KD
Flag
25KD

f

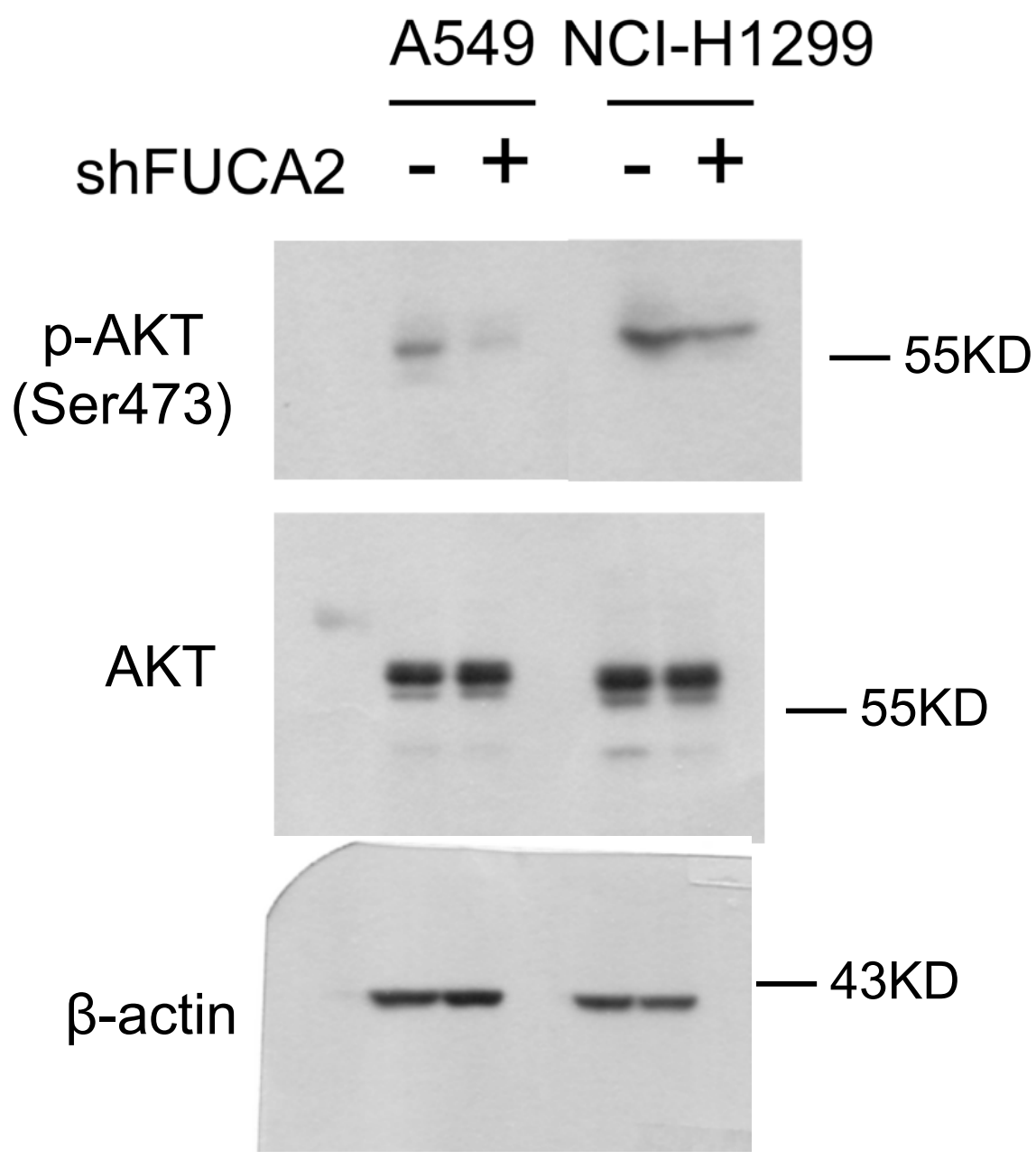
A549
NCI-H1299
shFUCA2 - + - +
p-AKT (Ser473)
55KD
AKT
55KD
β-actin
43KD

g

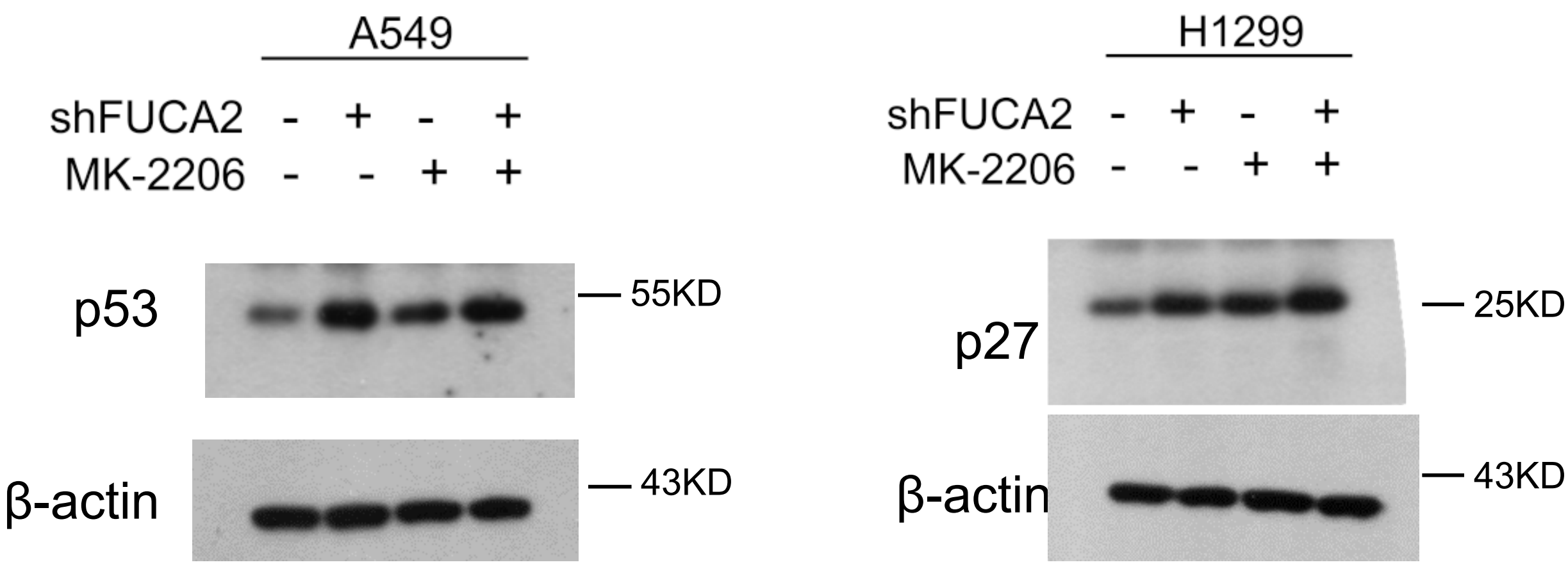
A549
shFUCA2 - + - +
MK-2206 - - + +
p53
55KD
β-actin
43KD
H1299
shFUCA2 - + - +
MK-2206 - - + +
p27
25KD
β-actin
43KD

Figure 5

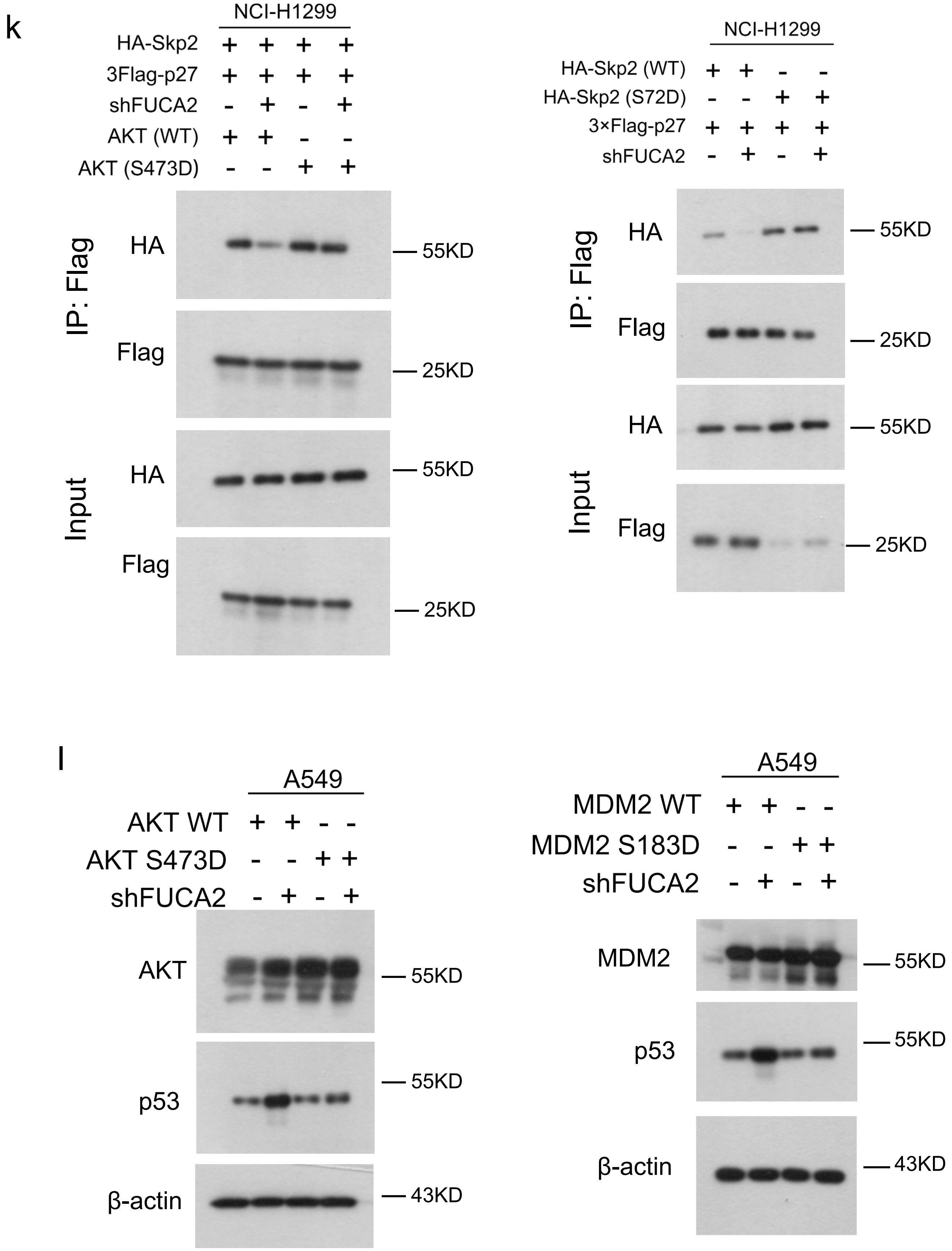

k
NCI-H1299
HA-Skp2 + + + +
3Flag-p27 + + + +
shFUCA2 - + - +
AKT (WT) + + - -
AKT (S473D) - - + +
IP: Flag
HA
55KD
Flag
25KD
Input
HA
55KD
Flag
25KD
NCI-H1299
HA-Skp2 (WT) + + - -
HA-Skp2 (S72D) - - + +
3×Flag-p27 + + + +
shFUCA2 - + - +
IP: Flag
HA
55KD
Flag
25KD
HA
55KD
Input
Flag
25KD
l
A549
AKT WT + + - -
AKT S473D - - + +
shFUCA2 - + - +
AKT
55KD
p53
55KD
β-actin
43KD
A549
MDM2 WT + + - -
MDM2 S183D - - + +
shFUCA2 - + - +
MDM2
55KD
p53
55KD
β-actin
43KD

Figure 5

m

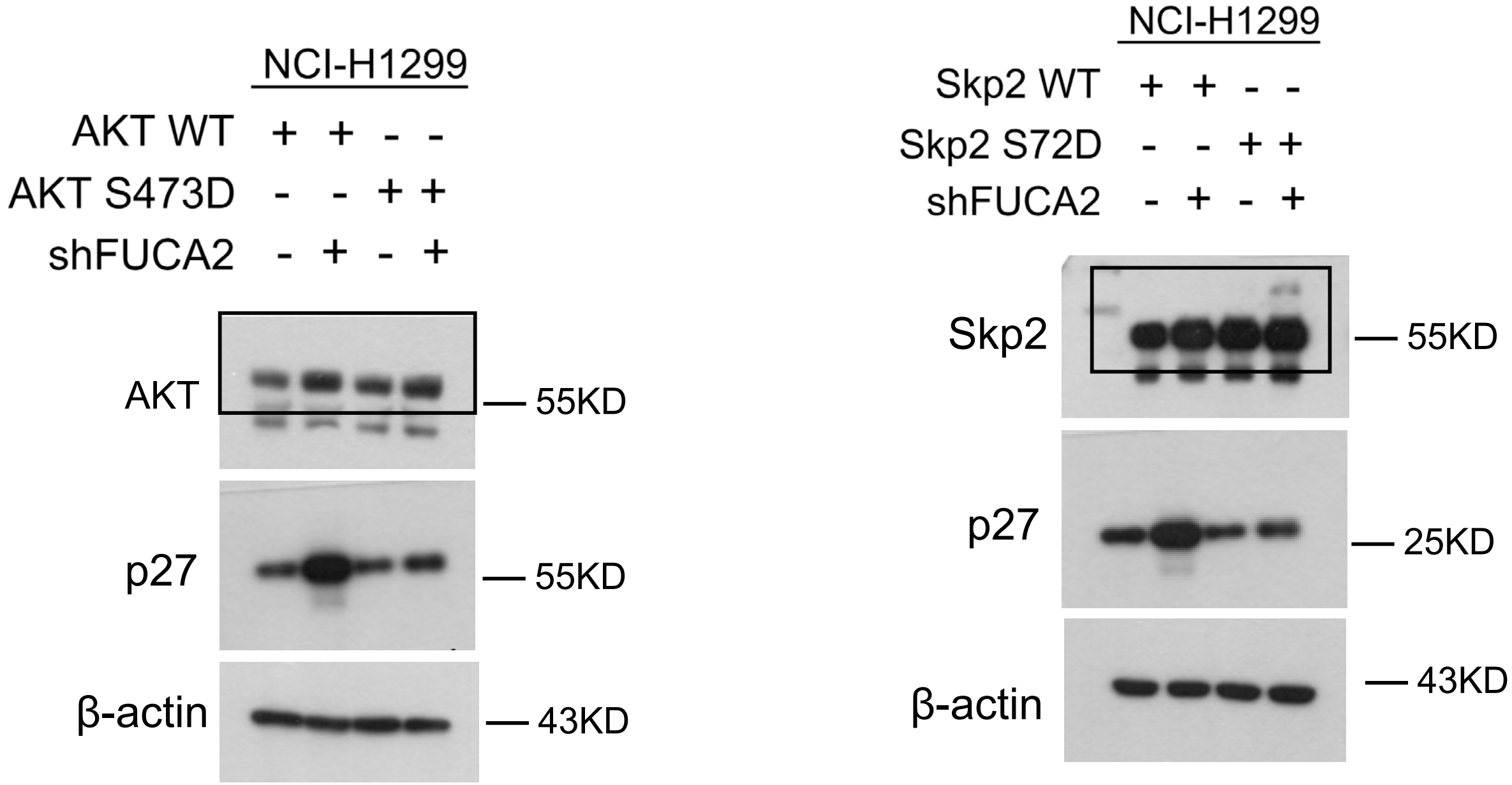
NCI-H1299
AKT WT + + - -
AKT S473D - - + +
shFUCA2 - + - +
AKT
55KD
p27
55KD
β-actin
43KD
NCI-H1299
Skp2 WT + + - -
Skp2 S72D - - + +
shFUCA2 - + - +
Skp2
55KD
p27
25KD
β-actin
43KD

Extended Data Figure 4

a

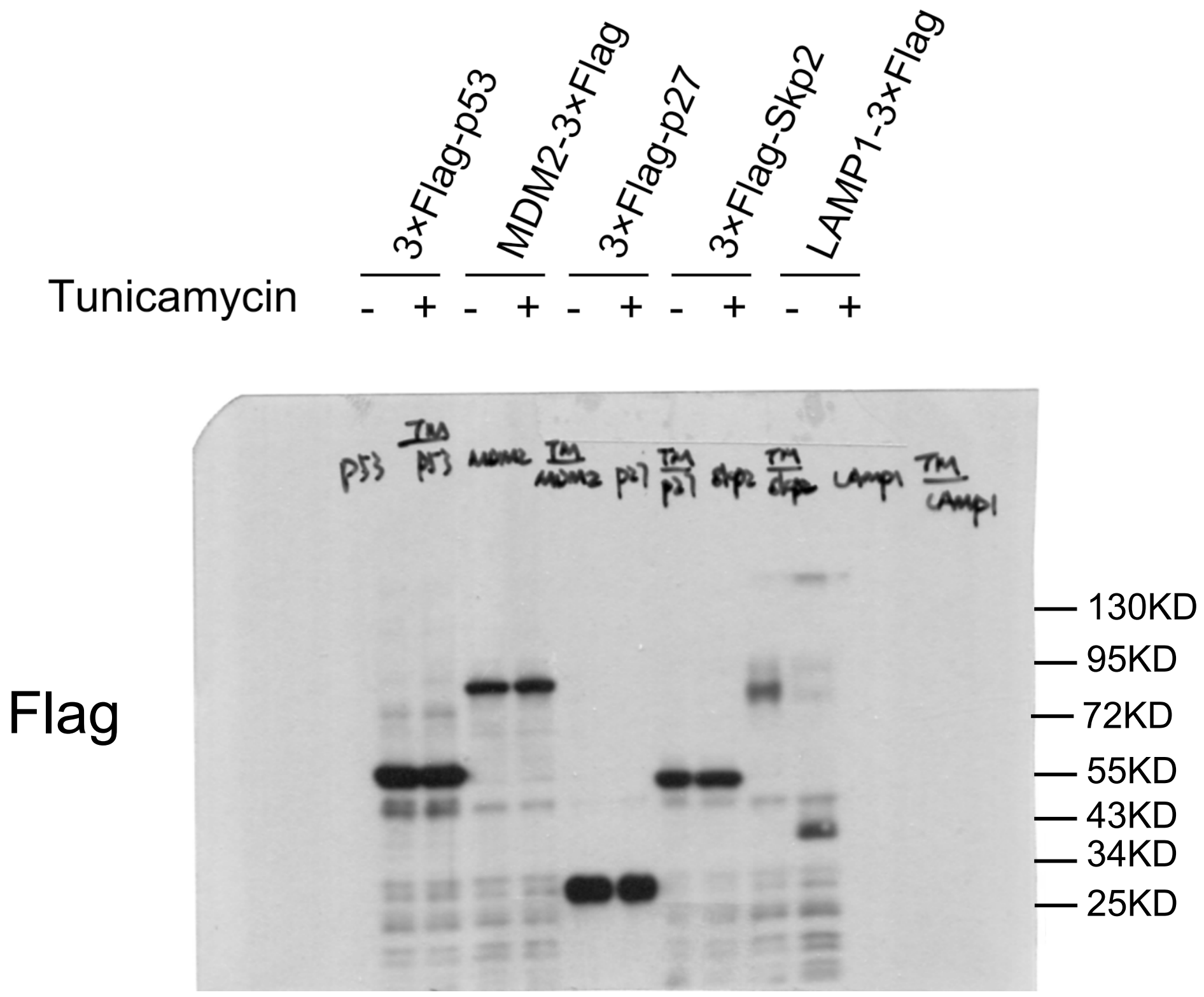

c

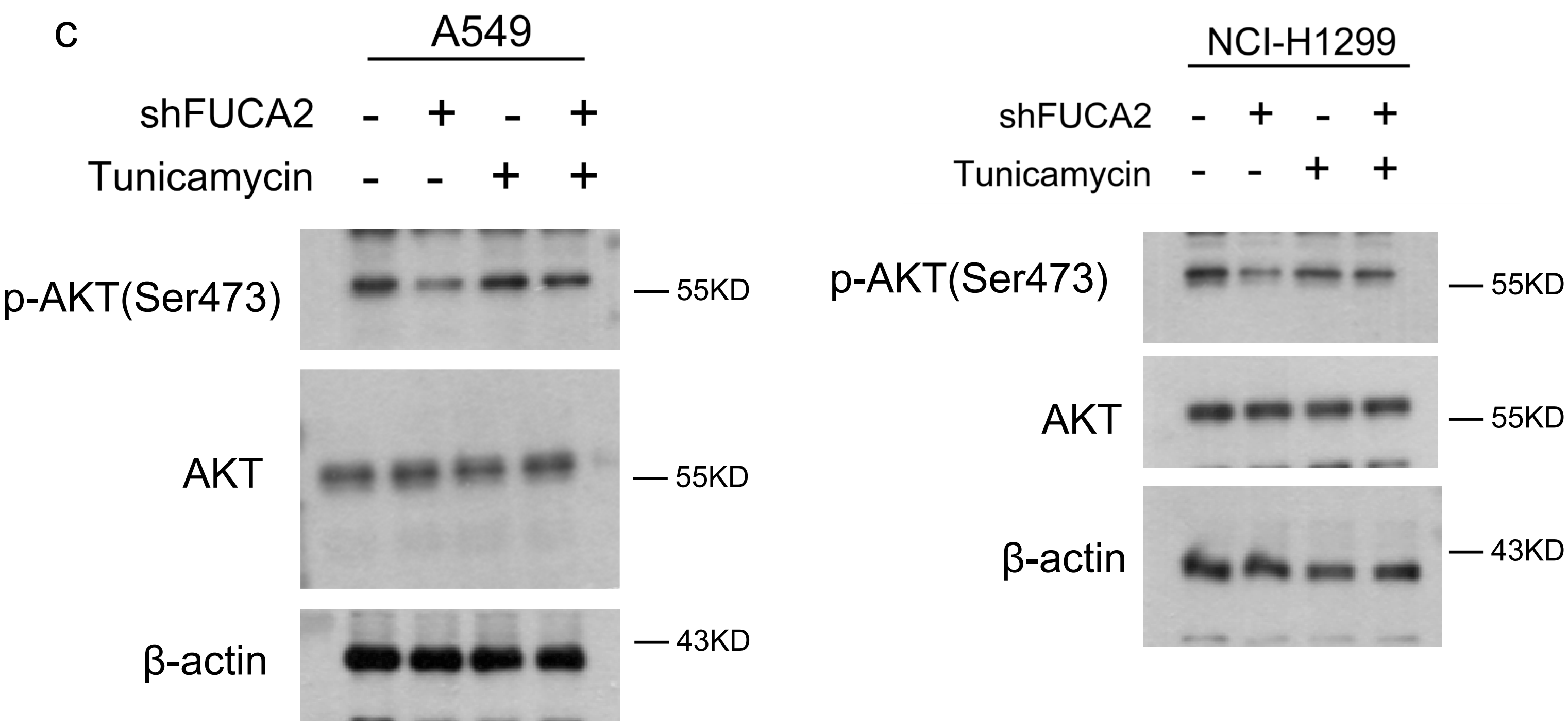

Figure 6

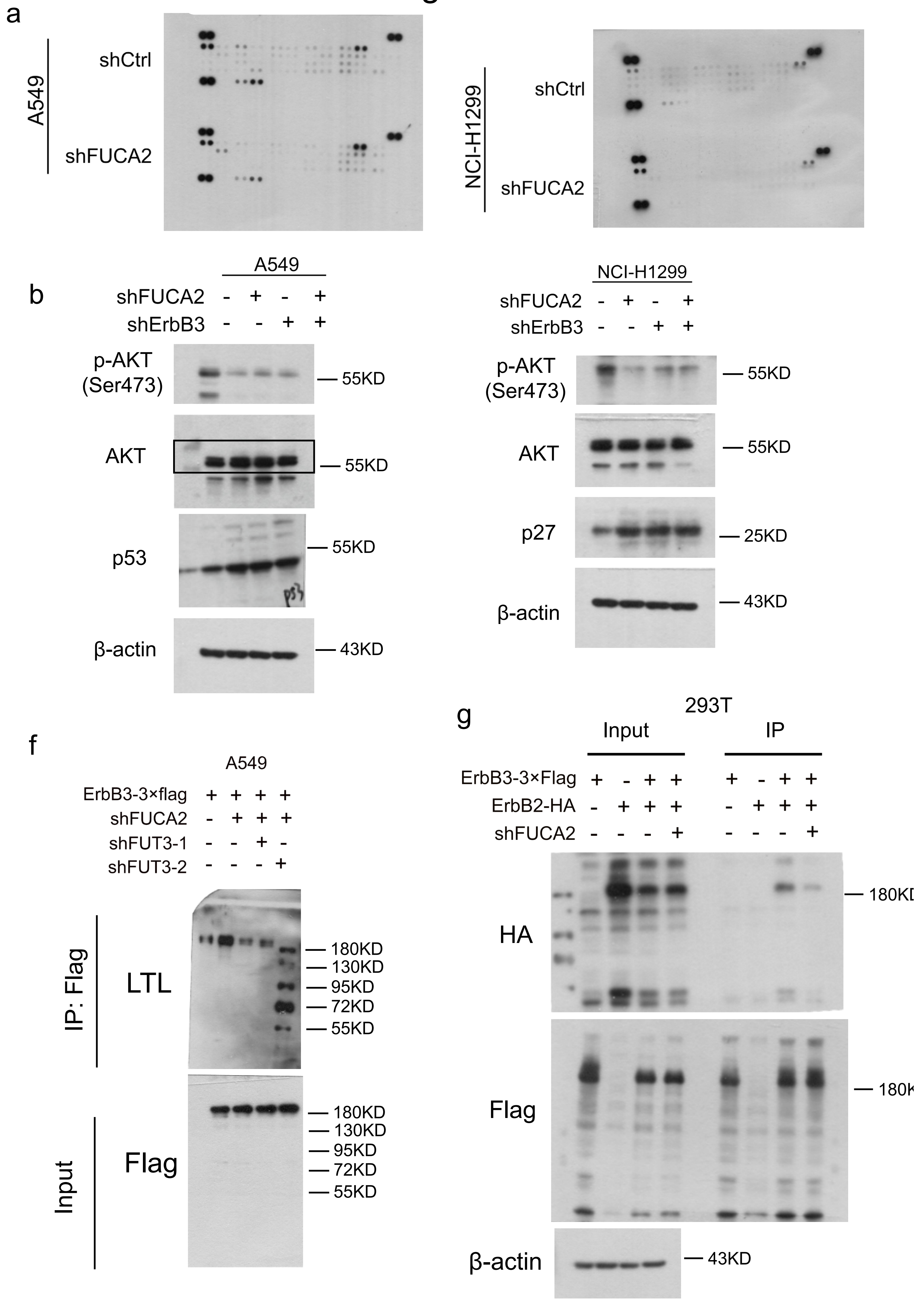
a
A549
shCtrl
shFUCA2
NCI-H1299
shCtrl
shFUCA2
b
A549
shFUCA2 - + - +
shErbB3 - - + +
p-AKT (Ser473)
55KD
AKT
55KD
p53
55KD
β-actin
43KD
NCI-H1299
shFUCA2 - + - +
shErbB3 - - + +
p-AKT (Ser473)
55KD
AKT
55KD
p27
25KD
β-actin
43KD
f
A549
ErbB3-3×flag + + + +
shFUCA2 - + + +
shFUT3-1 - - + -
shFUT3-2 - - - +
IP: Flag
LTL
180KD
130KD
95KD
72KD
55KD
Input
Flag
180KD
130KD
95KD
72KD
55KD
g
293T
Input
IP
ErbB3-3×Flag + - + + + - + +
ErbB2-HA - + + + - + + +
shFUCA2 - - - + - - - +
HA
180KD
Flag
β-actin
43KD

Figure 6

h

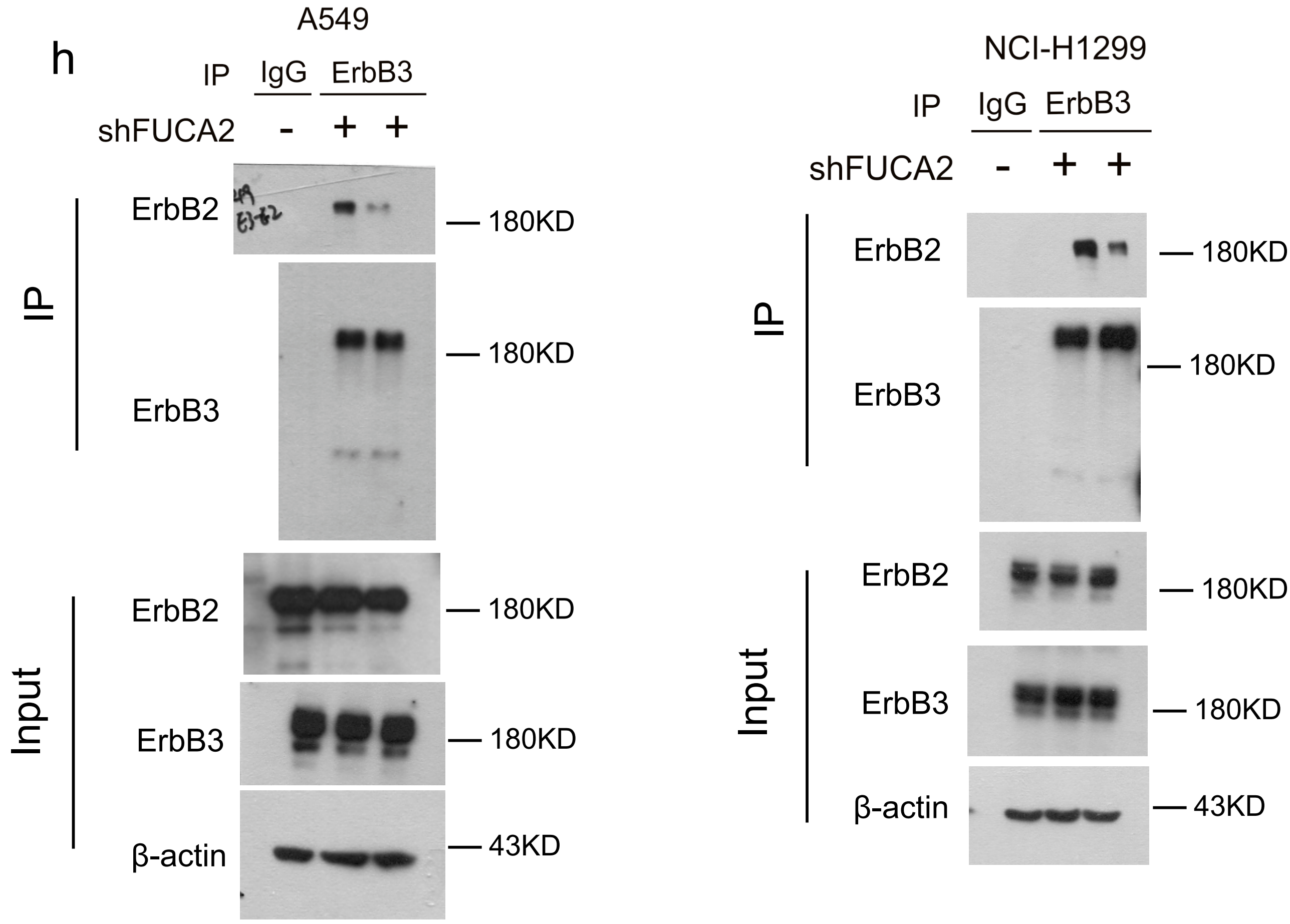
A549
IP IgG ErbB3
shFUCA2 - + +
IP
ErbB2
180KD
ErbB3
180KD
Input
ErbB2
180KD
ErbB3
180KD
β-actin
43KD
NCI-H1299
IP IgG ErbB3
shFUCA2 - + +
IP
ErbB2
180KD
ErbB3
180KD
Input
ErbB2
180KD
ErbB3
180KD
β-actin
43KD

i

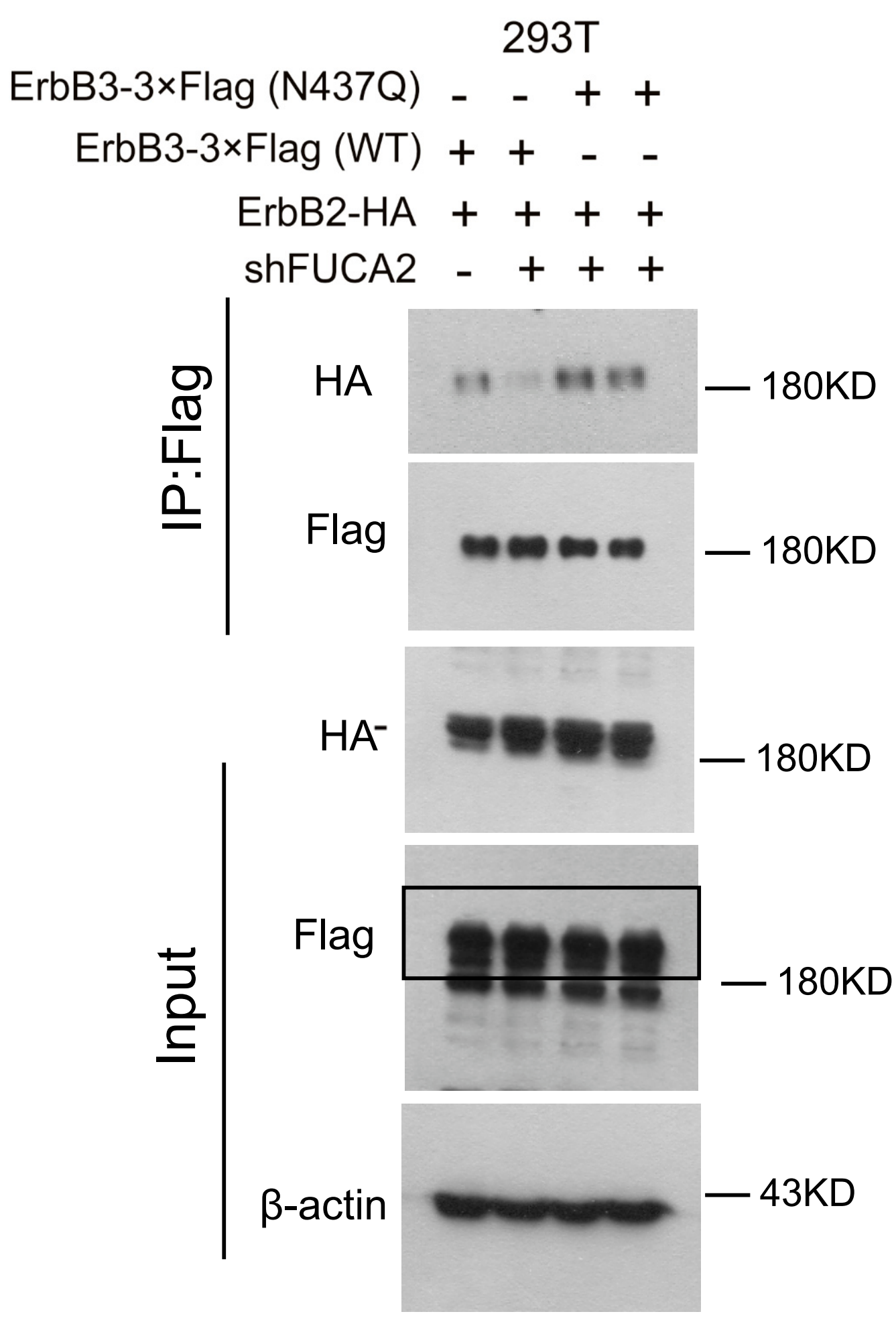
293T
ErbB3-3×Flag (N437Q) - - + +
ErbB3-3×Flag (WT) + + - -
ErbB2-HA + + + +
shFUCA2 - + + +
IP:Flag
HA
180KD
Flag
180KD
Input
HA
180KD
Flag
180KD
β-actin
43KD

Figure 6

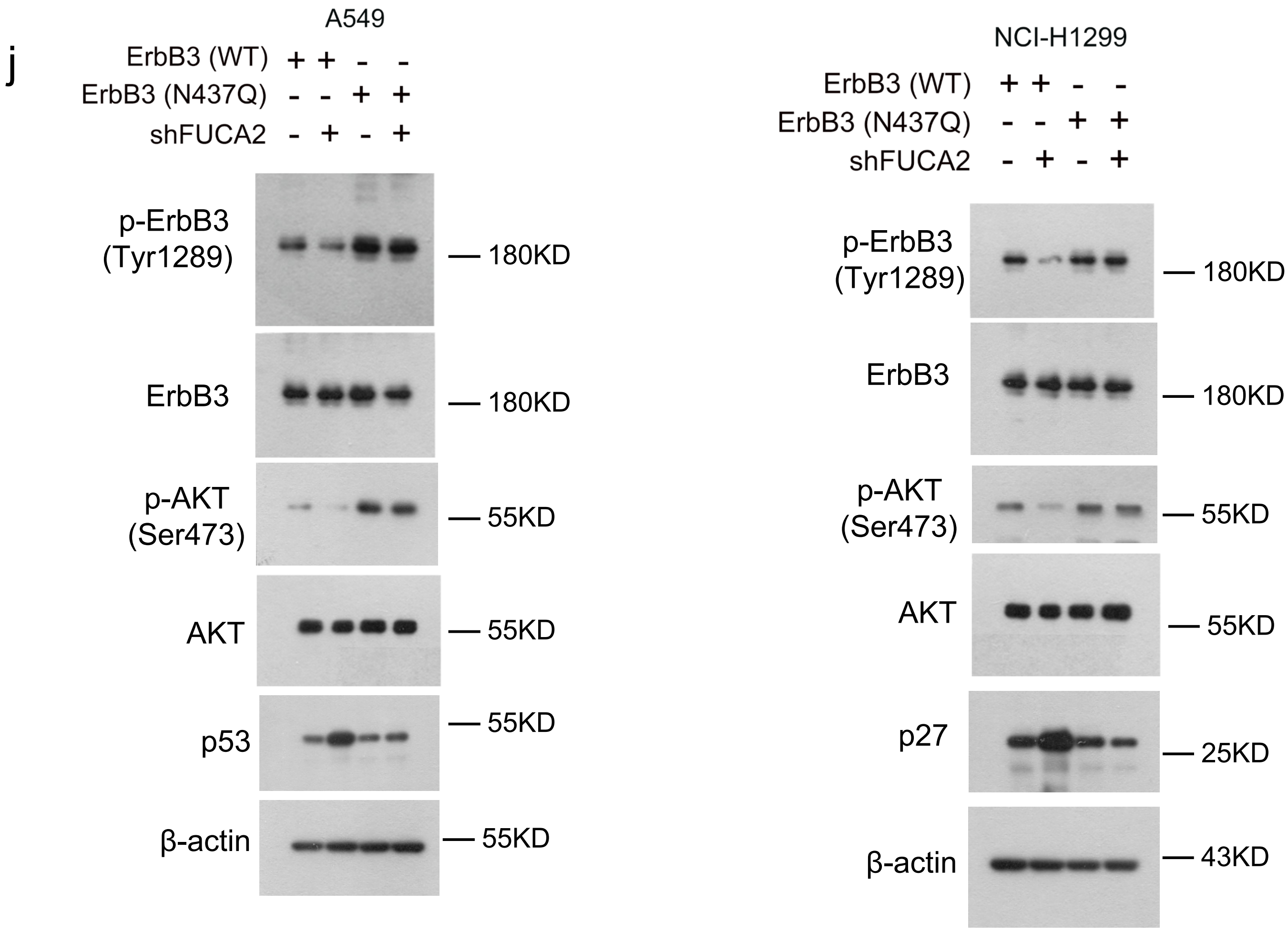
j
A549
ErbB3 (WT) + + - -
ErbB3 (N437Q) - - + +
shFUCA2 - + - +
p-ErbB3 (Tyr1289)
180KD
ErbB3
180KD
p-AKT (Ser473)
55KD
AKT
55KD
p53
55KD
β-actin
55KD
NCI-H1299
ErbB3 (WT) + + - -
ErbB3 (N437Q) - - + +
shFUCA2 - + - +
p-ErbB3 (Tyr1289)
180KD
ErbB3
180KD
p-AKT (Ser473)
55KD
AKT
55KD
p27
25KD
β-actin
43KD

Figure 6

I

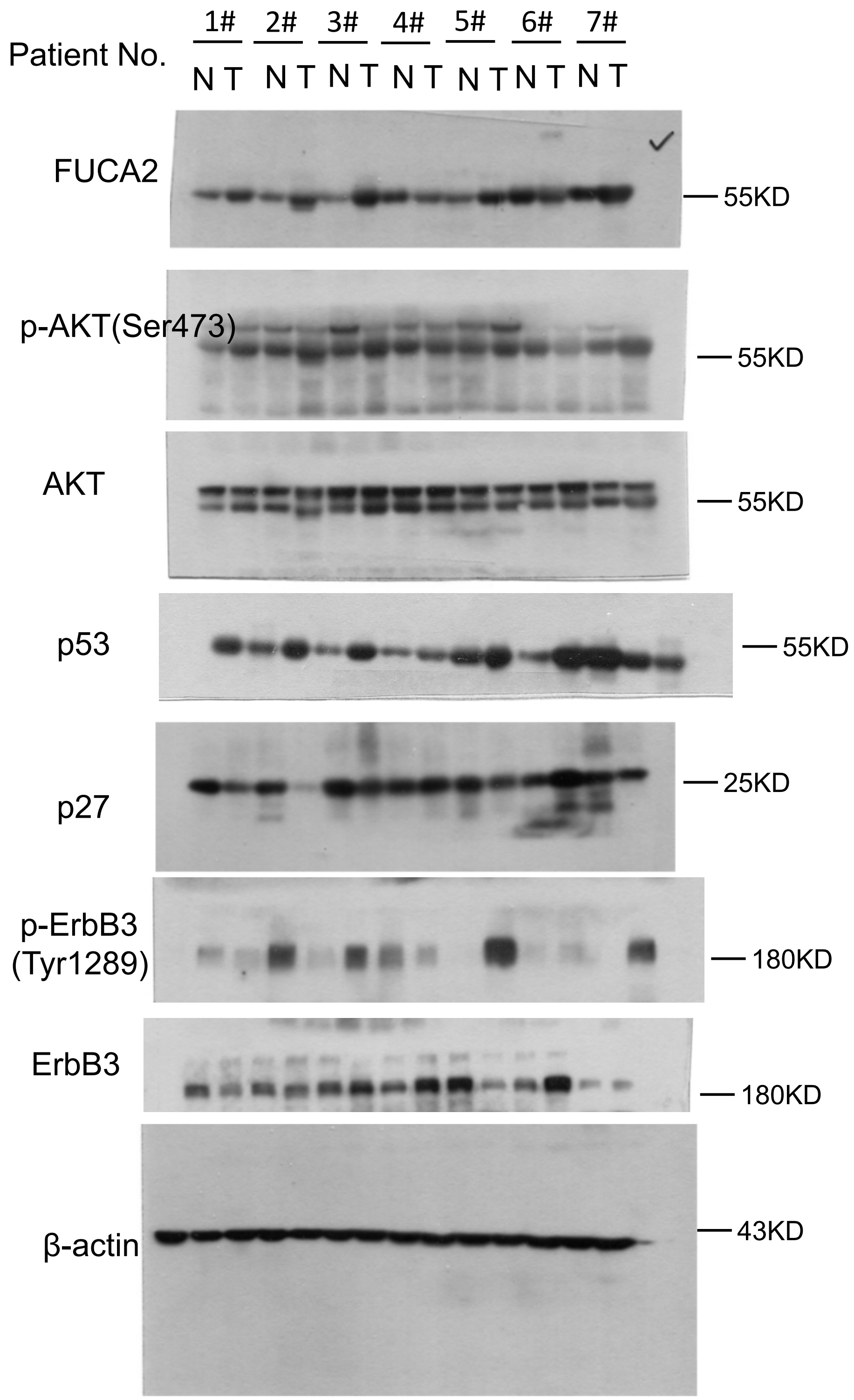

Patient No.
1# 2# 3# 4# 5# 6# 7#
N T N T N T N T N T N T N T
FUCA2
55KD
p-AKT(Ser473)
55KD
AKT
55KD
p53
55KD
p27
25KD
p-ErbB3
(Tyr1289)
180KD
ErbB3
180KD
β-actin
43KD

Extended Data Figure 5

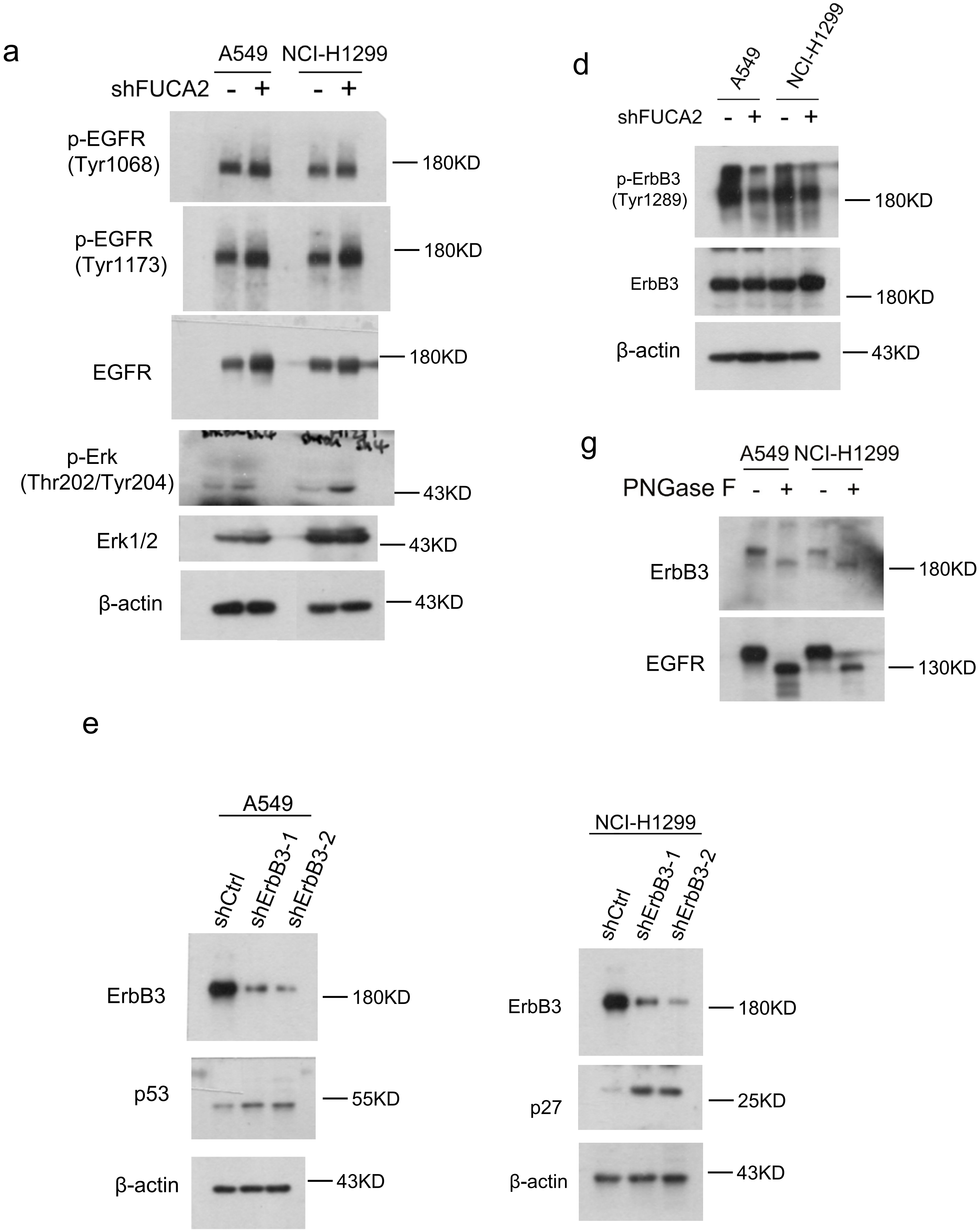

Extended Data Figure 5

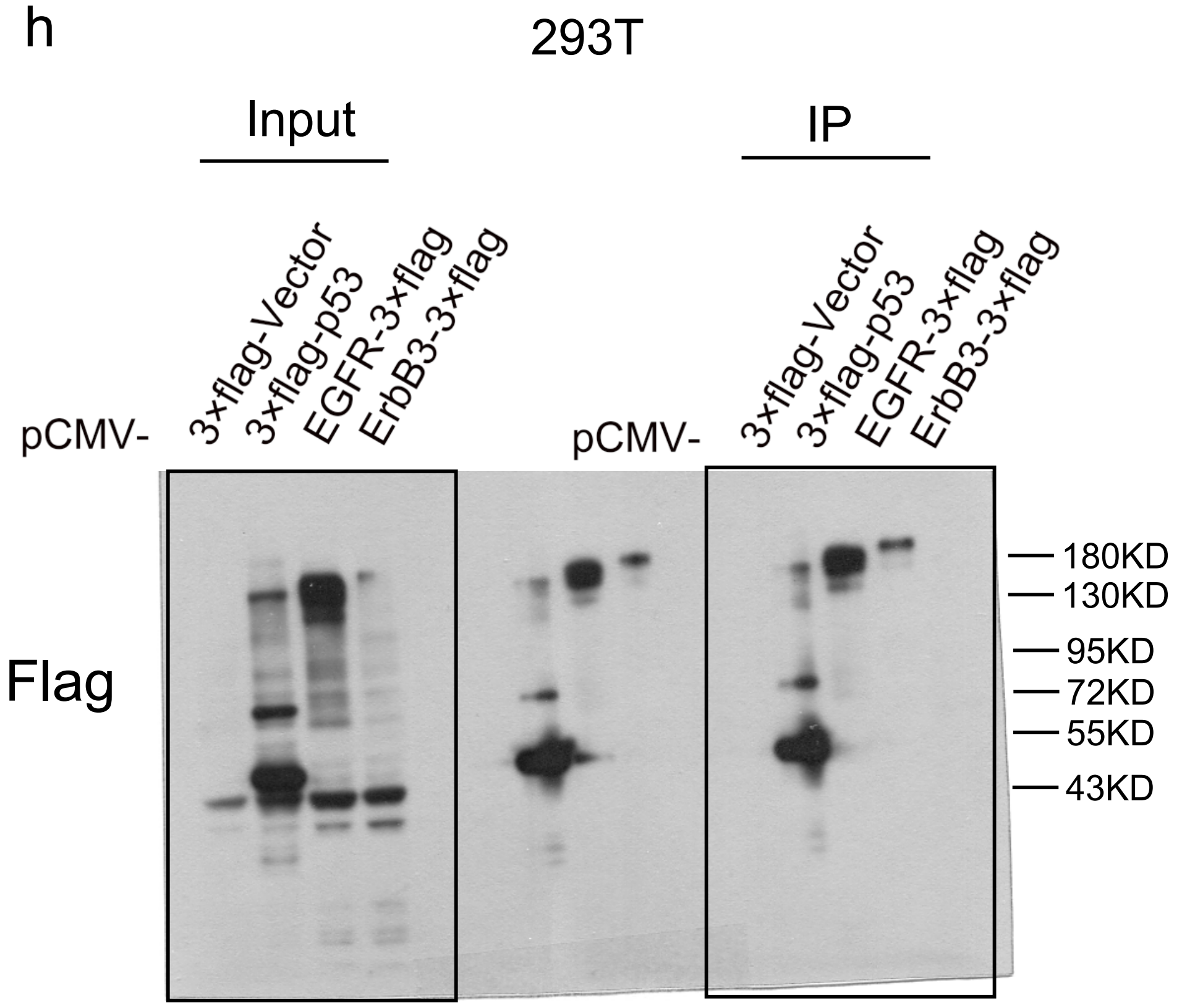

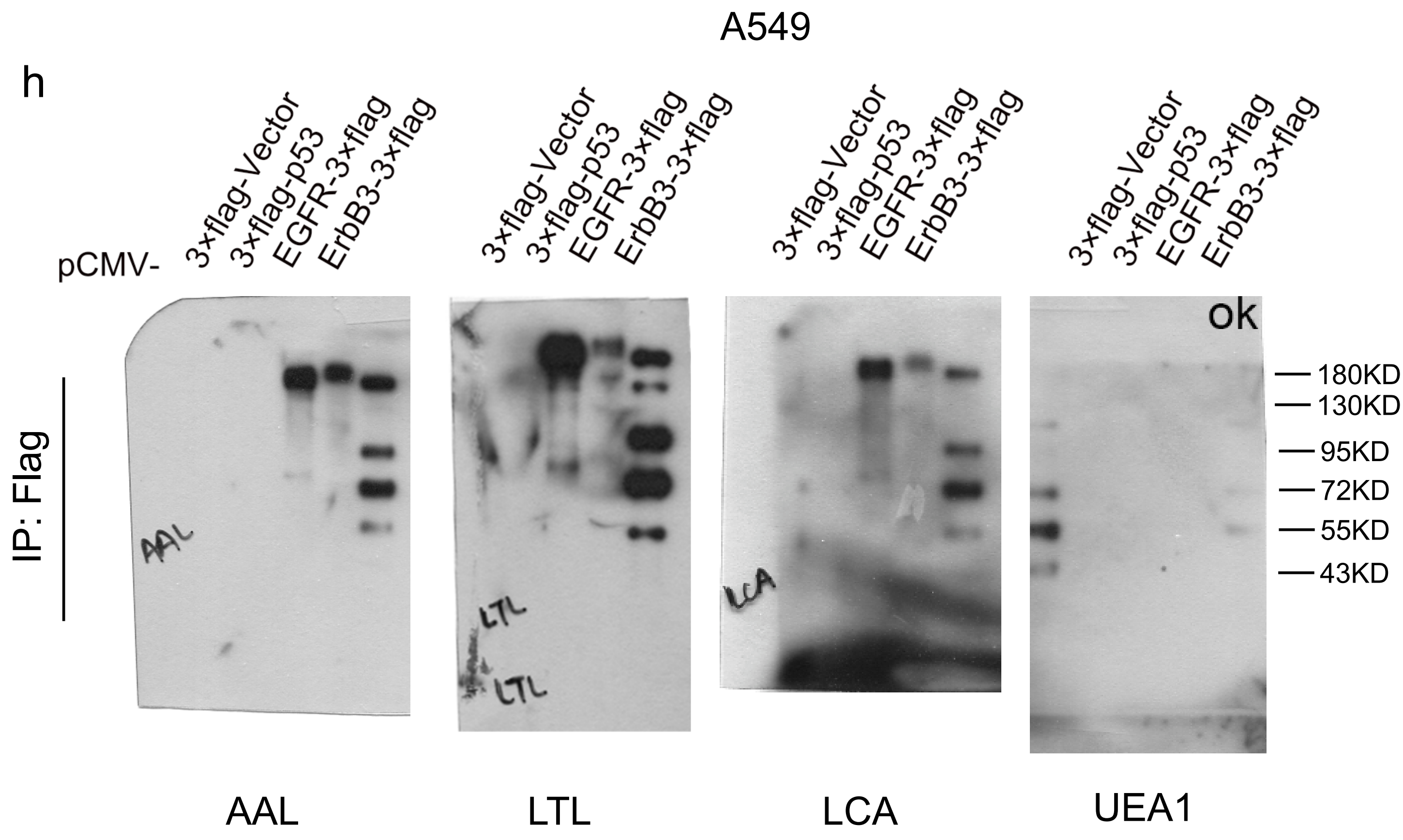

Extended Data Figure 5

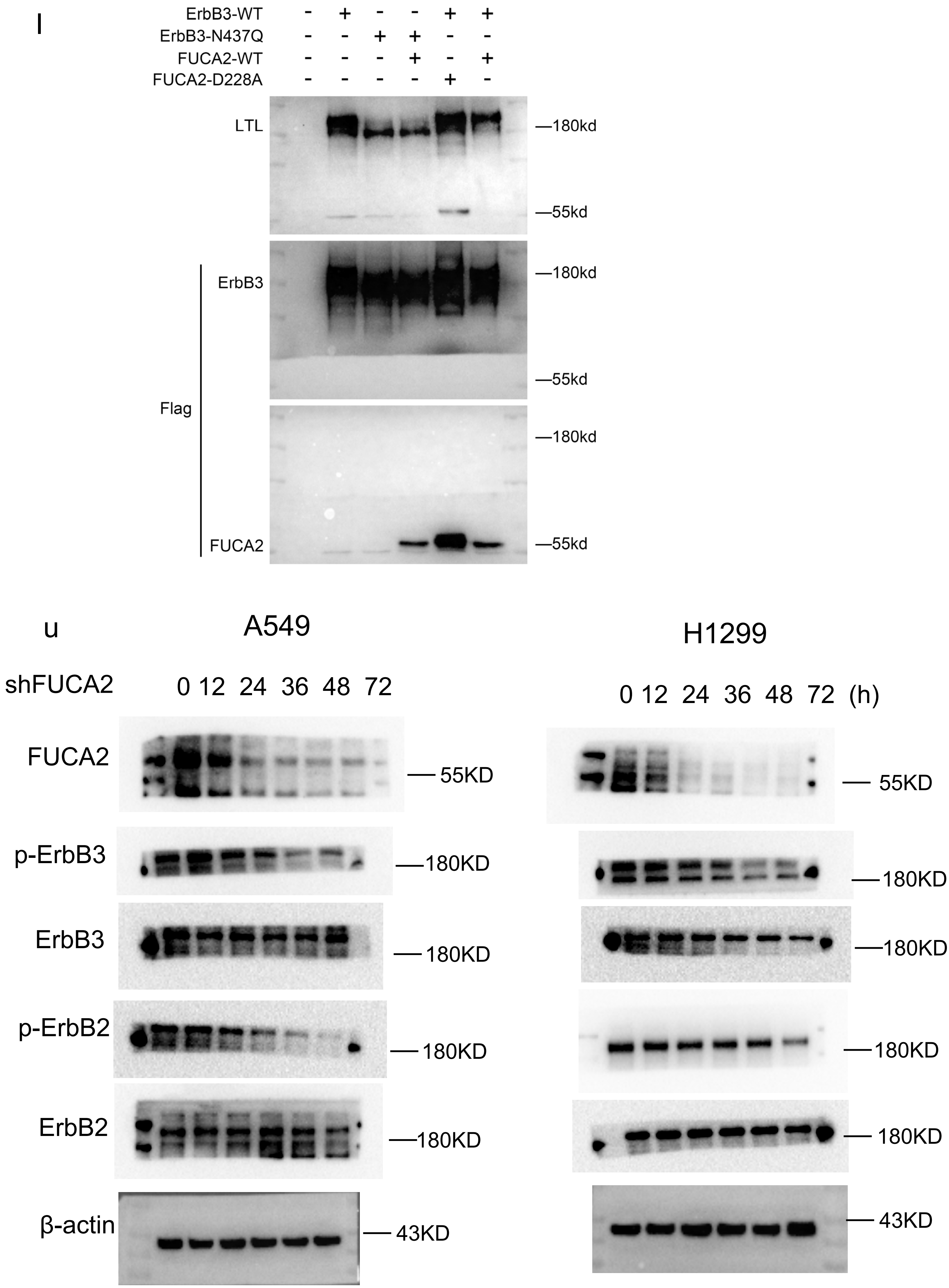

Figure 7

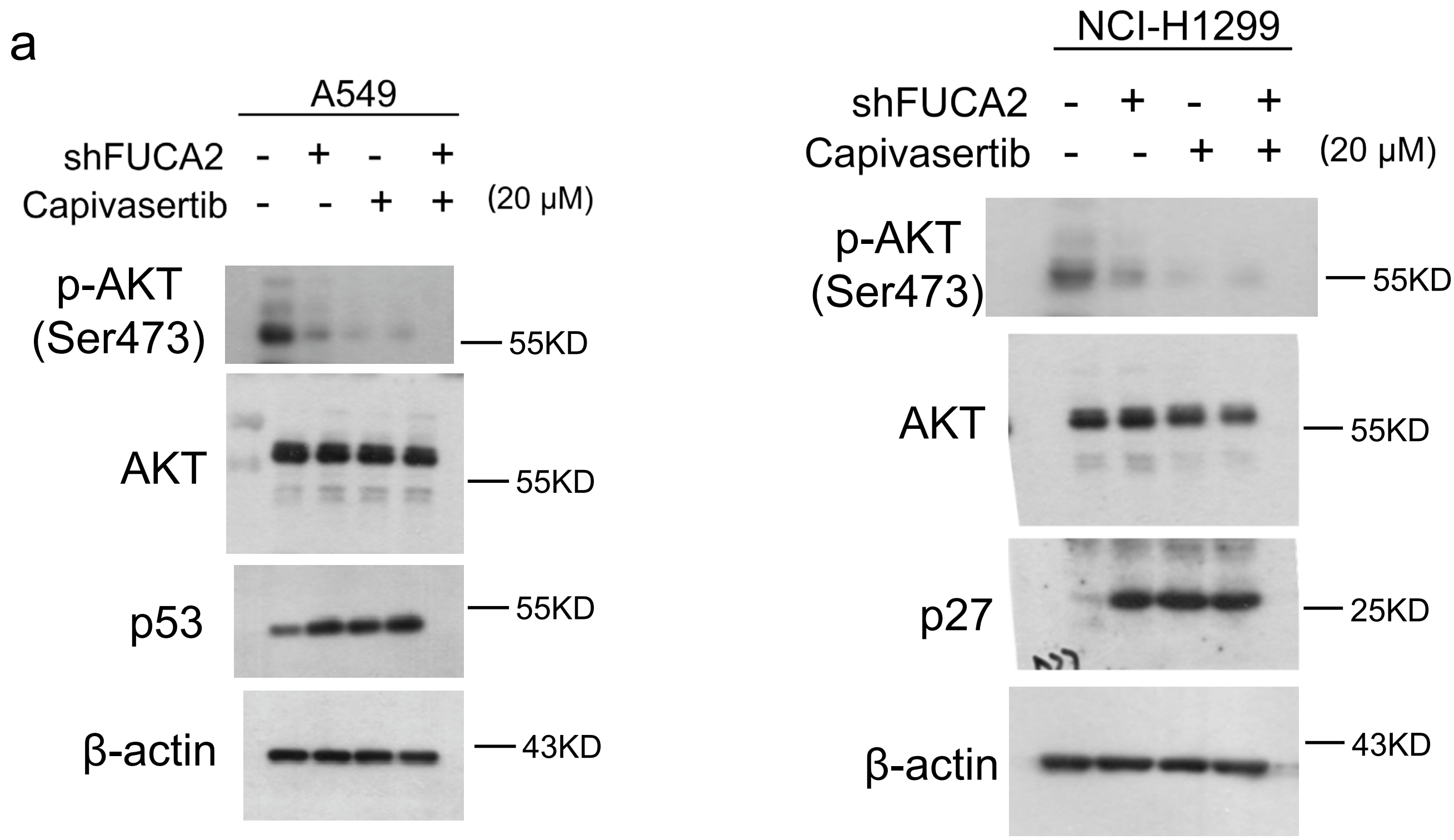

a
A549
shFUCA2 - + - +
Capivasertib - - + + (20 μM)
p-AKT (Ser473)
55KD
AKT
55KD
p53
55KD
β-actin
43KD
NCI-H1299
shFUCA2 - + - +
Capivasertib - - + + (20 μM)
p-AKT (Ser473)
55KD
AKT
55KD
p27
25KD
β-actin
43KD

Extended Data Figure 7

e

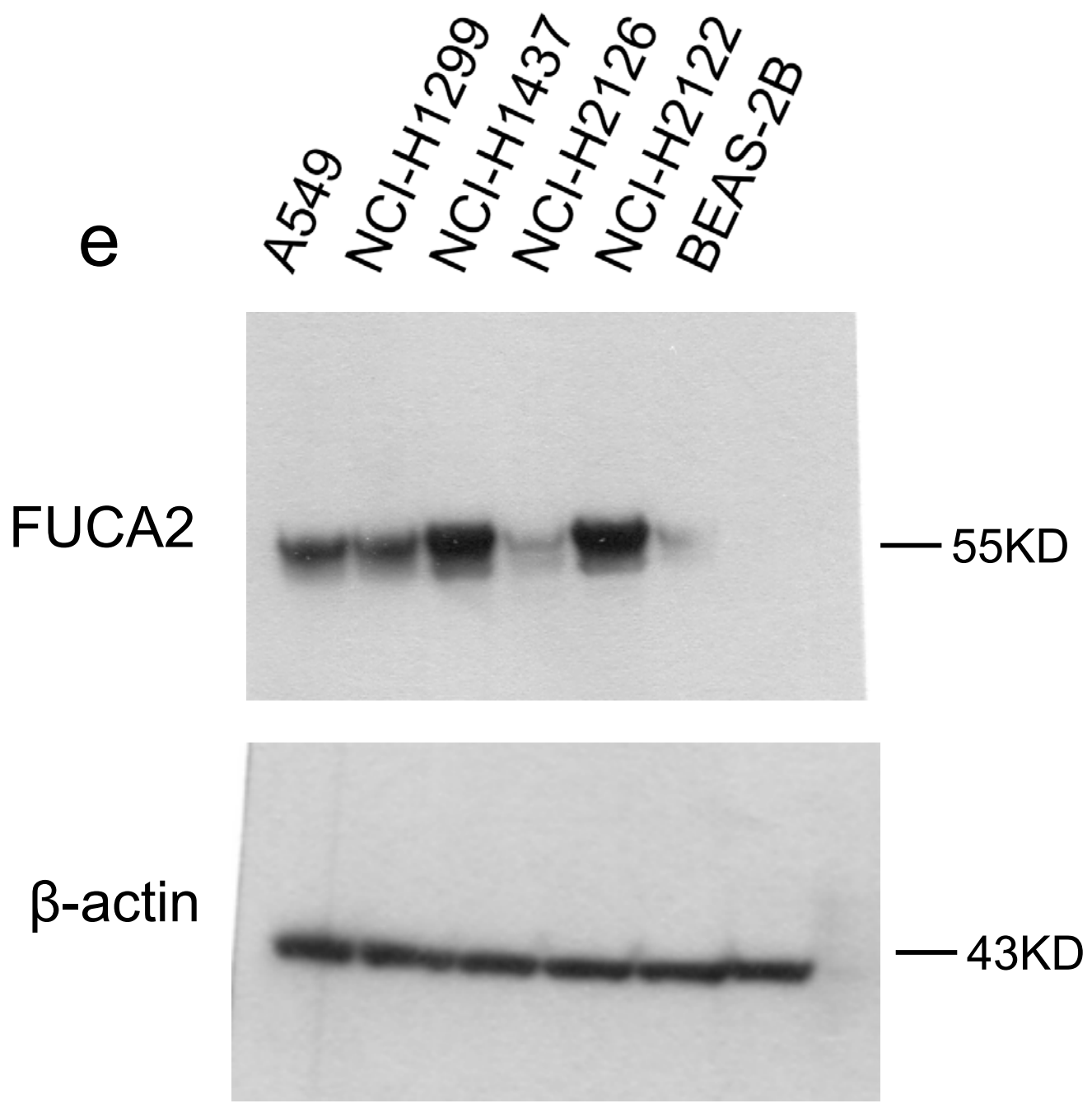

i

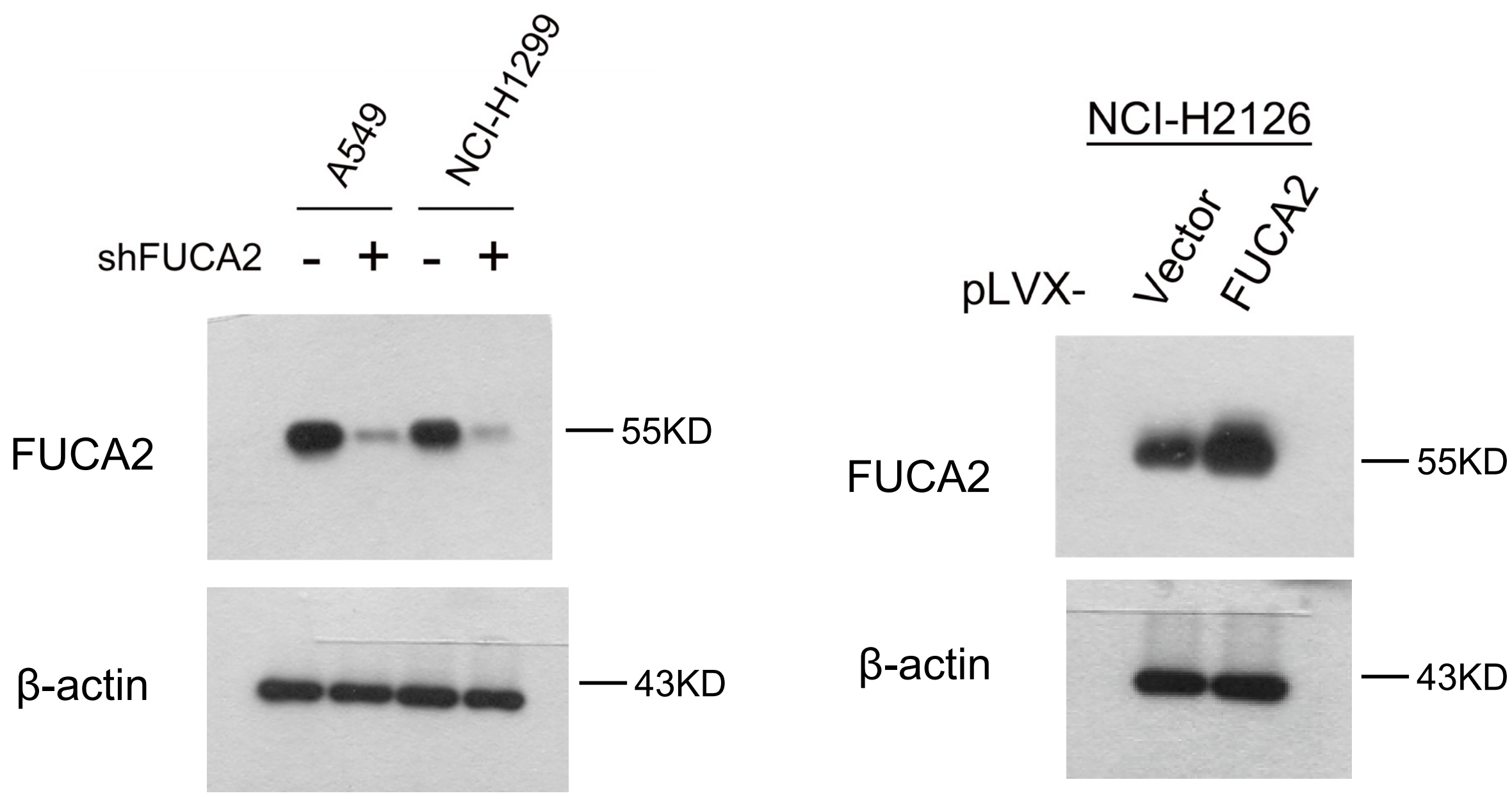

Extended Data Figure 8

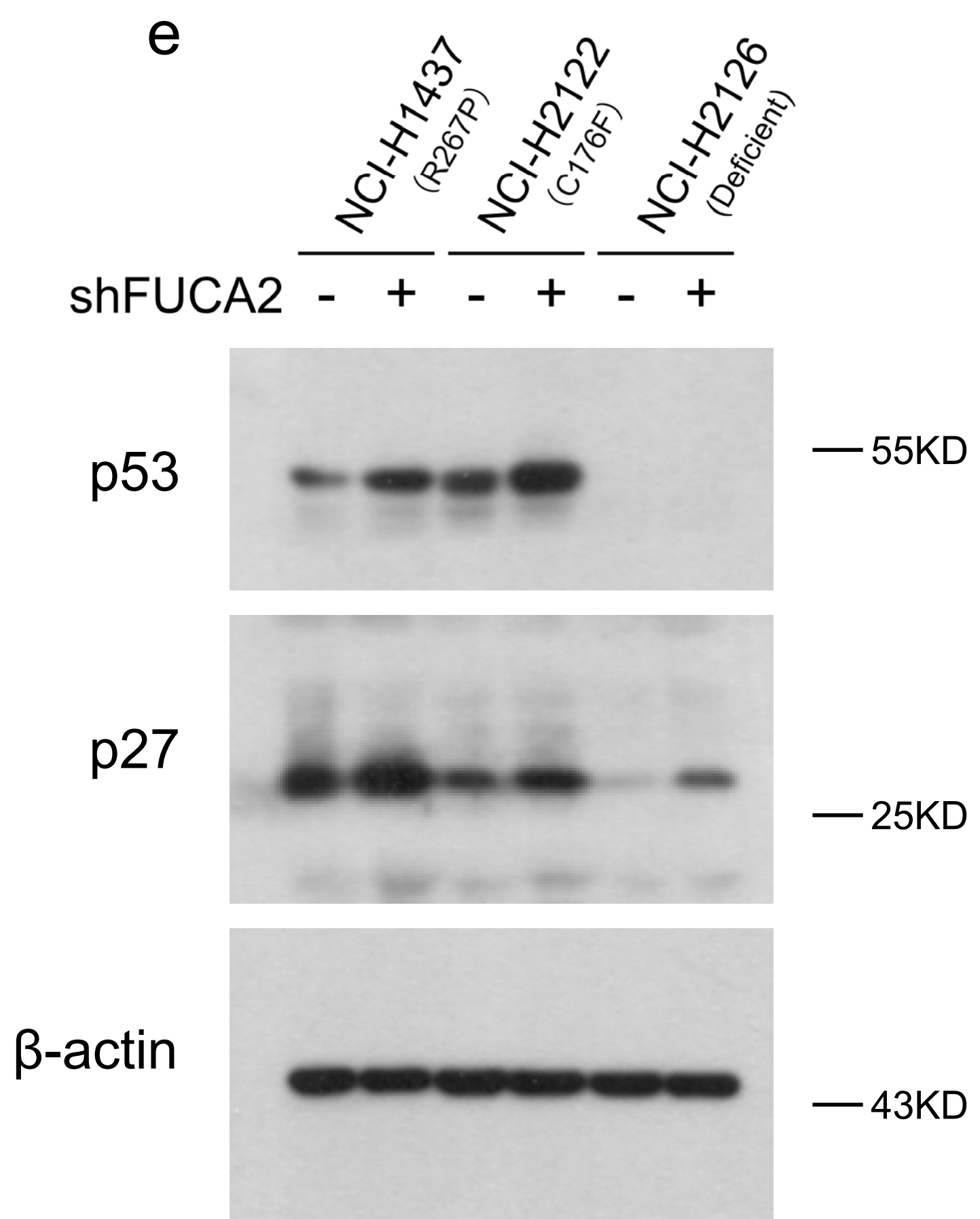

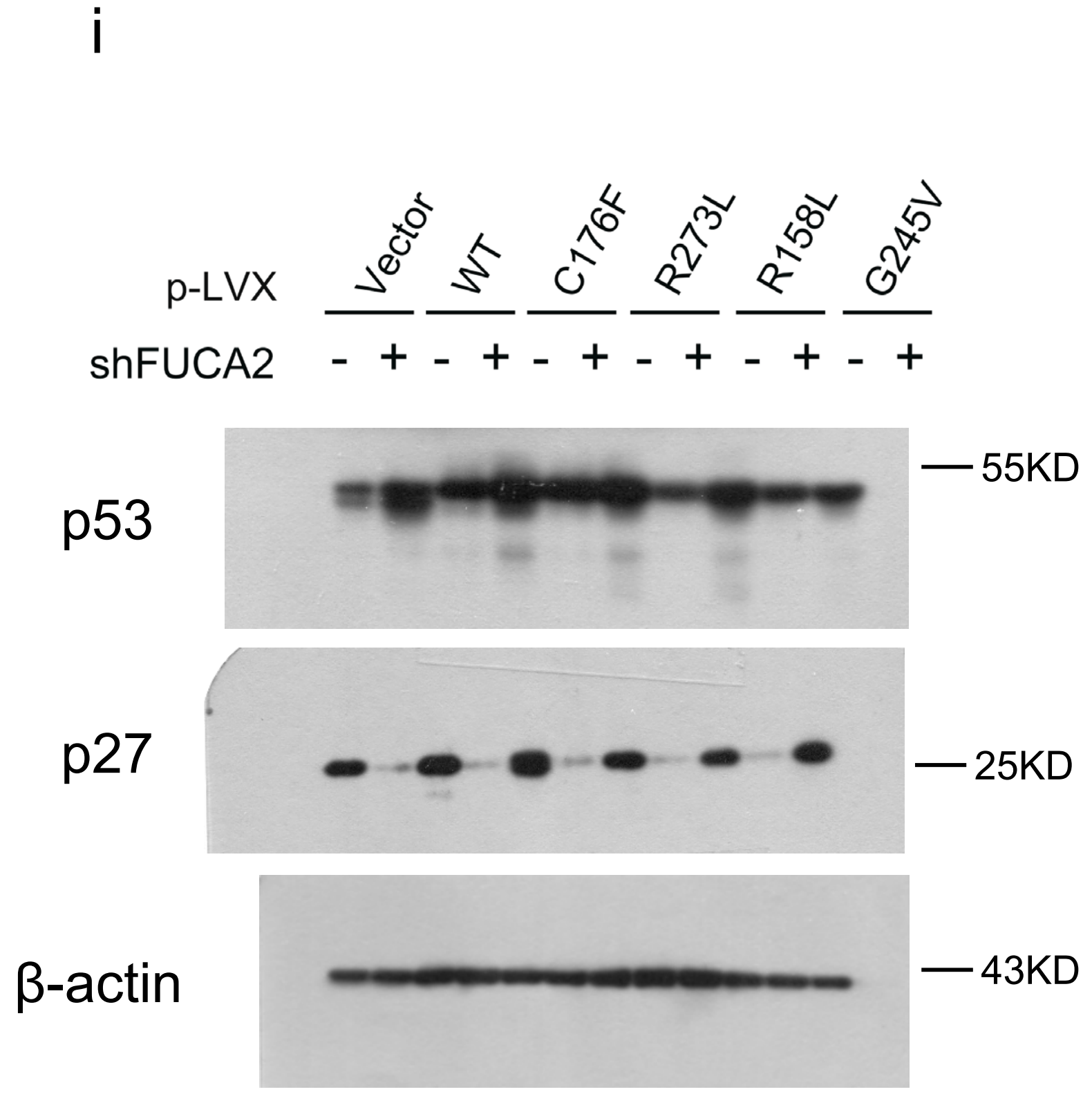

Extended Data Figure 9

f

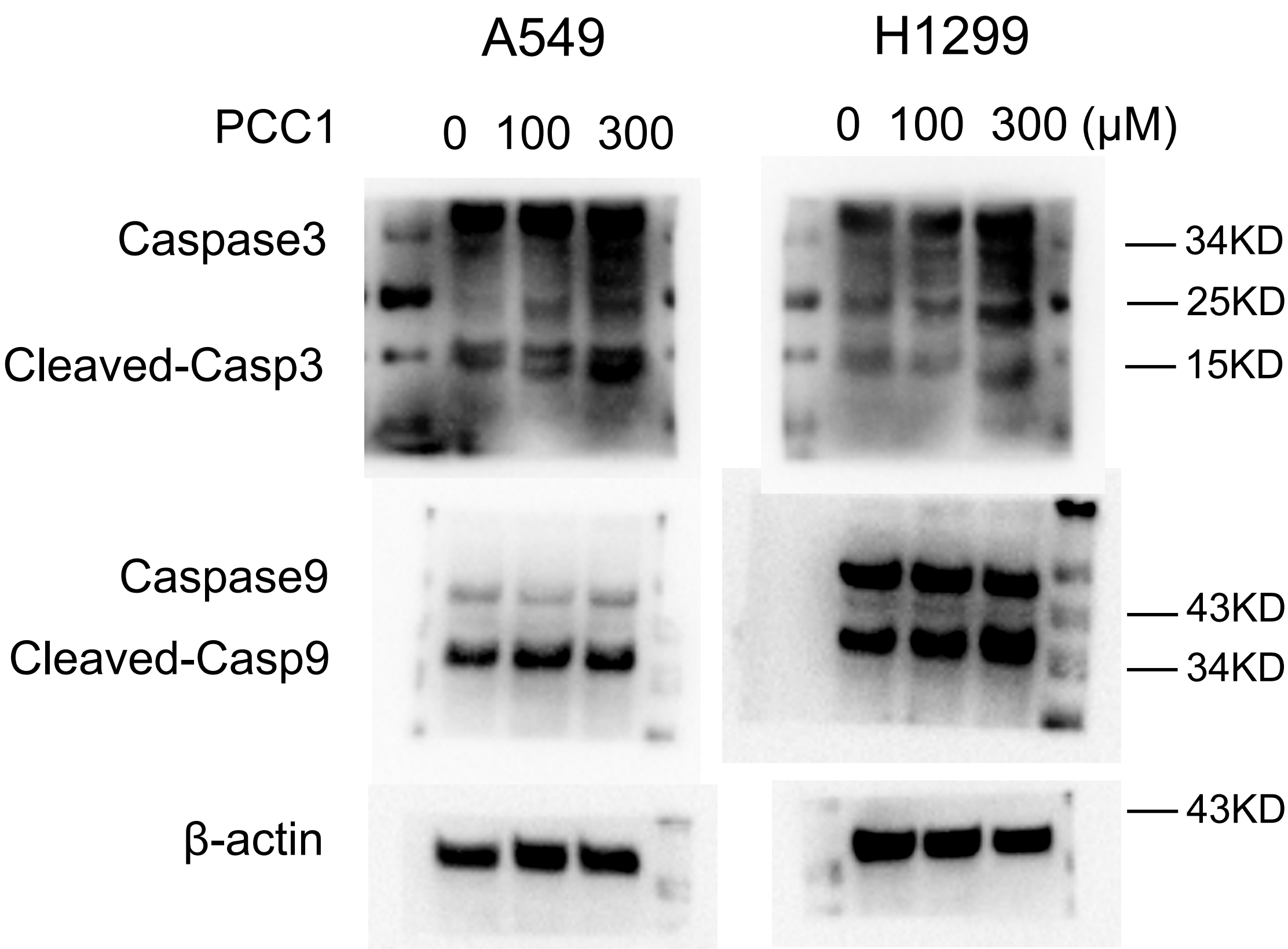

Extended Data Figure 10

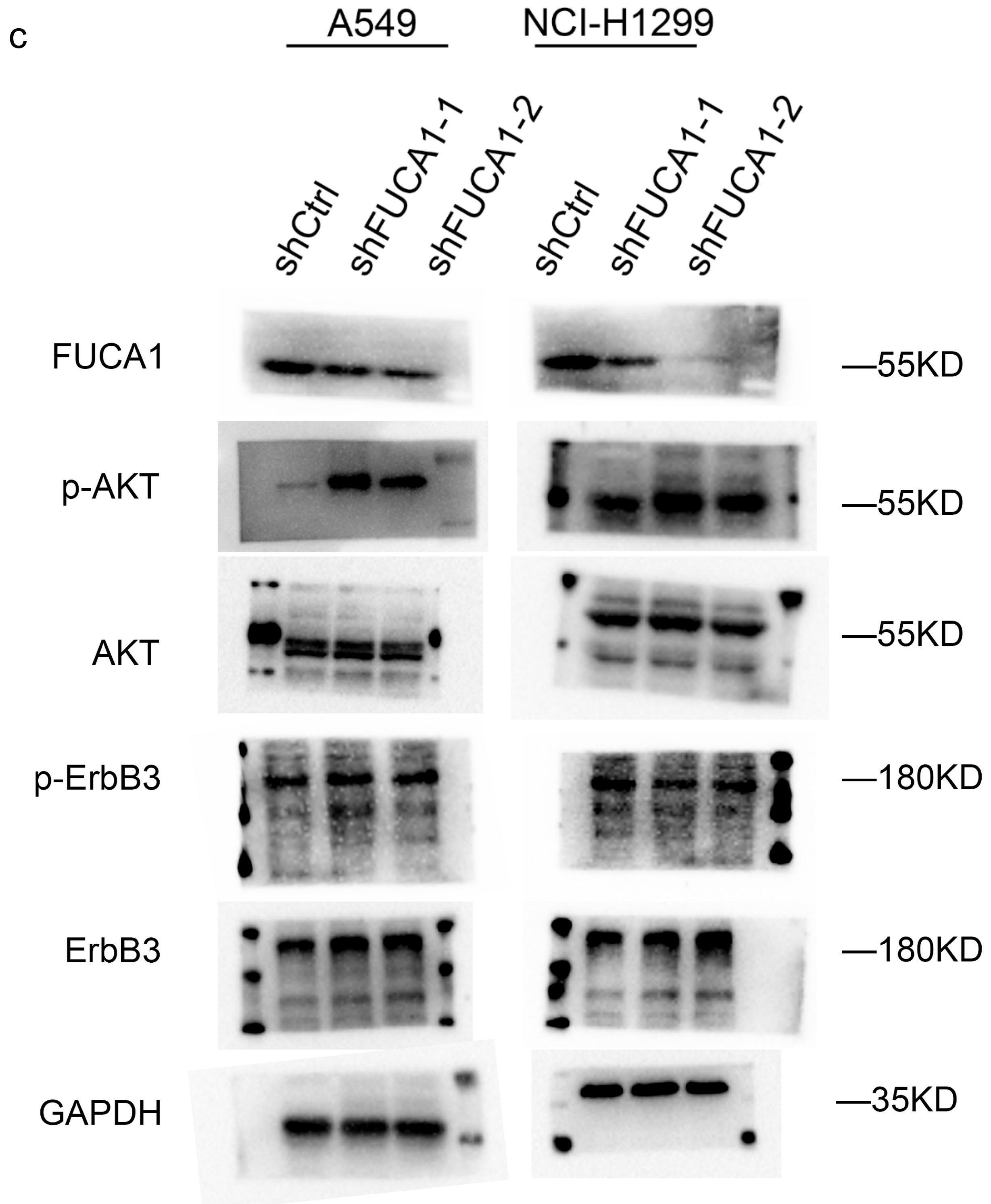

Supplement: Supplementary file 2 [file ADVS-13-e23667-s003.pdf]
