# Supplementary material for: FUCA2 Sustains AKT Signaling and Suppresses Senescence by Antagonizing FUT3‐Mediated ErbB3 Fucosylation in Lung Adenocarcinoma
Source: Adv Sci (Weinh). 2026 Jun 16:e23667. Online ahead of print. doi: 10.1002/advs.202523667 (PMC13335885; doi:10.1002/advs.202523667)
Supplement: Supplementary file 1 — Supporting File 1: advs75694‐sup‐0001‐SuppMat.pdf. [file ADVS-9999-e23667-s002.pdf]

Supporting Information for

FUCA2 Sustains AKT Signaling and Suppresses Senescence by Antagonizing FUT3-Mediated ErbB3 Fucosylation in Lung Adenocarcinoma

Lu Chen, Jintao Guo, Fei Li, Mingjie Gao, Runyang Li, Zhaozhang Huang, Yaolin Zheng, Chunyi Gao, Jihuan Hou, Qiang Yu, Bowen Zheng, Xuemei Chen, Wenqing Zhang, Xiaoting Hong, Yali Zheng, Daxuan Wang, Qiyuan Li, Tianhui Hu, Yan-yan Zhan

Including：

Supplementary Figures S1-10

Supplementary Table S1

Supplementary Material Tables S1-4

Supplementary Figures

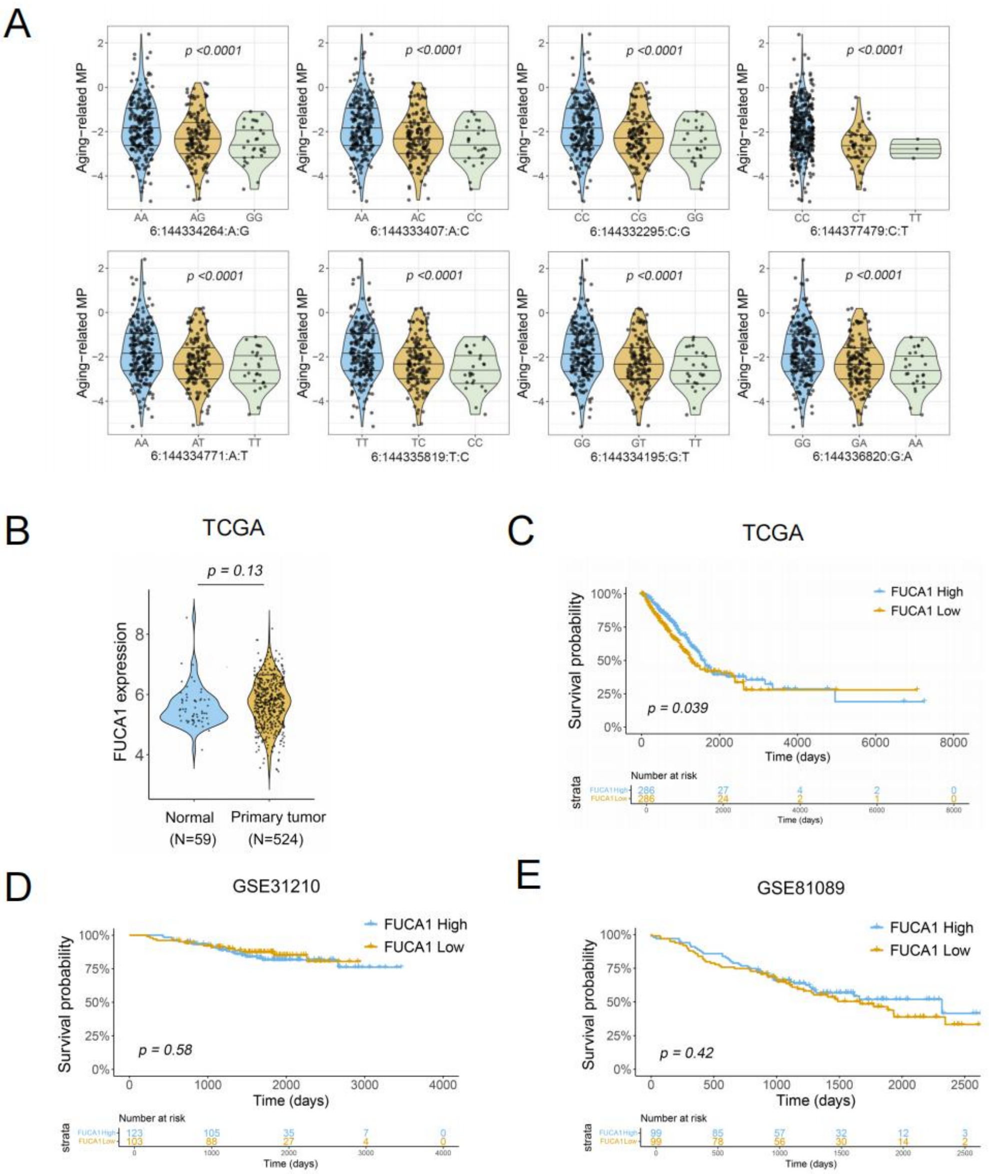

Supplementary Figure S1 | Bioinformatics screening identified FUCA2 as a novel potential driver gene inversely correlated with cellular senescence in LUAD.

(A) Allele frequency differences between FUCA2-related eight eQTLs and the senescence-associated somatic mutation gene set score. (B-C) TCGA LUAD dataset analyses of clinical relevance of FUCA1: No significant difference in FUCA1 expression between tumor and normal tissues (B, $p > 0.05$); FUCA1 expression shows no association with adverse prognosis (C, $p > 0.05$). (D, E) Analysis of GSE31210

and GSE81089 datasets from GEO database revealed no association between FUCA1 expression and overall survival in LUAD ($p > 0.05$).

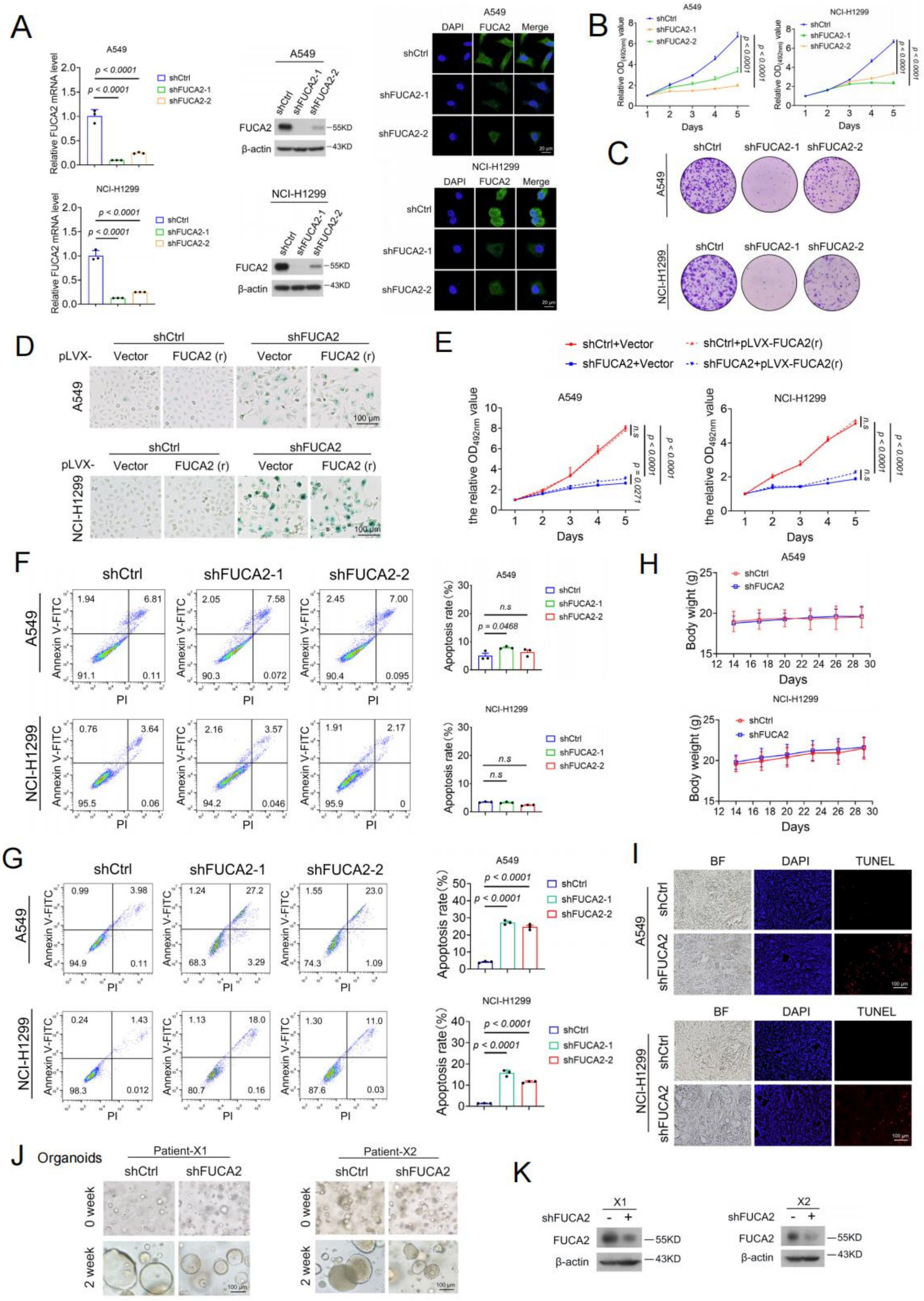

Supplementary Figure S2 | Knockdown of FUCA2 in A549 and H1299 cells inhibited cell

proliferation through inducing cell senescence *in vitro* and *in vivo*.

(A) FUCA2 knockdown efficiency in A549 and NCI-H1299 cells by real-time PCR (left panel), western blotting (middle panel), and immunofluorescence staining (right panel, scale bar: 20 μm). (B-C) Proliferation assays demonstrating growth inhibition upon FUCA2 depletion in A549 and NCI-H1299 cell lines: MTT assay (B) and colony formation assay (C). (D, E) Rescue experiments with FUCA2 overexpression (pLVX-FUCA2(r), a shRNA-resistant construct)) 4 days after FUCA2 knockdown: Reversal of senescence phenotype by SA-β-gal staining (D, scale bar: 100 μm); Restoration of proliferative capacity by MTT assay (E). (F, G) Apoptosis dynamics upon FUCA2 knockdown: Flow cytometry analysis of Annexin V/PI staining at 72h post-knockdown of FUCA2 (F, quantification of early apoptotic cells in Q3 quadrant); Delayed apoptosis following prolonged FUCA2 suppression (7 days), showing significant increase in apoptotic cells (G, quantification of early apoptotic cells in Q3 quadrant). (H, I) Subcutaneous xenograft models utilizing A549 and NCI-H1299 cells show reduced tumor growth upon FUCA2 knockdown: Mouse body weights throughout the experiment (H, n=6 per group); TUNEL staining (BF-bright field) demonstrates partial apoptosis induction in shFUCA2 tumors compared to controls (I, scale bar: 100 μm). (J) FUCA2 knockdown reduced spheroid size in LUAD organoids derived from two patients (X1 and X2). Scale bar: 100 μm. (K) FUCA2 knockdown efficiency in LUAD organoids derived from two patients (X1 and X2) by western blotting. Data represented the mean +/± SEM, and the *p* values were analyzed by one-way ANOVA (A, B, E-G).

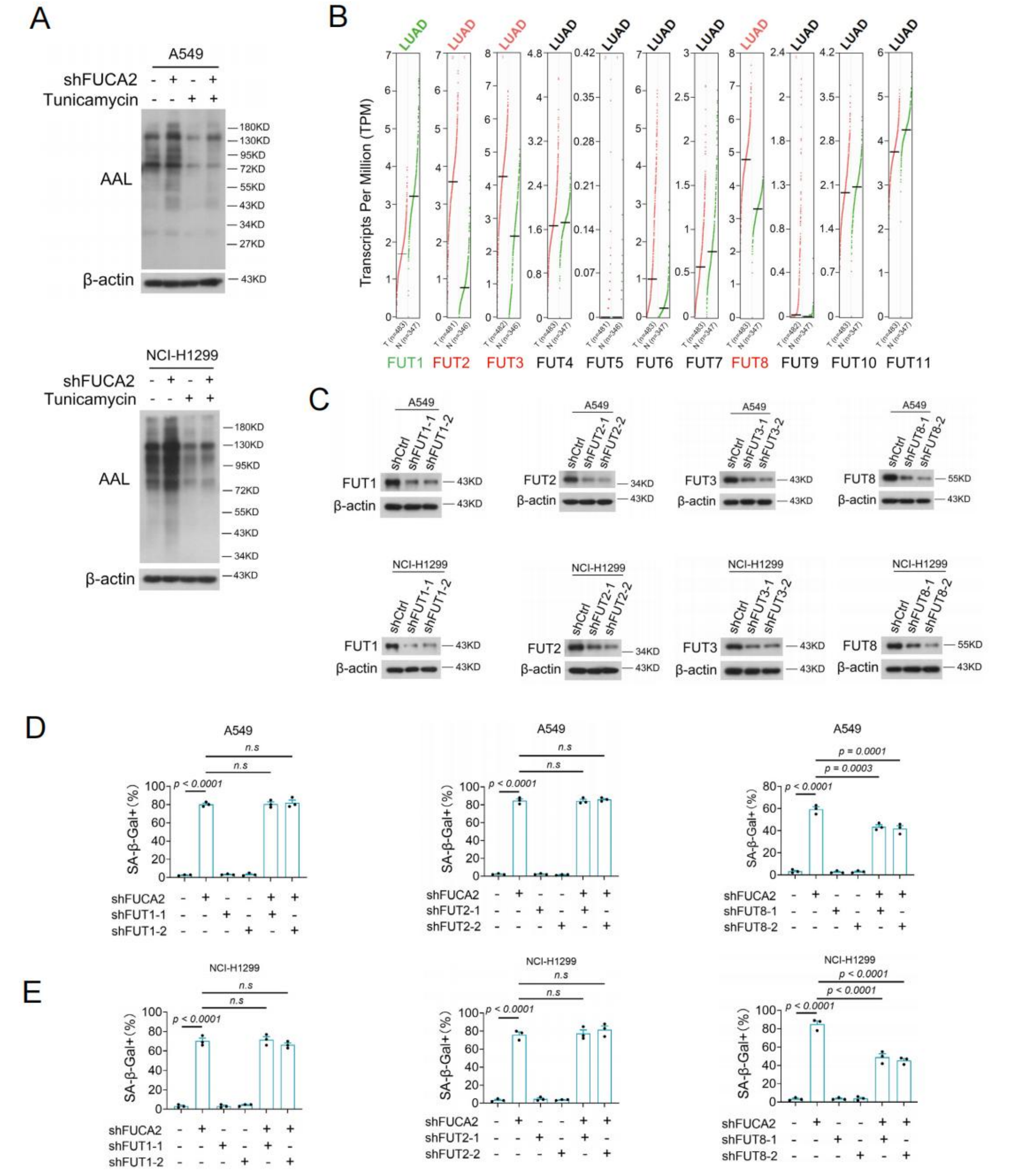

Supplementary Figure S3 | FUCA2's functional impact in lung adenocarcinoma required N-glycosylation modification mediated by FUT3.

(A) Lectin AAL assay measuring fucosylation modification levels in whole-cell lysates of A549/H1299 cells following Tunicamycin treatment (10 μM, 24 h). (B) Expression profiles of FUT1-11 in TCGA LUAD samples. (C) FUT1/FUT2/FUT3/FUT8 knockdown efficiency in A549 and NCI-H1299 cells were assessed by Western blotting. (D, E) Senescence-associated SA-β-Gal staining in

A549 cells (D) and NCI-H1299 cells (E) following combined knockdown of FUCA2 with either FUT1, FUT2, or FUT8. Data represented the mean + SEM, and the *p* values were analyzed by one-way ANOVA (D, E).

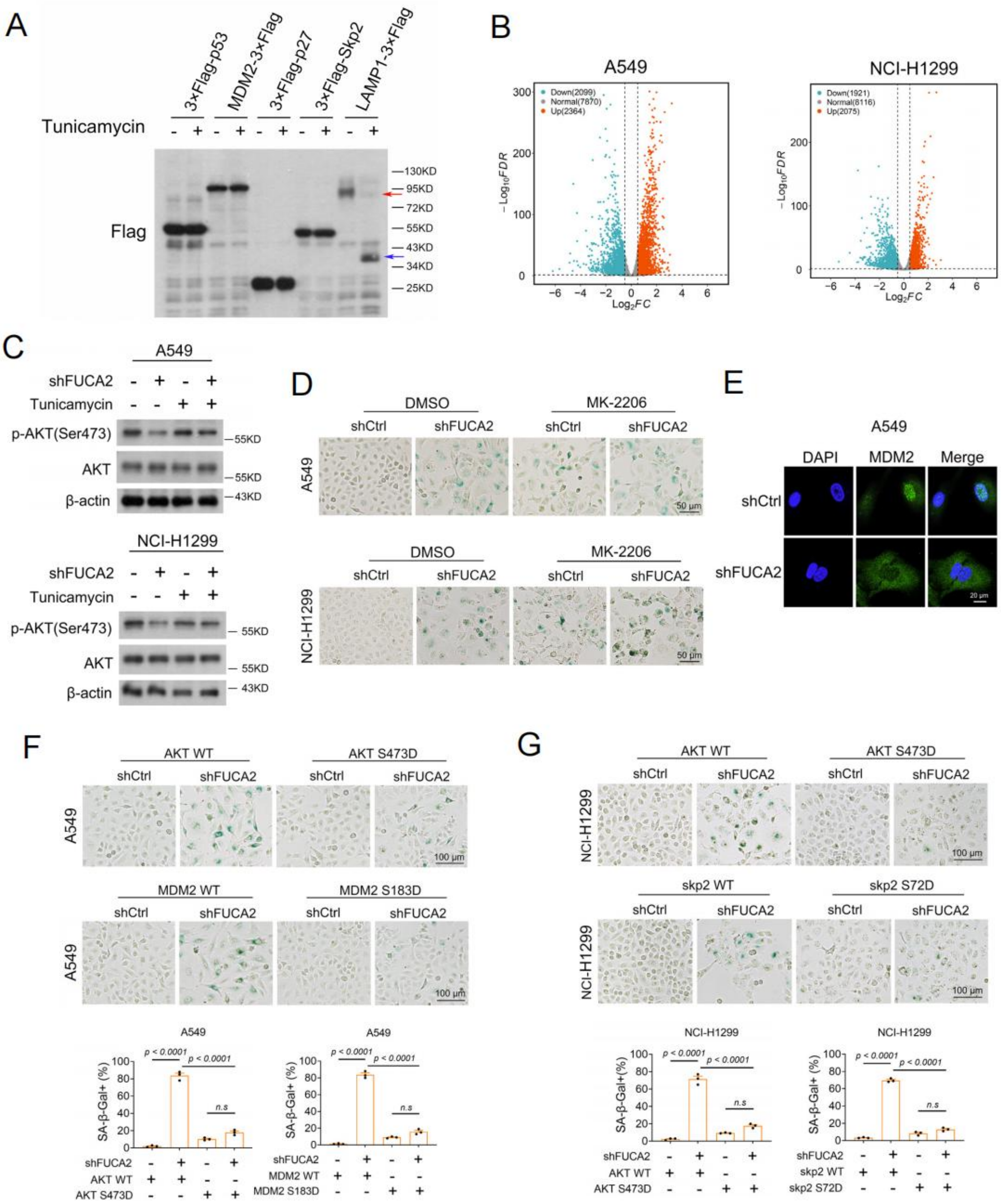

Supplementary Figure S4 | The PI3K/AKT signaling-mediated regulation of p53/p27 by FUCA2 required N-glycosylation (fucosylation).

(A) Western blotting analysis of N-glycosylation status following Tunicamycin

treatment (10 μM, 24 h), using LAMP2 as positive control (red arrow: glycosylated form; blue arrow: non-glycosylated form). (B) The volcano plot illustrated genes regulated by FUCA2 knockdown in A549 and NCI-H1299 cells. (C) Tunicamycin (10 μM, 24 h) blocked FUCA2 knockdown-induced reduction of p-AKT(Ser473) phosphorylation in both A549 and H1299 cell lines. (D) AKT inhibitor MK-2206 (20 μM, 3 days) blocked the effects of FUCA2 knockdown on cell senescence by SA-β-Gal staining (scale bar: 50 μm). (E) Immunofluorescence demonstrated altered MDM2 subcellular localization in FUCA2-knockdown A549 cells (scale bar: 20 μm). (F, G) SA-β-Gal staining confirmed that phospho-mimetic mutants (AKT S473D and MDM2 S183D), but not wild-type proteins, rescued FUCA2 knockdown-induced senescence in both A549 (F) and NCI-H1299 (G) cell lines (scale bars: 100 μm). Data represented the mean + SEM, and the *p* values were analyzed by one-way ANOVA (F, G).

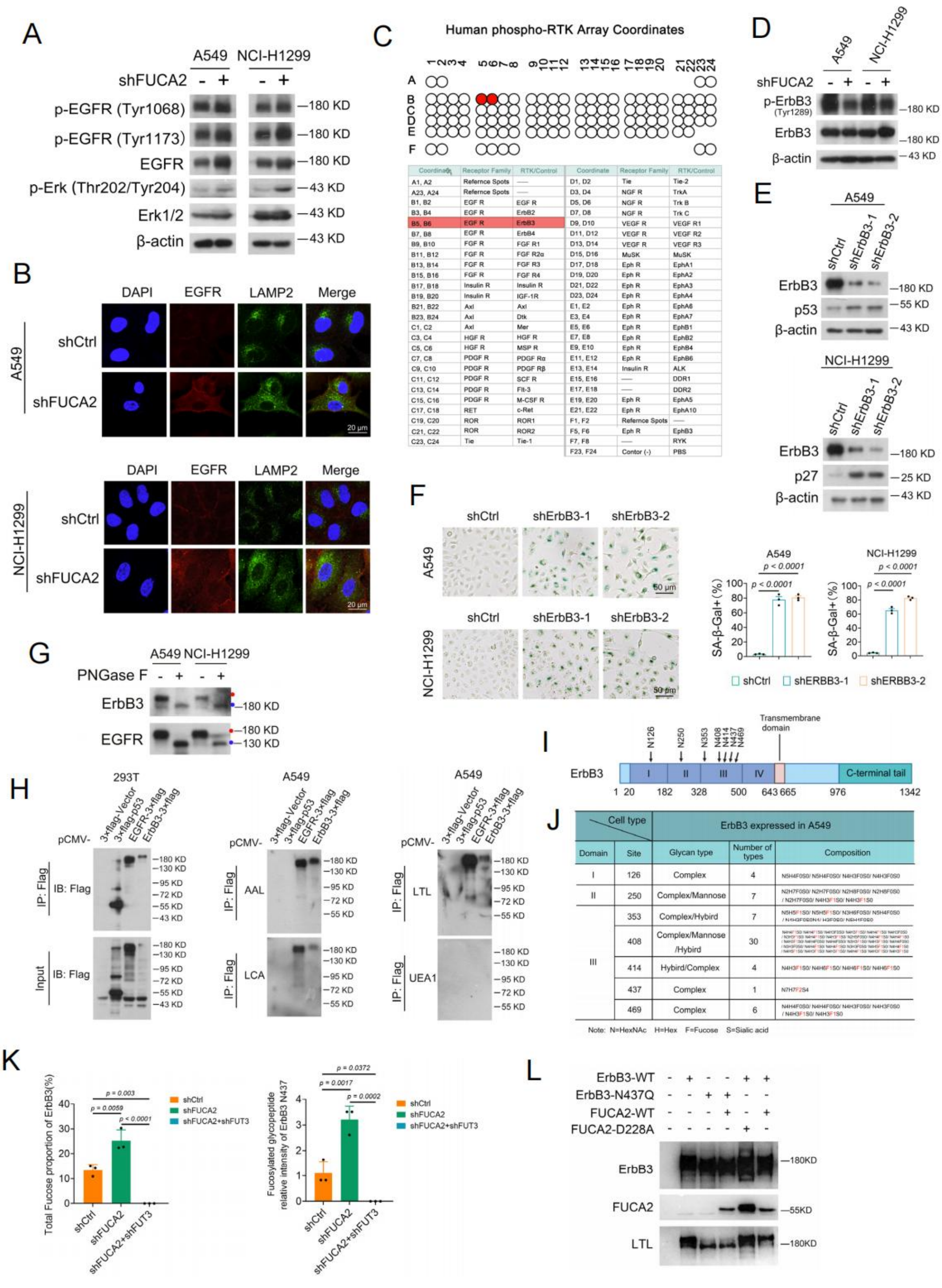

A
A549
NCI-H1299
shFUCA2
p-EGFR (Tyr1068)
p-EGFR (Tyr1173)
EGFR
p-Erk (Thr202/Tyr204)
Erk1/2
β-actin
180 KD
43 KD
B
DAPI
EGFR
LAMP2
Merge
shCtrl
shFUCA2
20 μm
C
Human phospho-RTK Array Coordinates
D
p-ErbB3 (Tyr1289)
ErbB3
E
shErbB3-1
shErbB3-2
p53
p27
55 KD
25 KD
F
50 μm
SA-β-Gal+(%)
p < 0.0001
shERBB3-1
shERBB3-2
G
PNGase F
130 KD
H
293T
pCMV-
3×flag-Vector
3×flag-p53
EGFR-3×flag
ErbB3-3×flag
IP: Flag
IB: Flag
Input
AAL
LCA
LTL
UEA1
I
Transmembrane domain
C-terminal tail
J
Cell type
ErbB3 expressed in A549
Domain
Site
Glycan type
Number of types
Composition
Note: N=HexNAc H=Hex F=Fucose S=Sialic acid
K
Total Fucose proportion of ErbB3(%)
Fucosylated glycopeptide relative intensity of ErbB3 N437
shFUCA2+shFUT3
L
ErbB3-WT
ErbB3-N437Q
FUCA2-WT
FUCA2-D228A
FUCA2

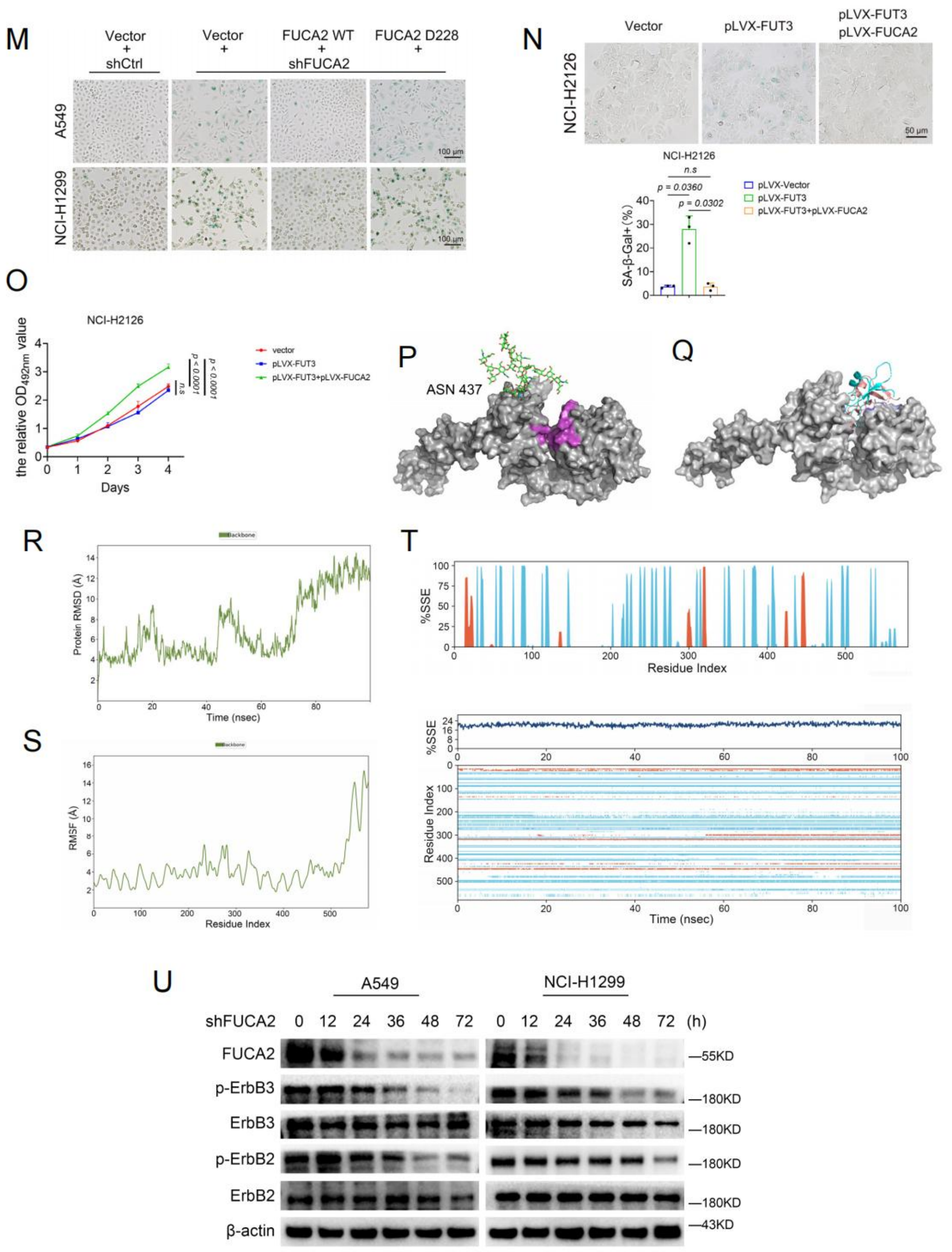

Supplementary Figure S5 | FUCA2 knockdown activated EGFR signaling while impairing ErbB3 function through N-glycosylation modulation.

(A) Western blots showing elevated EGFR, p-EGFR and p-Erk in FUCA2-knockdown A549/NCI-H1299 cells. (B) Immunofluorescence revealed enhanced EGFR membrane localization (red) with lysosomal marker LAMP2 (green) and DAPI (blue) (scale bars: 20 μm). (C) Detailed information of Phospho-RTK array,

with ErbB3 position highlighted (red). (D) Reduced ErbB3 phosphorylation post-FUCA2 knockdown. (E, F) ErbB3 knockdown increase p53/p27 protein levels (E) and induced senescence (F). (G) PNGase F treatment confirmed N-glycosylation on ErbB3 in A549 and NCI-H1299 cells (red: glycosylated; blue: deglycosylated). (H) Lectin profiling showed abundant α-1,3/α-1,6- (but minimal α-1,2-) fucosylation on ErbB3. (I) Schematic of ErbB3 protein domains. (J) UPLC-MS/MS-identified N-glycosylation sites/types on ErbB3. (K) Quantitative mass spectrometry showing that FUCA2 knockdown increased α-1,3-fucosylation at Asn437 by approximately 2-fold, which was abolished by additional FUT3 knockdown. (L) *In vitro* defucosylation assay confirming that wild-type FUCA2, but not D228A, specifically targeted Asn437. (M) Rescue experiment demonstrating that FUCA2(r) rescued senescence whereas FUCA2(r, D228A) did not. (N, O) FUT3 overexpression alone induced senescence and mildly suppressed growth, whereas co-overexpression with FUCA2 abrogated senescence and promoted growth. (P-T) Molecular docking and MD simulation analyses of ErbB3 glycosylation. (P, Q) MD simulations of ErbB3 characterizing the steric hindrance of N-glycosylation (P) and redocking benchmark validation of the unmodified ErbB3-NRG1b binding mode (Q). (P) Glycosylated ErbB3 was rendered as a gray surface, with the NRG1b-binding pocket highlighted in purple and the Asn437-linked N-glycan shown as a cyan ball-and-stick model. The glycan projected directly over the binding interface, creating profound steric occlusion that physically restricts NRG1b access. Crucially, HADDOCK3 docking simulations further corroborated this spatial blockade, failing to yield any viable binding poses for NRG1b at the modified pocket. This steric restriction thereby precluded ligand engagement and subsequent ErbB3-ErbB2 heterodimerization. (Q) The unmodified ErbB3 receptor was depicted as a gray surface. The native

crystallographic pose of the NRG1b ligand (PDB ID: 7MN8) was represented by a salmon cartoon, while the optimal docking conformation predicted via the HADDOCK3 platform was shown as a cyan cartoon. The two conformations exhibited exceptional three-dimensional spatial concordance within the binding pocket. (R-T) Structural stability of glycosylated ErbB3 during 100 ns MD simulation. (R) Backbone RMSD trajectory. (S) RMSF profile showing residue-level flexibility. (T) SSE analysis: SSE frequency per residue (orange, α-helix; blue, β-sheet; top), total SSE percentage over time (~20-24%; middle), and time-dependent SSE evolution per residue (bottom). (U) Time-course analysis of ErbB3 and ErbB2 phosphorylation upon FUCA2 knockdown. Data represented the mean + SEM, and the *p* values were analyzed by one-way ANOVA (F, K, N, O).

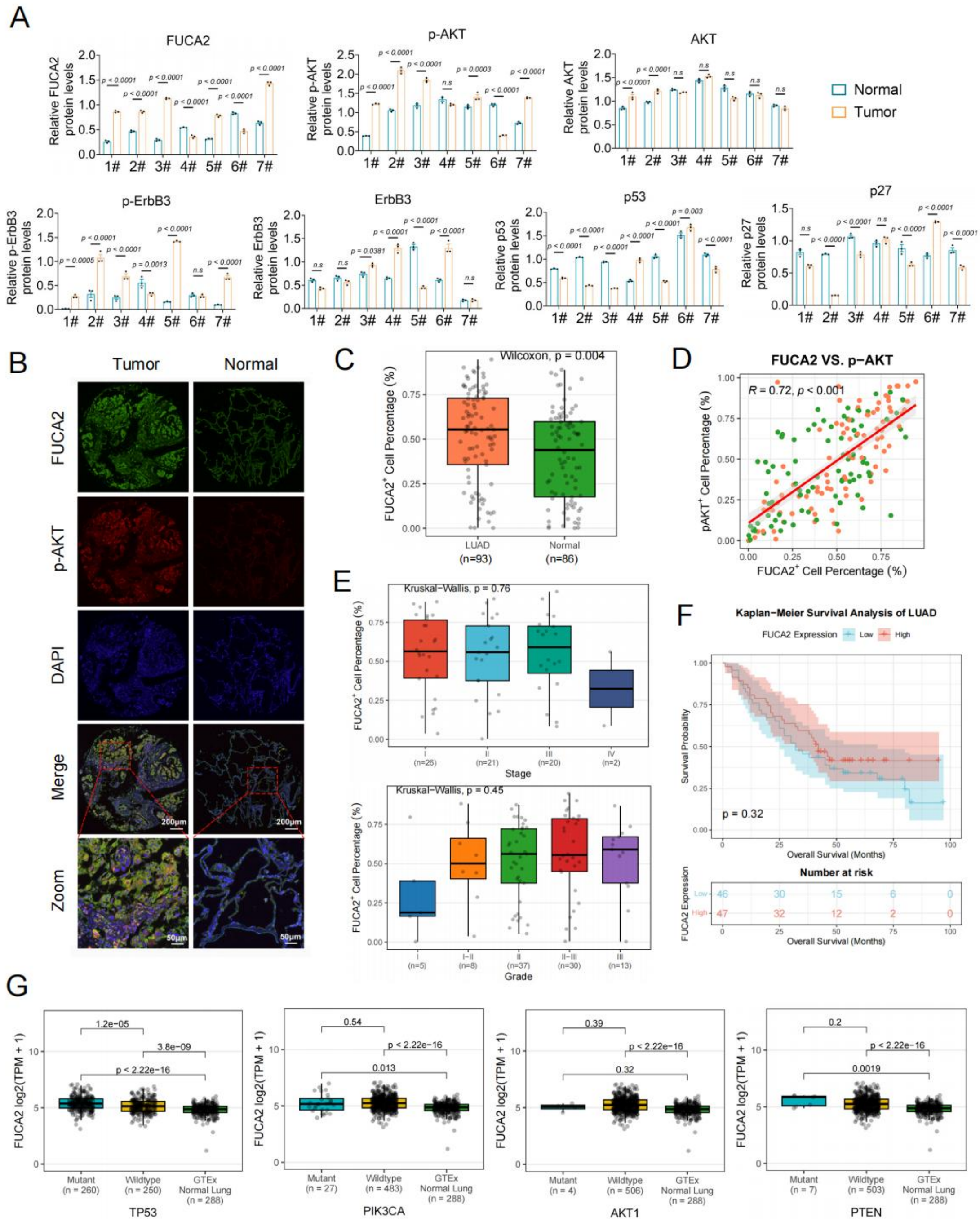

Supplementary Figure S6 | Clinical validation of FUCA2 as a biomarker in LUAD.

(A) Quantitative analysis of FUCA2, p-AKT, AKT, p-ErbB3, ErbB3, p53, and p27 in clinical LUAD samples in Figure 6I. (B-F) Multiplex fluorescence staining on a LUAD tissue microarray (94 LUAD, 86 adjacent normal). Representative staining images of FUCA2 and p-AKT in one matched pair of LUAD and adjacent normal

tissue (B, scale bars: 200 μm for main panels, 50 μm for insets). FUCA2 expression was elevated in LUAD (C) and positively correlated with p-AKT (D). Correlations of FUCA2 expression with tumor stage and grade (E) and survival (F). Among the total cohort (n = 93, as one sample detached during staining), patients with missing clinical stage (n = 24) were excluded from this stratified analysis to ensure statistical validity. (G) TCGA analysis revealing that FUCA2-high tumors were prevalent across LUAD subtypes irrespective of TP53/PIK3CA/AKT1/PTEN mutation status. Data were presented as mean ± SEM for (A) and as boxplots for (C, E, G). Statistical significance was determined by unpaired two-tailed Student's *t*-test (A), Wilcoxon rank-sum test (C, G), Spearman correlation analysis (D), Kruskal-Wallis test (E), and log-rank test (F).

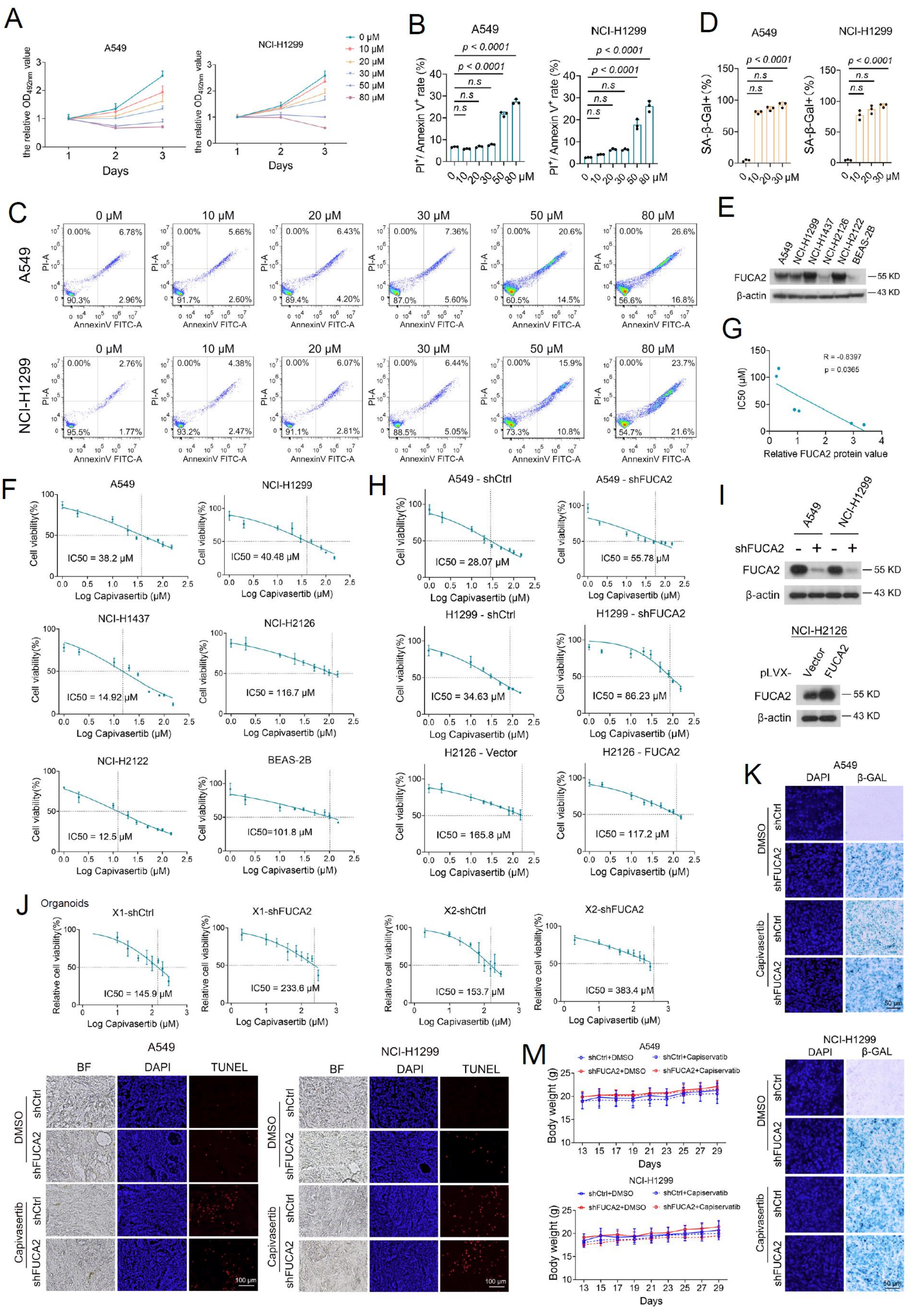

Supplementary Figure S7 | Concentration-dependent effects of Capivasertib and the correlation of its anti-LUAD efficacy with FUCA2 expression.

(A-D) Dose-response analyses for Capivasertib in A549 and NCI-H1299 cells. MTT proliferation assay (0-80 μM, 48 h) (A), Annexin V/PI apoptosis quantification (0-80

μM, 48 h) (B, C), and SA-β-Gal-positive cell percentages (D, ≤30 μM, 48 h) were shown. (E) Expression of FUCA2 in five LUAD cell lines and normal lung epithelial cell line BEAS-2B were detected by western blotting. (F) Capivasertib IC50 values (48 h treatment) across the above cell lines were analyzed by MTT assay and calculated using four-parameter logistic nonlinear regression. (G) Negative correlation between Capivasertib IC50 values (E) and FUCA2 levels (F). (H) FUCA2 knockdown (A549/NCI-H1299 cells) increased while overexpression (NCI-H2126) decreased Capivasertib IC50 values by MTT assay. (I) Knockdown and overexpression effect of FUCA2 in A549/NCI-H1299 cells and NCI-H2126 cells, respectively, were detected by western blotting. (J) Capivasertib dose-response analyses (48 h treatment) showing right-shifted IC50 curves upon FUCA2 knockdown in patient X1/X2-derived organoids by ATP viability assay. (K, L) SA-β-gal staining (demonstrating senescence; scale bar: 50 μm) and TUNEL staining (demonstrating apoptosis; BF-bright field; scale bar: 100 μm) of tumor sections in Figure 7d-j. m, Nude mouse body weights during 21-day Capivasertib treatment (50 mg/kg/day). Data represented the mean + or ± SEM, and the *p* values were analyzed by one-way ANOVA (B, D).

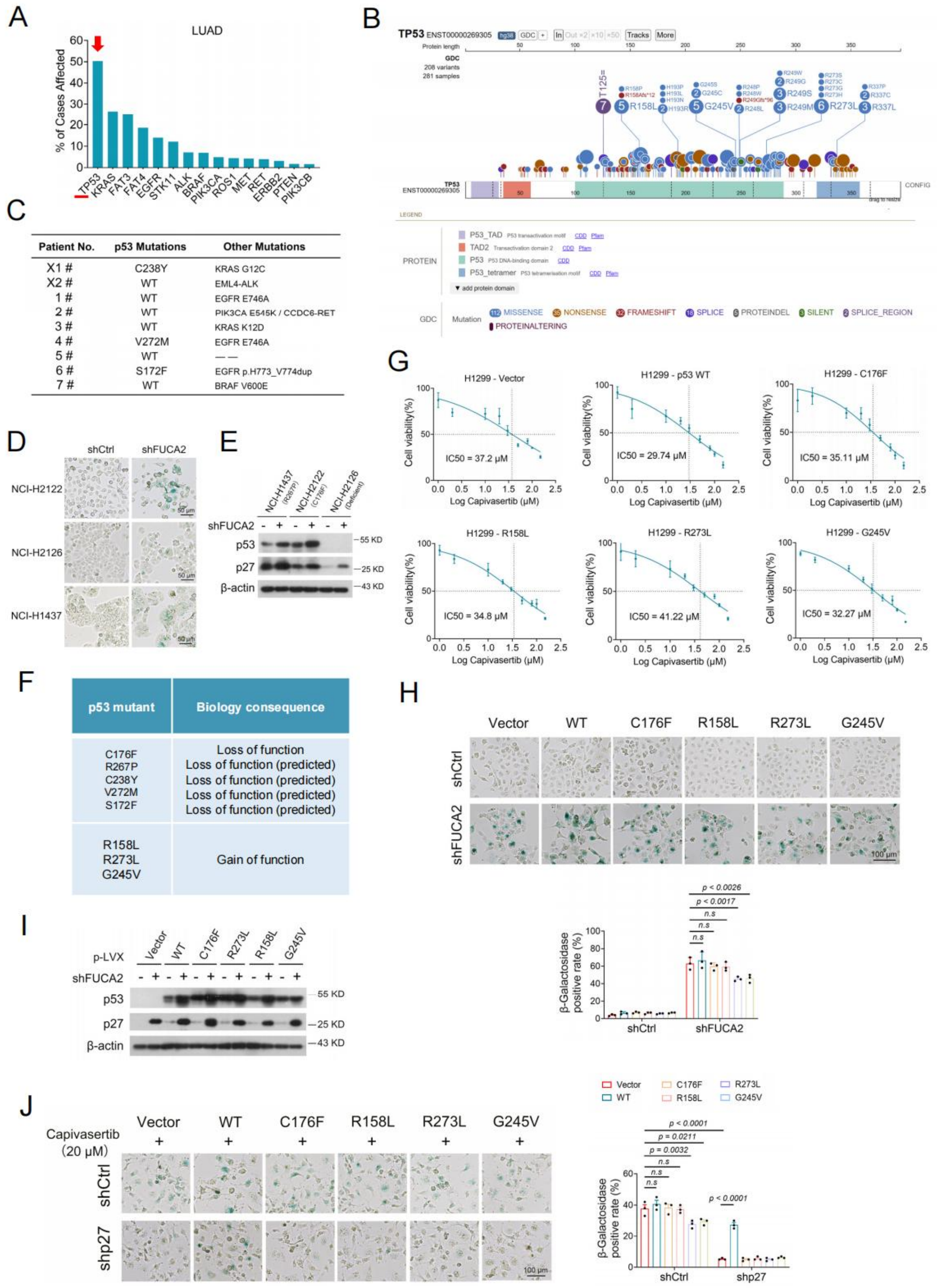

Supplementary Figure S8 | Genetic landscape of TP53 in LUAD and its functional interplay with FUCA2-mediated senescence.

(A, B) Genomic Data Commons (GDC) database analysis of LUAD patients revealed TP53 gene mutation frequency (A) with detailed mutated types (B). (C) Spectrum of

TP53 and other driver mutations in clinical LUAD samples. (D, E) Effects of FUCA2 knockdown on cellular senescence and p53/p27 protein levels in NCI-H2122/H2126/H1437 cells by SA-β-Gal staining (D) and western blotting (E). (F) Functional characterization of different TP53 mutations. (G) Capivasertib IC50 values in NCI-H1299 cells overexpressing wild-type versus mutant p53. (H) SA-β-Gal staining showed FUCA2 knockdown-induced senescence in p53 wild-type/mutant-overexpressing NCI-H1299 cells. (I) FUCA2 knockdown effects on p53/p27 levels in p53 wild-type/mutant-overexpressing NCI-H1299 cells. (J) Capivasertib-induced senescence requires p53 and p27 signaling in p53-wild-type and -mutated LUAD cells, respectively. Data represented the mean + or ± SEM, and the *p* values were analyzed by one-way ANOVA (I, J).

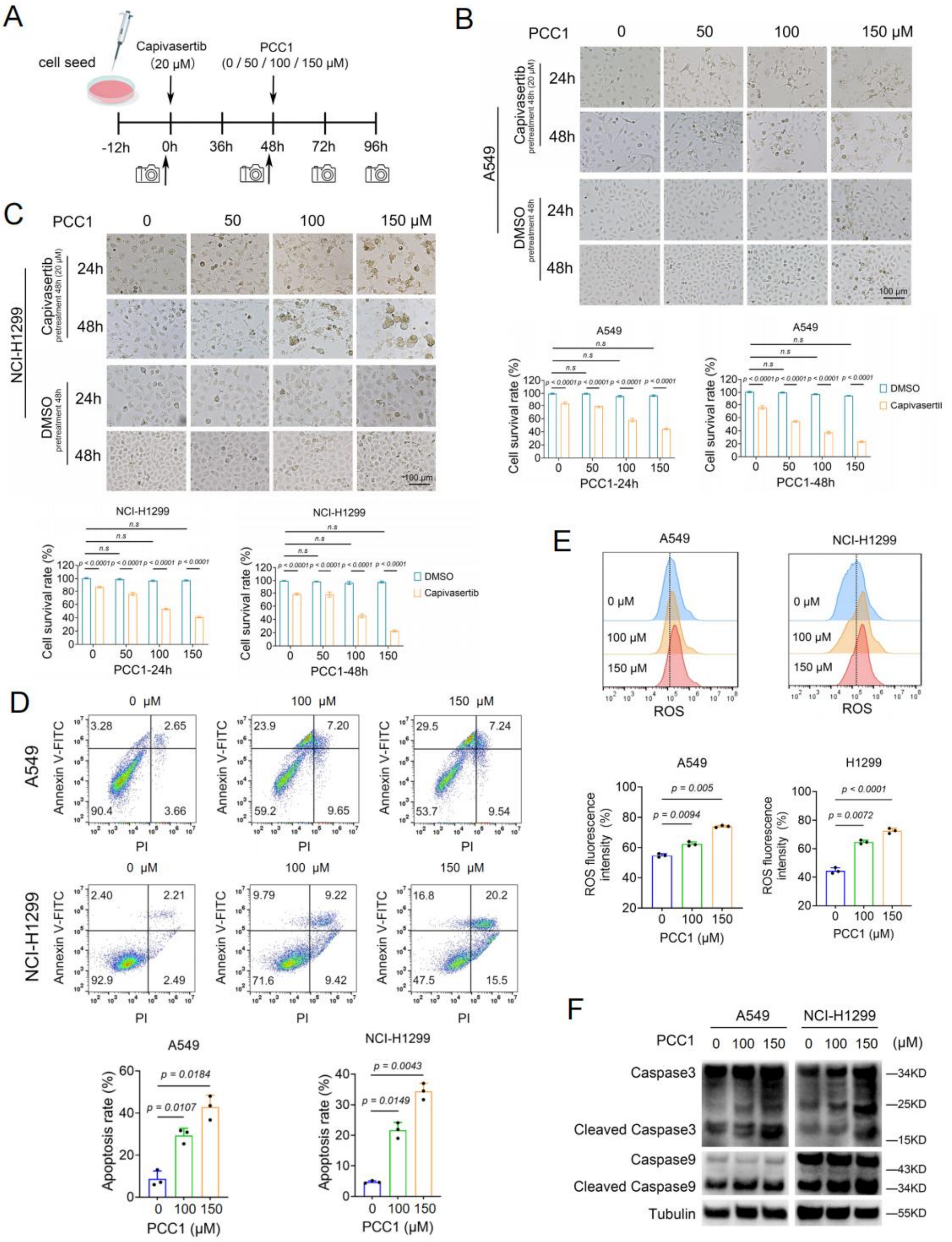
A
cell seed
Capivasertib
(20 μM)
PCC1
(0 / 50 / 100 / 150 μM)
-12h
0h
36h
48h
72h
96h
B
PCC1
0
50
100
150 μM
Capivasertib
24h
48h
A549
DMSO
C
NCI-H1299
D
A549
NCI-H1299
Annexin V-FITC
PI
0 μM
100 μM
150 μM
Apoptosis rate (%)
PCC1 (μM)
p = 0.0184
p = 0.0107
p = 0.0043
p = 0.0149
E
ROS
ROS fluorescence intensity (%)
p = 0.005
p = 0.0094
p < 0.0001
p = 0.0072
H1299
F
Caspase3
Cleaved Caspase3
Caspase9
Cleaved Caspase9
Tubulin
—34KD
—25KD
—15KD
—43KD
—55KD
Cell survival rate (%)
PCC1-24h
PCC1-48h
DMSO
Capivasertib

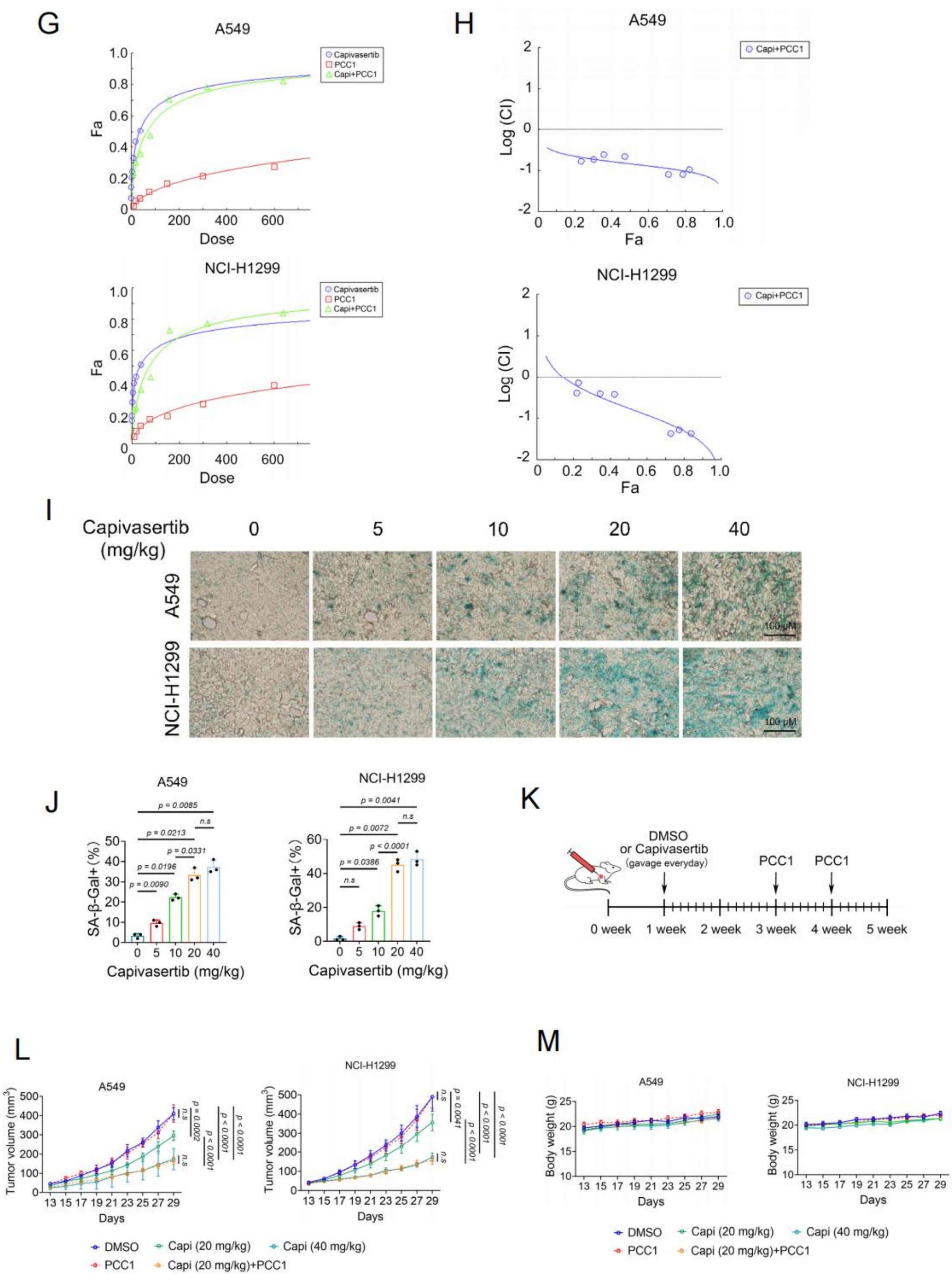

Supplementary Figure S9 | Synergistic effects of Capivasertib and PCC1 combination therapy *in vitro* and *in vivo*.

(A) Schematic of combination treatment protocol using Capivasertib (20 μM) with PCC1 (0/50/100/150 μM) in cellular models. (B, C) Dose- and time-dependent growth inhibition by Capivasertib (20 μM) combined with PCC1 (0-200 μM) in A549

(B) and NCI-H1299 (C) cells. (D) Annexin V/PI flow cytometry showing PCC1-induced apoptosis in senescent A549 and NCI-H1299 cells. (E) Flow cytometry showing dose-dependent mitochondrial ROS increase upon PCC1 treatment in senescent cells. (F) Western blot showing PCC1-induced cleavage of caspase-3 and caspase-9 in senescent cells. (G, H) Combination index (CI) analysis confirming synergistic cytotoxicity of capivasertib and PCC1. (G) Dose-response curves of cells treated with capivasertib alone, PCC1 alone, or their combination (48 h) by MTT assay. (H) Fa-log(CI) plots generated by CompuSyn (Chou-Talalay method). Log(CI) < 0, $= 0$, or > 0 indicated synergy (CI < 1), additivity (CI $= 1$), or antagonism (CI > 1), respectively. Consistent negative log(CI) values across multiple Fa levels confirmed synergy. (I, J) β-Galactosidase staining of frozen xenograft tumor sections showing dose-dependent senescence induction by Capivasertib. (K) Schematic representation of the *in vivo* combination therapy of Capivasertib with PCC1 (20 mg/kg). (L, M) Enhanced therapeutic efficacy of sequential treatment (Capivasertib 20 mg/kg ×2 weeks → PCC1 20 mg/kg ×2 doses+Capivasertib 20 mg/kg/day ×2 weeks) showing comparable tumor suppression to Capivasertib monotherapy (40 mg/kg/day ×4 weeks) in both A549 and NCI-H1299 xenograft models. Tracking of tumor volume (L) and body weight (M) was shown. Data represented the mean + or ± SEM, and the *p* values were analyzed by one-way ANOVA (B, C, D, E,J, L).

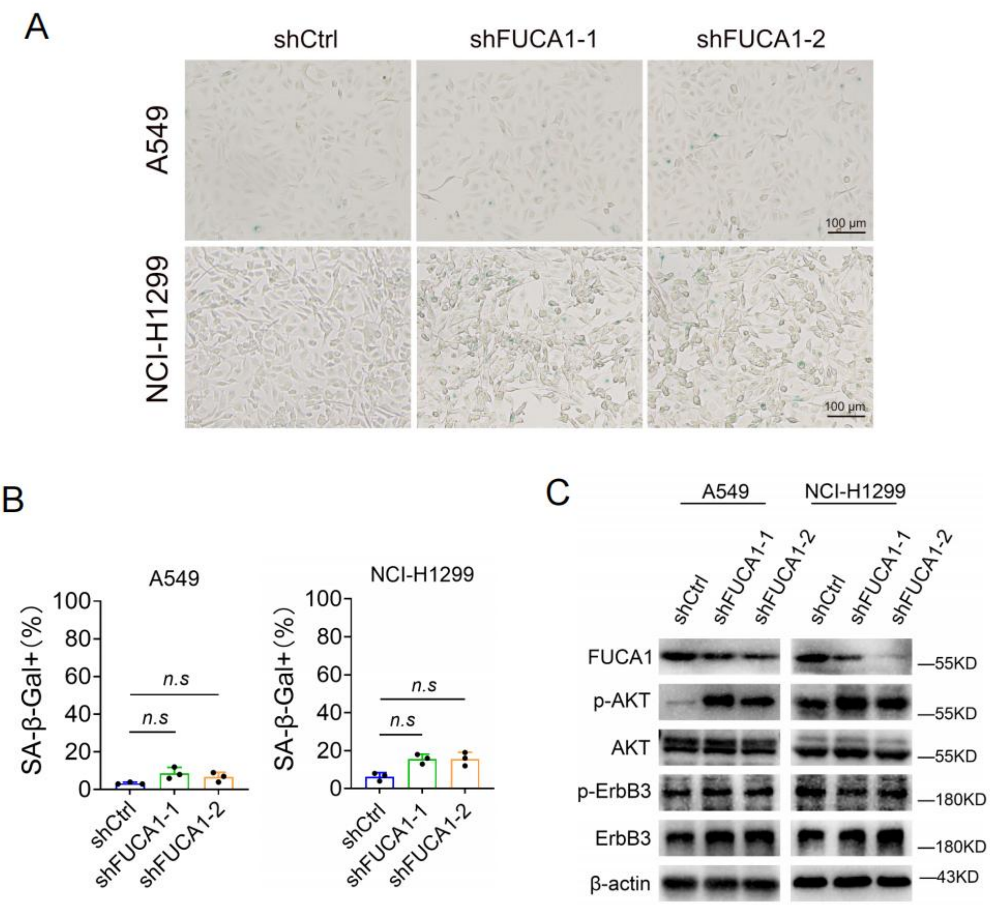

Supplementary Figure S10 | Effects of FUCA1 knockdown on ErbB3/AKT phosphorylation, cellular senescence, and SA-β-Gal staining in LUAD cells.

(A) Western blot showing that FUCA1 knockdown did not suppress AKT or ErbB3 phosphorylation. (B) SA-β-Gal staining showing that FUCA1 knockdown did not induce cellular senescence. (C) Quantification of SA-β-Gal-positive cells.

Supplementary Table

Supplementary Table S1 | Germline variants and genes associated with somatic mutational processes

Germline locus	ID	eQTL gene	Somatic mutational signature	Beta	Pvalue
**RS6937128**	6:144334264:A:G	FUCA2	aging-related	-0.43782	2.19E-06
**RS56360488**	6:144333407:A:C	FUCA2		-0.44484	2.55E-06
**RS76311854**	6:144332295:C:G	FUCA2		-0.42677	7.80E-06
**RS62427138**	6:144377479:C:T	FUCA2		-0.68724	5.67E-06
**RS12528481**	6:144334771:A:T	FUCA2		-0.44484	2.55E-06
**RS9766199**	6:144335819:T:C	FUCA2		-0.44242	2.79E-06
**RS6937531**	6:144334195:G:T	FUCA2		-0.43164	5.10E-06
**RS73006291**	6:144336820:G:A	FUCA2		-0.43395	4.39E-06
**RS3808523**	8:23162133:G:A	LOXL2		-0.45198	2.87E-06
**RS11135723**	8:23161204:A:C	LOXL2		-0.45955	4.32E-06
**RS12503646**	4:4711863:C:T	LYAR		-0.46313	9.98E-06
**RS72857966**	11:9150552:G:C	RIC3		0.600819	7.32E-06
**RS72857952**	11:9131489:C:A	RIC3		0.618573	5.87E-06
**RS1010806**	1:7153973:A:G	ZBTB48	tobacco exposure-related	0.292228	7.15E-06
**RS1010807**	1:7153864:T:C	ZBTB48		0.297075	5.52E-06
**RS61655518**	2:144134490:A:C	ARHGAP15	defective DNA mismatch repair-related	-0.52445	3.71E-06
**RS73964503**	2:144134540:T:C	ARHGAP15		-0.52445	3.71E-06
**RS2381468**	2:144136116:G:A	ARHGAP15		-0.52101	3.74E-06
**RS4939338**	11:60099225:T:C	MS4A2	POLE domain mutations-related	-0.53578	6.73E-06
**RS55777218**	11:60070946:T:C	MS4A2		0.528442	9.46E-06
**RS142761685**	6:81560733:A:Gs	TTK		1.040431	7.67E-06

Supplementary Material Tables

Supplementary Material Table S1 | Summary of antibodies

Name	Cat#	Clone No.	Source	Use	Dilution
β-actin	A3854		Sigma-Aldrich	WB	1: 10000
Flag	F1804		Sigma-Aldrich	WB	1: 10000
FUCA2	AM1909b		Abcepta	WB/IF	1: 500/1:100
AKT	4691	C67E7	Cell Signaling Technology	WB	1: 1000
p-AKT(Thr473)	4060	D9E	Cell Signaling Technology	WB	1: 1000
Rb	9309	4H1	Cell Signaling Technology	WB	1: 1000
p-Rb (Ser807/811)	8516	D20B12	Cell Signaling Technology	WB	1: 1000
p-EGFR (Tyr1068)	3777	D7A5	Cell Signaling Technology	WB	1: 1000
ErbB3/HER3	12708	D22C5	Cell Signaling Technology	WB	1: 1000
p-ErbB3 (Tyr11289)	4791	21D3	Cell Signaling Technology	WB	1: 1000
ErbB2/HER2	2165	29D8	Cell Signaling Technology	WB	1: 1000
EGFR	4267	D38B1	Cell Signaling Technology	WB/IF	1: 1000/1:200
$p27^{Kip}$	3686	D69C12	Cell Signaling Technology	WB/IF	1: 1000/1:200
LAMP-2	sc-18822	H4B4	Santa Cruz Biotechnology	WB/IF	1: 500/1: 200
p53	sc-126	DO-1	Santa Cruz Biotechnology	WB/IF	1: 500:1:200
Skp2	sc-74477	A-2	Santa Cruz Biotechnology	WB	1: 1000
MDM2	sc-965	SMP14	Santa Cruz Biotechnology	WB	1: 1000
Lamin B1	sc-6216		Proteintech group	WB	1: 1000
LAMP1	21997-1-AP		Proteintech group	WB	1: 1000
p21	10355-1-AP		Proteintech group	WB/IF	1: 1000/1:200
p16-INK4A	10883-1-AP		Proteintech	WB	1: 1000

			group		
AAL	B-1395-1		VectorLabs	WB	1: 1000
LTL	B-1325-2		VectorLabs	WB	1: 500
LCA	B-1045-5		VectorLabs	WB	1: 1000
UEA-1	B-1065-2		VectorLabs	WB	1: 1000
Goat anti-Rabbit IgG (H+L) Cross-Adsorbed Secondary Antibody, Alexa Fluor™ 488	A-11008		Invitrogen	IF	1: 200
Goat anti-Mouse IgG (H+L) Cross-Adsorbed Secondary Antibody, Alexa Fluor™ 488	A-11001		Invitrogen	IF	1: 200
Goat anti-Rabbit IgG (H+L) Cross-Adsorbed Secondary Antibody, Alexa Fluor™ 594	A-11012		Invitrogen	IF	1: 200
Goat anti-Mouse IgG (H+L) Cross-Adsorbed Secondary Antibody, Alexa Fluor™ 594	A-11005		Invitrogen	IF	1: 200

Supplementary Material Table S2 | Summary of chemical compounds

Name	Cat#	Source	Solvent
Capivasertib	HY-15431	MedChemExpress	DMSO
MK-2206	HY-108232	MedChemExpress	DMSO
PCC1	HY-N2342	MedChemExpress	Sterile deionized water
MG132	HY-13259	MedChemExpress	DMSO
CQ	HY-17589A	MedChemExpress	DMSO
Tunicamycin	HY-A0098	MedChemExpress	DMSO
recombinant human FUCA2 protein	ab187614	Abcam	Sterile deionized water

Supplementary Material Table S3 | Summary of real-time PCR primers

Gene	Primer type	Company	Sequence (5'-3')	Note
FUCA2-F	SYBR Green	Sangon	GAGGTTCTGTGGTCGGATGG	synthesized oligo
FUCA2-R	SYBR Green	Sangon	GCCACCATGCTTACAGATGC	synthesized oligo
TP21-F	SYBR Green	Sangon	GGCGGCAGACCAGCATGACAGATT	synthesized oligo
TP21-R	SYBR Green	Sangon	GCAGGGGGCGGCCAGGGTAT	synthesized oligo
TP53-F	SYBR Green	Sangon	CCTCAGCATCTTATCCGAGTGG	synthesized oligo
TP53-R	SYBR Green	Sangon	TGGATGGTGGTACAGTCAGAGC	synthesized oligo
TP27-F	SYBR Green	Sangon	CCGGTGGACCACGAAGAGT	synthesized oligo
TP27-R	SYBR Green	Sangon	GCTCGCCTCTTCCATGTCTC	synthesized oligo
GAPDH-F	SYBR Green	Sangon	CACCATCTTCCAGGAGCGAG	synthesized oligo
GAPDH-R	SYBR Green	Sangon	GACTCCACGACGTACTCAGC	synthesized oligo
IL6-F	SYBR Green	Sangon	GACAGCCACTCACCTCTTCA	synthesized oligo
IL6-R	SYBR Green	Sangon	GACAGCCACTCACCTCTTCA	synthesized oligo
IL8-F	SYBR Green	Sangon	GCTCTGTGTGAAGGTGCAGTTTTGCCAA	synthesized oligo
IL8-R	SYBR Green	Sangon	GGCGCAGTGTGGTCCACTCTCAAT	synthesized oligo
TNFα-F	SYBR Green	Sangon	CCCAGGCAGTCAGATCATCTTCTCGAA	synthesized oligo
TNFα-R	SYBR Green	Sangon	CTGGTTATCTCTCAGCTCCACGCCATT	synthesized oligo
ERBB3-F	SYBR Green	Sangon	AGGGACCCAGGTCTACGATG	synthesized oligo
ERBB3-R	SYBR Green	Sangon	CTCACGATGTCCCTCCAGTC	synthesized oligo
FUT3-F	SYBR Green	Sangon	CCGACTACATCACCGAGAAGCT	synthesized oligo
FUT3-R	SYBR Green	Sangon	GAACCTCTCGTAGTTGCTTCTGC	synthesized oligo
FUT8-F	SYBR Green	Sangon	GACAGAACTGGTTCAGCGGAGA	synthesized oligo
FUT8-R	SYBR Green	Sangon	GCAGTAGACCACATGATGGAGC	synthesized oligo

Supplementary Material Table S4 | Summary of oligos for shRNAs

Gene	Source	Sequence (5'-3')	Note
shControl	Sangon	CAACCCGCTCCAAGGAATCG	synthesized oligo
shFUCA2-1	Sangon	AGTCAATGGAGAAGCTATTTA	synthesized oligo
shFUCA2-2	Sangon	ATTACCCTCCTAGTTTCAAAT	synthesized oligo
shp53-1	Sangon	TATCCGAGTGGAAGGAAATTT	synthesized oligo
shp53-2	Sangon	CCTCTGAGTCAGGAAACATTT	synthesized oligo
shp21-1	Sangon	GCAGACCAGCATGACAGATTT	synthesized oligo
shp21-2	Sangon	AGCGATGGAACTTCGACTTTG	synthesized oligo
shp27-1	Sangon	CGGTGGACCACGAAGAGTTAA	synthesized oligo
shp27-2	Sangon	GAGCCAGCGCAAGTGGAATTT	synthesized oligo
shFUT1-1	Sangon	ACAGTGCAACCACACCATTAT	synthesized oligo
shFUT1-2	Sangon	CTGAGTTCCTGAAGATCTTTA	synthesized oligo
shFUT2-1	Sangon	CTTTACGGTTTCCACTATATT	synthesized oligo
shFUT2-2	Sangon	GGGCTCACCTGCCAAAGATTT	synthesized oligo
shFUT3-1	Sangon	TGTCTGGCCGCACTGCTATTT	synthesized oligo
shFUT3-2	Sangon	CATCGTGCACCACTGGGATAT	synthesized oligo
shFUT8-1	Sangon	CAGAGTGAATTGAAGAAATTA	synthesized oligo
shFUT8-2	Sangon	ACATCATGAAAGGTCTATAAT	synthesized oligo
shERBB3-1	Sangon	GTGCTGGGCGTATCTATATAA	synthesized oligo
shERBB3-2	Sangon	GTGCTTGTCCTGTCGAAATTA	synthesized oligo
